# Comprehensive genomic profiling of colorectal cancer patients reveals differences in mutational landscapes among clinical and pathological subgroups

**DOI:** 10.3389/fonc.2022.1000146

**Published:** 2022-11-10

**Authors:** Peng Li, Qingyu Meng, Yonggan Xue, Zhipeng Teng, Hanlin Chen, Junli Zhang, Yang Xu, Sha Wang, Ruoying Yu, Qiuxiang Ou, Xue Wu, Baoqing Jia

**Affiliations:** ^1^ Department of General Surgery, The First Medical Centre, Chinese People’s Liberation Army (PLA) General Hospital, Beijing, China; ^2^ Geneseeq Research Institute, Geneseeq Technology Inc., Nanjing, Jiangsu, China

**Keywords:** Colorectal cancer, next generation sequencing - NGS, biomarkers, clinicopathological analysis, genomic analysis

## Abstract

With the widespread of colonoscopy, colorectal cancer remains to be one of the most detrimental types of cancer. Though there were multiple studies investigating the genomic landscape of colorectal cancer, a comprehensive analysis uncovering the differences between various types of colorectal cancer is still lacking. In our study, we performed genomic analysis on 133 patients with colorectal cancer. Mutated *FAT1* and *PKHD1* and altered Hippo pathway genes were found to be enriched in early-onset colorectal cancer. APOBEC signature was prevalent in microsatellite stable (MSS) patients and was related to lymph node metastasis. *ZNF217* mutations were significantly associated with early-stage colorectal cancer. In all, this study represents a comprehensive genomic analysis uncovering potential molecular mechanisms underneath different subgroups of colorectal cancer thus providing new targets for precision treatment development.

## Introduction

The incidences of colorectal cancer drop significantly after the widespread of colonoscopy, which enabled the early detection of colorectal cancer and decreased the mortality rate (1). Even with the modern early-detection technology, however, colorectal cancer remained the third most diagnosed cancer worldwide and was ranked to have the fourth-highest incidence rate and fifth-highest mortality rate among all cancer types in China (2). It has incurred a huge economic loss for patients and communities.

Colorectal cancer has multiple risk factors, including age, sex, genetic factors, etc., while over half of the cases were ascribed to modifiable and preventable factors, such as unhealthy lifestyles (3). Though the incidence rate increases dramatically with age, a widespread rise in early-onset colorectal cancer cases is noticed (4). Moreover, previous studies reported that different types of colorectal cancer (early stage vs. late stage, microsatellite instability vs. microsatellite stable) displayed different prognosis performances (5). Multiple genetic alternations are considered to play important roles in colorectal cancer development, such as *APC, KRAS*, and *TP53* (6–8). However, while there are multiple studies that reported the genomic meta-analysis in colorectal cancer patients, studies comparing the mutational landscape to illustrate the differences in clinical outcomes between different clinical or pathological colorectal cancer subgroups are still lacking.

In this study, we performed genomic analysis for 133 colorectal cancer patients using targeted sequencing with a 425 cancer-related gene panel. The genomic landscape of different subgroups of colorectal cancer patients was compared, including early versus late-onset, MSI versus MSS, and different anatomic locations, which has substantially expanded our understanding of molecular mechanisms underlying colorectal cancer.

## Materials and methods

### Patient cohort

A total of 133 colorectal cancer (CRC) patients were retrospectively recruited from the Chinese PLA General Hospital. Tumor tissue was sampled from each patient before treatment. This study was approved by the Ethical Committee of Chinese PLA Central Hospital (Approval No. S2022-307-01). The patients/participants provided their written informed consent to participate in this study. Targeted sequencing of 425 cancer-related genes was performed on both the tumor tissue sample and the matched white blood cell sample from each patient (gene list, **Table S1**), and the sequencing results from the white blood cells were used as controls to filter out the germline mutations. The resulting tumor somatic mutations were listed in **Table S2**. The results derived from our patient cohort were further validated using a published independent dataset consisting of 240 stage II to III Chinese colorectal patients (9). Detailed clinicopathological features of the validation cohort can be found in **Table S5**.

### DNA extraction and sequencing library preparation

As previously described (10), the genomic DNA from formalin‐fixed and paraffin‐embedded (FFPE) was extracted using the QIAamp DNA FFPE Tissue Kit (Qiagen) according to the manufacturer’s protocol. The quantity and quality of the extracted DNA were evaluated using a Qubit 3.0 fluorometer and Nanodrop 2000, respectively (Thermo Fisher Scientific). Sequencing libraries were prepared using the KAPA Hyper Prep Kit (KAPA Biosystems) according to the manufacturer’s suggestions for different sample types. In brief, 1 μg of fragmented genomic DNA underwent end-repairing, A-tailing, and ligation with indexed adapters sequentially, followed by size selection using Agencourt AMPure XP beads (Beckman Coulter). Hybridization-based target enrichment was carried out with a pan-cancer gene panel (474 cancer-relevant genes), and xGen Lockdown Hybridization and Wash Reagents Kit (Integrated DNA Technologies). Captured libraries by Dynabeads M-270 (Life Technologies) were amplified in KAPA HiFi HotStart ReadyMix (KAPA Biosystems) and quantified by qPCR using the KAPA Library Quantification Kit (KAPA Biosystems) for sequencing.

### Next generation sequencing

Sequencing data were processed as previously described (10). In brief, the data was first demultiplexed and subjected to FASTQ file quality control to remove low-quality data or N bases. Qualified reads were mapped to the reference human genome hg19 using Burrows-Wheller Aligner and Genome Analysis Toolkit (GATK 3.4.0) was employed to apply the local realignment around indels and base quality score recalibration. Picard was used to remove PCR duplicates. VarScan2 was employed for the detection of single-nucleotide variations (SNVs) and insertion/deletion mutations. SNVs were filtered out if the mutant allele frequency (MAF) was less than 1% for tumor tissue and 0.3% for plasma samples. Common SNVs were excluded if they were present in >1% population in the 1000 Genomes Project or the Exome Aggregation Consortium (ExAC) 65,000 exomes database. The resulting mutation list was further filtered by an in-house list of recurrent artifacts based on a normal pool of whole blood samples. Parallel sequencing of matched white blood cells from each patient was performed to further remove sequencing artifacts, germline variants, and clonal hematopoiesis. The Copy number alterations were analyzed as previously described (11, 12). The tumor purities were first estimated using ABSOLUTE (13). Somatic CN alteration events were assigned based on sample-ploidy values calculated in the FACETS algorithm. Structural variants were detected using FACTERA with default parameters (14). The fusion reads were further manually reviewed and confirmed on Integrative Genomics Viewer (IGV).

### Data analysis

Statistical analyses were performed using the R (v3.4.2), and a *P*-value <0.05 (*) was considered to be statistically significant. To define the mutational signatures, we assessed the mutational context of nonsynonymous SNVs in tumor samples with at least 5 mutations (n=133). The mutational patterns were compared to the mutational signatures reported by Alexandrov et al. (15). All mutational signatures were confirmed using deconstructSigs with default parameters (16).

## Results

### The clinical features of the analyzed cohort

This analyzed CC cohort included 75 males (56.39%) and 58 females (43.61%) with a median age at diagnosis of 58 years old, ranging from 29 to 85 years old (**Table 1**). More than a quarter of the patients were early-onset colorectal cancer with an age below 50 (25.57%). There were 2 (1.50%) stage 0, 18 (13.53%) stage I, 42 (31.58%) stage II, 59 (44.36%) stage III patients, and 12 (9.02%) stage IV patients. Around 96% (128/133) of the total cases were adenocarcinoma and 3.76% (5/133) were other histological subtypes. Each patient had one tumor sample, resulting in a total of 133 colorectal tumors. The tumor location included right-side colon (31, 23.31%), left-side colon (36, 27.07%), and rectal (66, 49.62%). 53 (39.85%) tumors had a tumor size larger than 5cm and 76 (57.14%) tumors had a tumor size less than 5cm. 12 tumors (9.02%) were identified as microsatellite instability (MSI)-high. The histological grades of tumors were well-differentiated (2, 1.50%), moderate-differentiated (114, 85.71%), poor-differentiated (8, 6.01%), or unknown (9, 6.77%). The distribution of different tumor locations, tumor volumes, and stages were well-balanced between males and females and between early-onset (<50) and late-onset (≥50) patients (**Table S3**).

**Table 1:** The clinical features of enrolled patients.

Features	No. of patients (percentage)
**Sex**	
** Male**	75 (56.39%)
** Female**	58 (43.61%)
**Age**	
** ≥50**	99 (74.43%)
** <50**	34 (25.57%)
**Median age**	58 (29~85)
**Primary tumor location**	
** Right-side colon**	31 (23.31%)
** Left-side colon**	36 (27.07%)
** Rectal**	66 (49.62%)
**Tumor size**	
** ≥5 cm**	53 (39.85%)
** <5 cm**	76 (57.14%)
** Unknow**	4 (3.01%)
**MS status**	
** MSS**	121 (90.98%)
** MSI-high**	12 (9.02%)
**Pathological stage**	
** 0**	2 (1.50%)
** I**	18 (13.53%)
** II**	42 (31.58%)
** III**	59 (44.36%)
** IV**	12 (9.02%)
**Histological type**	
** Adenocarcinoma**	128 (96.24%)
** other**	5 (3.76%)
**Histological grade**	
** Well-differentiated**	2 (1.50%)
** Moderate-differentiated**	114 (85.71%)
** Poor-differentiated**	8 (6.01%)
** Unknow**	9 (6.77%)

MSS, microsatellite stability; MSI, microsatellite instability.

### Altered Hippo pathway enriched in early-onset colorectal cancer patients

**Figure 1:**
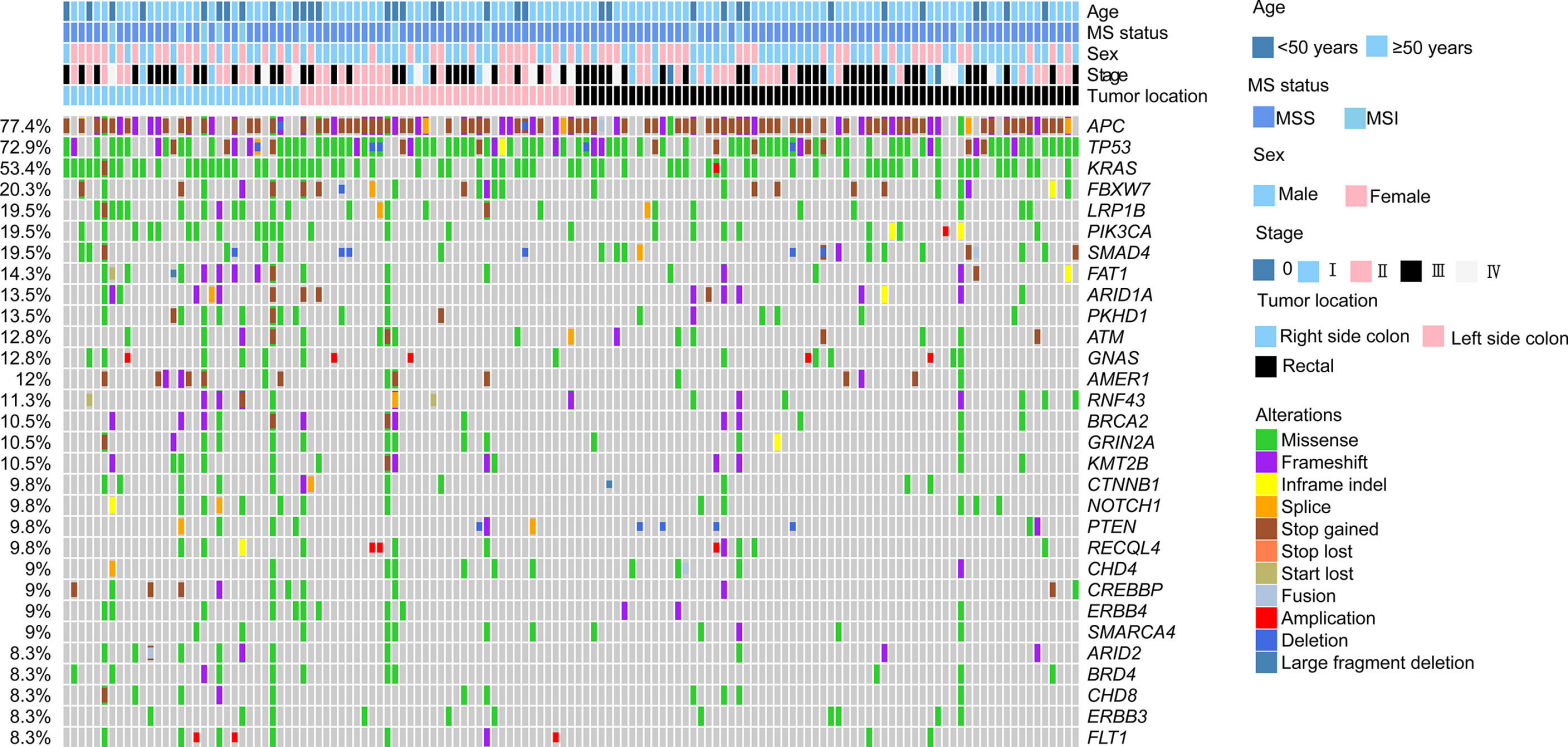
Genomic landscape of colorectal cancer patients. The clinicopathological features including age, sex, stage, tumor location, and MS status were indicated by the bar on the top. The types of alterations were indicated by different colors. Each column represented one patient.

**Figure 2:**
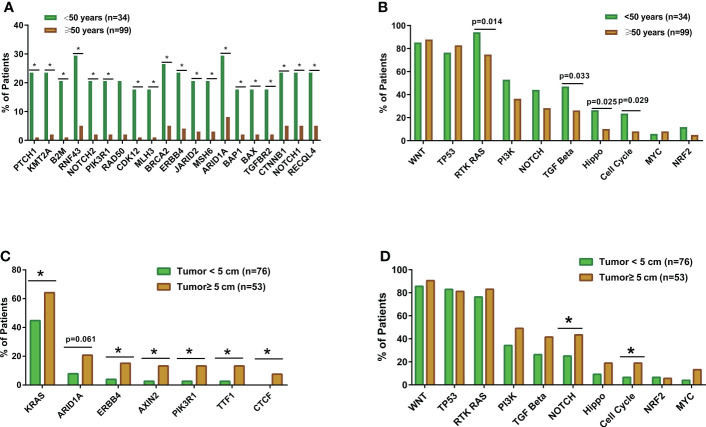
The comparisons of somatic mutation and pathway alteration characteristics between colorectal cancer patients with different tumor sizes and ages. **(A)** The bar plots comparing gene alteration rates in early-onset (N=34) and late-onset patients (N=99). **(B)** The bar plots comparing pathway alteration rates in early-onset (N=34) and late-onset patients (N=99). **(C)** The bar plots comparing gene alteration rates patients with small tumor size (tumor diameter smaller than 5cm, N=76) and large tumor size (tumor diameter equal or larger than 5cm, N=53). **(D)** The bar plots comparing pathway alteration rates patients with small tumor size (tumor diameter smaller than 5cm, N=76) and large tumor size (tumor diameter equal or larger than 5cm, N=53). *p < 0.05.

The genomic landscape of the overall colorectal cancer cohort was shown in **Figure S1**. In the enrolled patients, the most frequently mutated genes were *APC* (77.4%), *TP53* (72.9%), *KRAS* (53.4%), and *FBXW7* (20.3%) (**Figure 1**). We then compared the mutation profiles between early- and late-onset CC patients. As shown in **Figure 2A**, alterations in multiple genes, including *PTCH1*, *KMT2A*, *B2M*, *RNF43*, *NOTCH2*, and *PIK3R1*, were significantly enriched in the early-onset patients when compared with the late-onset patients. Similar trends of the enrichment of somatic alterations in *CDK12, PTCH1, PIK3R1, ERBB4, BRCA2, RAD50, etc.* were also observed in the validation cohort, though the results did not reach statistical significance (**Figure S1A**). Pathway analysis revealed that the majority of gene alterations enriched in the younger population were in the Hippo pathway (*P*=0.025), cell cycle pathway (*P*=0.029), and TGFβ pathway (*P*=0.033) (**Figure 2B**). The results on Hippo and cell cycle pathways were further recapitulated in the validation cohort (**Figure S1B**). Additionally, the Hippo pathway alterations and early-onset disease showed significant associations using both univariate and multivariate analyses (**Table 2**). We further compared the genomic profiles of small (<5cm) and large (≥5cm) tumors. As for individual genes, alterations in *KRAS, ERBB4, AXIN2, PIK3R1, TTF1, and CTCF* displayed a significant difference between large and small tumors (**Figure 2C**). Notch and cell cycle pathway gene alterations were significantly enriched in the tumors with a size larger than 5cm (*P*<0.05, **Figure 2D**); however, the results were insignificant in multivariate analysis (**Table 3**).

**Table 2:** Univariant and multivariant analysis of patients stratified by young (<50) and old (≥50).

Factors	Univariate analysis HR (95%CI)	P value	Multivariate analysis HR (95%CI)	P value
Stage	–	0.424	0.66 (0.43~1.02)	0.063
Tumor location	–	0.982	0.69 (0.26~1.78)	0.439
Tumor size	0.85 (0.41~1.73)	0.721	0.93 (0.44~1.98)	0.849
Hippo pathway	0.27 (0.06~0.91)	0.025	0.29 (0.08~0.99)	0.048

**Table 3:** Univariant and multivariant analysis of patients stratified by small (<5cm) and large (≥5cm) tumor sizes.

Factors	Univariate analysis HR (95%CI)	P value	Multivariate analysis HR (95%CI)	P value
Age	1.00 (0.39~2.26)	0.929	1.29 (0.54~3.25)	0.574
Stage	–	0.189	1.23 (0.58~2.69)	0.592
Tumor location	–	0.008	0.37 (0.12~1.02)	0.059
NOTCH pathway	2.28 (1.01~5.22)	0.036	2.33 (0.67~8.84)	0.191
Cell cycle pathway	3.27 (0.94~13.04)	0.049	1.94 (0.855~4.44)	0.116

### APOBEC signature enriched in the MSS patients

As for the molecular features of MSS and MSI patients, MMR gene alterations including MSH6, MLH1/3, MSH2, PMS1/2 were significantly higher in the MSI group. Furthermore, the mutational signature analysis showed that MMR deficiency signature was increased in the MSI group while APOBEC signature was higher in the MSS group (**Figures S2A, B**). This enrichment of APOEBC signature in MSS patients with colorectal cancer was also observed in the validation cohort (**Figure S2C**).

### ZNF217 alterations associated with early-stage CRC

**Figure 3:**
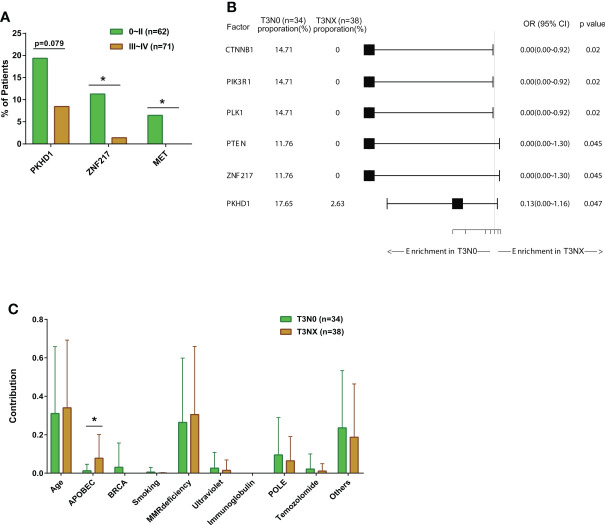
The comparisons of somatic mutation characteristics and signatures among colorectal cancer patients with different stages. **(A)** The bar plots comparing gene alteration rates in early- (Stage 0~II, N=62) and late-stage patients (Stage III~IV, N=71). **(B)** Forest plot of somatic mutation rates of stage III colorectal cancer patients with metastatic disease (T3N0, N=34) compared to patients without metastatic disease (T3NX, N=38). **(C)** The bar plots comparing somatic mutation signatures between patients with metastatic disease (T3N0, N=34) and patients without metastatic disease (T3NX, N=38). *p < 0.05.

We compared the mutation profiles of early-stage (0-II) and late-stage (III-IV) CRC patients. The ratios of *ZNF217* alterations (*P*<0.05), *MET* alterations (*P*<0.05), and *PKHD1* alterations (*P*=0.079) were higher in the early-stage group (**Figure 3A**). The univariate and multivariate analysis also identified altered *ZNF217* as an independent factor associated with early stage (**Table 4**). The forest plot further revealed the enrichment of *ZNF217* (*P*=0.045) and *PKHD1* (*P*=0.047) alterations in stage T3N0 patients but not in T3NX patients (**Figure 3B**). The T3NX stage displayed an association with a higher APOBEC signature compared to T3N0, suggesting the lymph node metastasis in colorectal cancer may be related to the APOBEC signature (**Figure 3C**).

**Table 4:** Univariant and multivariant analysis of patients stratified by early-stage (stage0-II) and late-stage (stage III-IV).

Factors	Univariate analysis HR (95%CI)	P value	Multivariate analysis HR (95%CI)	P value
Age	0.63 (0.26~1.50)	0.320	0.63 (0.27~1.43)	0.278
Tumor location	–	0.911	0.96 (0.35~2.62)	0.938
ZNF217	0.11 (0.00~0.93)	0.025	0.11 (0.01~0.64)	0.041

### Anatomic location and genomic features of CRC

**Figure 4:**
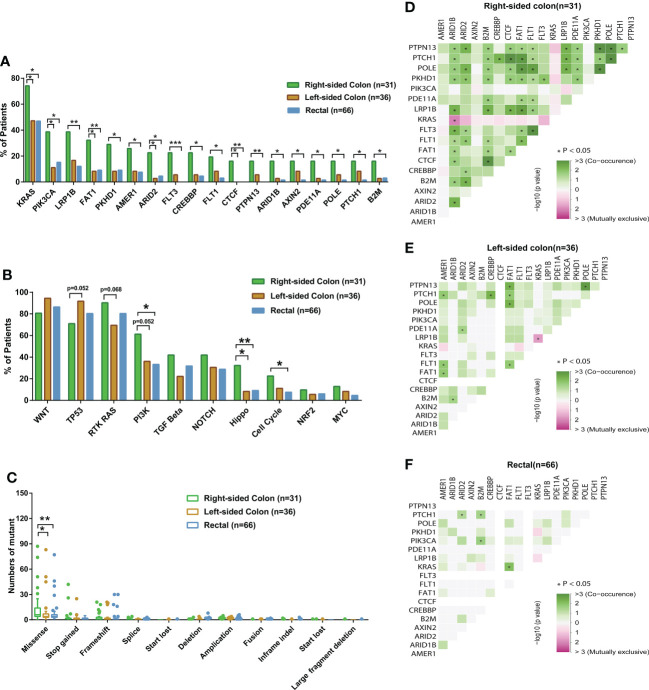
The comparisons of somatic mutation characteristics and mutation types among colorectal cancer patients with different tumor anatomic locations and genetic interaction analysis of different tumor anatomic locations. **(A, B)** The bar plots comparing somatic mutation and pathway alteration rates among patients with different tumor anatomic locations (right-sided colon, N=31; left-sided colon, N=36; rectal, N=66). **(C)** The box plots comparing differences in mutation types among patients with different tumor anatomic locations (right-sided colon, N=31; left-sided colon, N=36; rectal, N=66). **(D-F)** Genetic interaction analysis of different tumor anatomic locations. *p < 0.05, **p < 0.01, ***p < 0.001.

**Figure 5:**
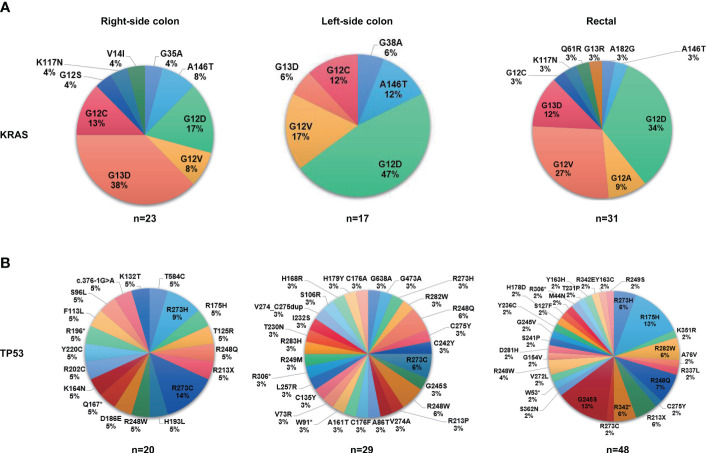
The proportions of mutation sites of KRAS and TP53 in different tumor anatomic locations. **(A)** The proportions of mutation sites of KRAS in different tumor anatomic locations (right-sided colon, N=31; left-sided colon, N=36; rectal, N=66). **(B)** The proportions of mutation sites of TP53 in different tumor anatomic locations (right-sided colon, N=31; left-sided colon, N=36; rectal, N=66).

Next, we investigated the genomic features of tumors in different anatomic locations. Tumors in the right-sided colon displayed a significantly higher ratio of altered genes including *KRAS*, *PIK3CA*, *LRP1B*, *FAT1*, and *PKHD1* (**Figure 4A**), and a considerable number of these right-sided tumor-enriched genetic alterations (e.g., *KRAS, PIK3CA, CREBBP, PKHD1, AMER1, FAT1, ARID2*, and *POLE*) were further confirmed in the validation cohort (**Figure S3A**). Additionally, the Hippo pathway, cell cycle pathway, and PI3K pathway were more frequently altered in the tumors of the right-sided colon compared to the tumors of the left-sided colon and rectal (**Figure 4B**), which were all confirmed in the validation cohort (**Figure S3B**). Meanwhile, higher missense mutations were found in the right-sided colon tumors compared to the other two locations (**Figure 4C**). In the right-sided colon tumors, *PKHD1* co-occurred with *ARID1B, ARID2, B2M, CTCF, FAT1, FLT1, FLT3*, and *PDE11A*, most of which were DNA damage genes, whereas *KRAS* was found mutually exclusive with *ARID1B* (**Figure 4D**). On the other hand, *KRAS* was mutually exclusive with *LRP1B* in the left-side colon tumors (**Figure 4E**), while the result was not significant in other sites (**Figures 4D,F**). We further investigated the distribution of frequently mutated genetic alterations in tumors from different anatomic locations. As shown in **Figure 5A** and **Table S4**, the rectal tumors displayed a higher ratio of *KRAS* G12 mutations (G12D 34%, G12V 27%, G12A 9%, G12C 3%) compared to the tumors from the colon. The distribution of *TP53* mutation was similar among right-side colon, left-sided colon, and rectal tumors (**Figure 5B**).

## Discussion

Understanding the differences in the genomic landscape among various colorectal cancer subgroups is important for the development of precision care. The present study revealed the subgroup-specific genetic alterations in colorectal cancer. In particular, we demonstrated the differentially enriched mutations and aberrant signaling pathways between early- and late-onset patients, as well as between early- and late-stage diseases. Additionally, a distinct mutational landscape was observed between right-sided and left-sided colon cancers, and we found that the tumor mutational spectrum differed according to the lymphatic metastatic status for T3 stage colorectal cancers. These findings are particularly important in understanding the underlying mechanisms of tumorigenesis and metastasis of colorectal carcinoma, as well as contributing to implementing precise medication.

We investigated the molecular features of 133 colorectal cancer cases by comprehensive genomic profiling of 425 cancer-related genes. Consistent with previous research (17), *APC* (77.4%), *TP53* (72.9%), and *KRAS* (53.4%) represented the most frequently mutated genes in our cohort. Loss-of-function mutations of APC have been shown to induce the accumulation of β-catenin and activate TCF signaling pathway, thus promoting tumor evolution (6). *KARS* mutations, which could activate the RAS-RAF-MEK-ERK signaling pathway and stimulate cell proliferation (7), were found in most colorectal cancer cases (18) and were typically enriched in patients with bigger tumors than those in our study.

Additionally, we analyzed tumor location-based mutational discrepancies. Notably, we observed that mutations in *KRAS*, *PIK3CA*, *CREBBP*, *FAT1*, *PKHD1*, *ARID2*, and *POLE* were specifically enriched in right-site colon cancers in both our cohort and the validation cohort. Previous research has shown that DNA mismatch repair pathways frequently occurred in colon tumors located on the right side (19). Consistently, we found a higher percentage of missense mutations in right-site colon cancers, as compared with left-site colon cancers and rectal cancers. It is well studied that *KRAS* mutations were present in a majority of colorectal tumors (18). Intriguingly, our study discovered that *KRAS* mutational frequency was closely correlated with the tumor size, with larger tumors tending to enrich for *KRAS* mutations. Genetic alternations in *KRAS* could aberrantly activate the RAS-MAPK signaling axis, which may facilitate tumor initiation and promote early relapse after neoadjuvant chemotherapy (18). *KRAS* mutations were also important drug-resistant mechanisms for some targeted drugs like cetuximab and bevacizumab (7). Noticeably, we found that the incidence of *KRAS* mutations was higher in right-sided colon cancers than that in left-sided ones. A similar trend has been observed in previous studies, with *KRAS* mutational frequency between right- and left-sided colon cancers being 49.7% vs. 33.0% (20) or 40.0% vs. 29.8% (21). Intriguingly, these two studies also found that *BRAF* mutational frequency was significantly higher in right-sided colon cancers than in left-sided colon cancers. Given that *KRAS* and *BRAF* mutations are generally mutually exclusive (21), genetic alterations of *KRAS* and *BRAF* were likely to occur in different colon tumors, implying that right-sided colon tumors are predisposed to harbor KRAS-BRAF pathway mutations. Furthermore, we found that mutations of *KRAS* and *ARID1B* were also mutually exclusive in right-site cancer, but not in other sites. Sen et al. found that *ARID1A* mutations were significantly mutually exclusive with *KRAS* mutations using the TCGA colorectal cancer cohort (22). Considering the close relationship between *ARID1A* and *ARID1B (* 23), it would be compelling to investigate their tumor site-specific associations with KRAS pathway in future research. In addition, we discovered that *KRAS* was mutually exclusive with *LRP1B* in the left-sided colon tumors. Previous studies showed that the mutation of *EGFR* and *LRP1B* were mutually exclusive in primary lung adenocarcinoma (24), while no studies have reported the site-specific mutual exclusion between *KRAS* and *LRP1B*. Nevertheless, given the limited sample size in individual locations, future studies are warranted to validate our findings.

Besides tumor site-specific genetic features, we discovered that the frequency of the aberrant Hippo signaling pathway was higher in large tumors (≥5cm) than in small tumors (<5cm). Similarly, younger patients (aged ≤50 years) appeared to possess a higher percentage of genetic aberrances in the Hippo signaling pathway as compared with elder patients. Given that the Hippo pathway could inhibit the WNT signaling pathway (25), the aberrant Hippo signaling may result in WNT overactivation and consequently, expedite tumor growth. Consistent with this speculation, large tumors were typically accompanied by ischemia and hypoxia in the core region of the tumor, which, in turn, overacts the WNT pathway (26). Excessive activity in WNT signaling activates the stemness of colorectal cancer stem cells, leading to fast growth and resistance to chemotherapy (27).

Based on mutation signature analysis, we found that the APOEBC signature was enriched in colorectal cancer patients with MSS and/or lymphatic metastases. APOEBC signature, which is derived from APOBEC3A and APOBEC3B cytosine deaminase activities, is one of the most common mutation signatures in different types of cancer, and it has been reported to be associated with tumorigenesis and drug resistance (28). In particular, Law et al. reported that elevated APOBEC3A expression could promote tumor formation in colon and liver tissues in the mouse model (29). Additionally, APOBEC mutagenesis was often associated with higher intratumor heterogeneity, thus increasing tumor subclone diversity (30). The enrichment of APOEBC signature in T3NX patients in our cohort suggests that colorectal patients with APOEBC signature tend to have advanced disease and prone to lymph node metastasis.

Among patients with T3 stage tumors, we discovered that *CTNNB1* and *ZNF217* mutations were mainly present in patients who were absent of metastatic disease. Liu and colleagues reported that CTNNB1 (β-catenin), a core constituent of the WNT signaling pathway, could promote the development and progression of colorectal cancer and enhance the stemness of cancer stem cells (31). Amplification of ZNF217 is associated with advanced disease (32). The high expression of ZNF217 was reported to be associated with the poor prognosis and the development of metastases in breast cancer (33). However, the role of ZNF217 in colorectal cancer metastasis is still unknown.

There were several limitations in the present study. Firstly, the sample size of our patient cohort was intermediate, and it was relatively difficult to reach convincing conclusions for some subgroup analyses. Although this shortage was partially overcome by using an independent validation cohort of 240 colorectal cancer patients, future studies with larger sample sizes are still necessary to confirm our findings. Secondly, the current study was based on a single clinical center, while a future multicenter study with diverse ethnic composition is needed to test the generality of our results. Thirdly, although the broad-panel targeted sequencing that was used by this study covered the majority of cancer-relevant genes, it is still ideal to use whole genome sequencing to more comprehensively analyze the genomic profile if the budget is not a major concern.

Overall, our results elucidated the distinct genomic features in subgroup-specific colorectal cancer patients based on different stratification characteristics, including the timing of cancer onset, the microsatellite status, the disease stage, the metastatic status, and tumor anatomic locations. Our findings shed light on the molecular mechanism of colorectal cancer and could potentially facilitate the advancement of precise medication.

## Data availability statement

The data supporting this study's findings are deposited in the Genome Sequence Archive for Human (GSA-Human) repository, accession number HRA003355.

## Ethics statement

The studies involving human participants were reviewed and approved by Ethical Committee of Chinese PLA Central Hospital (Approval No. S2022-307-01). The patients/participants provided their written informed consent to participate in this study.

## Author contributions

PL and BJ contributed to study conception and design. PL, QM, YGX, and ZT conducted patient recruitment and data collection. HC, JZ, RY, QO, and XW conducted DNA sequencing and bioinformatics analysis. PL, BJ, and HC drafted the manuscript. HC, SW, YX, PL, QM, and YGX revised the manuscript.

## Funding

This work was supported by the National Natural Science Foundation of China [82173355].

## Acknowledgments

We would like to thank the patients and family members who gave their consent to present data in this study, as well as the investigators and research staff.

## Conflict of interest

HC, JZ, YX, SW, RY, QO, and XW are employees of Nanjing Geneseeq Technology Inc., China.

The remaining authors declare that the research was conducted in the absence of any commercial or financial relationships that could be construed as a potential conflict of interest.

## Publisher’s note

All claims expressed in this article are solely those of the authors and do not necessarily represent those of their affiliated organizations, or those of the publisher, the editors and the reviewers. Any product that may be evaluated in this article, or claim that may be made by its manufacturer, is not guaranteed or endorsed by the publisher.

## References

[1] Bhurgri H Samiullah S. Colon cancer screening - is it time yet? J Coll Physicians Surg Pak (2017) 27(6):327–8. 28689518

[2] Yang Y Han Z Li X Huang A Shi J Gu J. Epidemiology and risk factors of colorectal cancer in China. Chin J Cancer Res (2020) 32(6):729–41. doi: 10.21147/j.issn.1000-9604.2020.06.06 PMC7797231 33446996

[3] Siegel RL Miller KD Goding Sauer A Fedewa SA Butterly LF Anderson JC. Colorectal cancer statistics, 2020. CA Cancer J Clin (2020) 70(3):145–64. doi: 10.3322/caac.21601 32133645

[4] O'Sullivan DE Sutherland RL Town S Chow K Fan J Forbes N. Risk factors for early-onset colorectal cancer: A systematic review and meta-analysis. Clin Gastroenterol Hepatol (2022) 20(6):1229–1240.e5. doi: 10.1016/j.cgh.2021.01.037 33524598

[5] Toh JWT Phan K Reza F Chapuis P Spring KJ. Rate of dissemination and prognosis in early and advanced stage colorectal cancer based on microsatellite instability status: systematic review and meta-analysis. Int J Colorectal Dis (2021) 36(8):1573–96. doi: 10.1007/s00384-021-03874-1 33604737

[6] Dow LE O'Rourke KP Simon J Tschaharganeh DF van Es JH Clevers H. Apc restoration promotes cellular differentiation and reestablishes crypt homeostasis in colorectal cancer. Cell (2015) 161(7):1539–52. doi: 10.1016/j.cell.2015.05.033 PMC4475279 26091037

[7] Eklof V Wikberg ML Edin S Dahlin AM Jonsson BA Oberg A. The prognostic role of KRAS, BRAF, PIK3CA and PTEN in colorectal cancer. Br J Cancer (2013) 108(10):2153–63. doi: 10.1038/bjc.2013.212 PMC3670497 23660947

[8] Rechsteiner M von Teichman A Ruschoff JH Fankhauser N Pestalozzi B Schraml P. KRAS, BRAF, and TP53 deep sequencing for colorectal carcinoma patient diagnostics. J Mol Diagn (2013) 15(3):299–311. doi: 10.1016/j.jmoldx.2013.02.001 23531339

[9] Chen G Peng J Xiao Q Wu HX Wu X Wang F. Postoperative circulating tumor DNA as markers of recurrence risk in stages II to III colorectal cancer. J Hematol Oncol (2021) 14(1):80. doi: 10.1186/s13045-021-01089-z 34001194 PMC8130394

[10] Yang Z Yang N Ou Q Xiang Y Jiang T Wu X. Investigating novel resistance mechanisms to third-generation EGFR tyrosine kinase inhibitor osimertinib in non-small cell lung cancer patients. Clin Cancer Res (2018) 24(13):3097–107. doi: 10.1158/1078-0432.CCR-17-2310 29506987

[11] Tang WF Wu M Bao H Xu Y Lin JS Liang Y. Timing and origins of local and distant metastases in lung cancer. J Thorac Oncol (2021) 16(7):1136–48. doi: 10.1016/j.jtho.2021.02.023 33722707

[12] Li H Shan C Wu S Cheng B Fan C Cai L. Genomic profiling identified novel prognostic biomarkers in Chinese midline glioma patients. Front Oncol (2020) 10:607429. doi: 10.3389/fonc.2020.607429 33747896 PMC7968371

[13] Carter SL Cibulskis K Helman E McKenna A Shen H Zack T. Absolute quantification of somatic DNA alterations in human cancer. Nat Biotechnol (2012) 30(5):413–21. doi: 10.1038/nbt.2203 PMC4383288 22544022

[14] Newman AM Bratman SV Stehr H Lee LJ Liu CL Diehn M. FACTERA: A practical method for the discovery of genomic rearrangements at breakpoint resolution. Bioinformatics (2014) 30(23):3390–3. doi: 10.1093/bioinformatics/btu549 PMC4296148 25143292

[15] Alexandrov LB Nik-Zainal S Wedge DC Campbell PJ Stratton MR. Deciphering signatures of mutational processes operative in human cancer. Cell Rep (2013) 3(1):246–59. doi: 10.1016/j.celrep.2012.12.008 PMC3588146 23318258

[16] Rosenthal R McGranahan N Herrero J Taylor BS Swanton C. DeconstructSigs: Delineating mutational processes in single tumors distinguishes DNA repair deficiencies and patterns of carcinoma evolution. Genome Biol (2016) 17:31. doi: 10.1186/s13059-016-0893-4 26899170 PMC4762164

[17] Huang D Sun W Zhou Y Li P Chen F Chen H. Mutations of key driver genes in colorectal cancer progression and metastasis. Cancer Metastasis Rev (2018) 37(1):173–87. doi: 10.1007/s10555-017-9726-5 29322354

[18] Li W Qiu T Zhi W Shi S Zou S Ling Y. Colorectal carcinomas with KRAS codon 12 mutation are associated with more advanced tumor stages. BMC Cancer (2015) 15:340. doi: 10.1186/s12885-015-1345-3 25929517 PMC4423107

[19] Baran B Mert Ozupek N Yerli Tetik N Acar E Bekcioglu O Baskin Y. Difference between left-sided and right-sided colorectal cancer: A focused review of literature. Gastroenterol Res (2018) 11(4):264–73. doi: 10.14740/gr1062w PMC6089587 30116425

[20] Jiang Y Yan X Liu K Shi Y Wang C Hu J. Discovering the molecular differences between right- and left-sided colon cancer using machine learning methods. BMC Cancer (2020) 20(1):1012. doi: 10.1186/s12885-020-07507-8 33076847 PMC7574488

[21] Gonsalves WI Mahoney MR Sargent DJ Nelson GD Alberts SR Sinicrope FA. Patient and tumor characteristics and BRAF and KRAS mutations in colon cancer, NCCTG/Alliance N0147. J Natl Cancer Inst (2014) 106(7):dju106. doi: 10.1093/jnci/dju106 24925349 PMC4110470

[22] Sen M Wang X Hamdan FH Rapp J Eggert J Kosinsky RL. ARID1A facilitates KRAS signaling-regulated enhancer activity in an AP1-dependent manner in colorectal cancer cells. Clin Epigenet (2019) 11(1):92. doi: 10.1186/s13148-019-0690-5 PMC6585056 31217031

[23] Helming KC Wang X Wilson BG Vazquez F Haswell JR Manchester HE. ARID1B is a specific vulnerability in ARID1A-mutant cancers. Nat Med (2014) 20(3):251–4. doi: 10.1038/nm.3480 PMC3954704 24562383

[24] Feng A Li Y Li G Wang Y Wen Q Yang Z. Genomic features of organ-specific metastases in lung adenocarcinoma. Front Oncol (2022) 12:908759. doi: 10.3389/fonc.2022.908759 35912232 PMC9331737

[25] Samji P Rajendran MK Warrier VP Ganesh A Devarajan K. Regulation of hippo signaling pathway in cancer: A MicroRNA perspective. Cell Signal (2021) 78:109858. doi: 10.1016/j.cellsig.2020.109858 33253912

[26] Mohamed R Kennedy C Willmore WG. Responses of porcupine and wntless proteins to oxidative, hypoxic and endoplasmic reticulum stresses. Cell Signal (2021) 85:110047. doi: 10.1016/j.cellsig.2021.110047 34015469

[27] Wang X Sun D Tai J Chen S Yu M Ren D. TFAP2C promotes stemness and chemotherapeutic resistance in colorectal cancer via inactivating hippo signaling pathway. J Exp Clin Cancer Res (2018) 37(1):27. doi: 10.1186/s13046-018-0683-9 29439714 PMC5812206

[28] Langenbucher A Bowen D Sakhtemani R Bournique E Wise JF Zou L. An extended APOBEC3A mutation signature in cancer. Nat Commun (2021) 12(1):1602. doi: 10.1038/s41467-021-21891-0 33707442 PMC7952602

[29] Law EK Levin-Klein R Jarvis MC Kim H Argyris PP Carpenter MA. APOBEC3A catalyzes mutation and drives carcinogenesis in vivo. J Exp Med (2020) 217(12):e20200261. doi: 10.1084/jem.20200261 32870257 PMC7953736

[30] Venkatesan S Rosenthal R Kanu N McGranahan N Bartek J Quezada SA. Perspective: APOBEC mutagenesis in drug resistance and immune escape in HIV and cancer evolution. Ann Oncol (2018) 29(3):563–72. doi: 10.1093/annonc/mdy003 PMC5888943 29324969

[31] Liu Z Xiao J Wang N Ding J. LSD1 regulates the FOXF2-mediated wnt/beta-catenin signaling pathway by interacting with Ku80 to promote colon cancer progression. Am J Cancer Res (2022) 12(8):3693–712. PMC9442015 36119820

[32] Ramirez-Ramirez R Gutierrez-Angulo M Peregrina-Sandoval J Moreno-Ortiz JM Franco-Topete RA Cerda-Camacho FJ. Somatic deletion of KDM1A/LSD1 gene is associated to advanced colorectal cancer stages. J Clin Pathol (2020) 73(2):107–11. doi: 10.1136/jclinpath-2019-206128 PMC7027028 31471467

[33] Vendrell JA Thollet A Nguyen NT Ghayad SE Vinot S Bieche I. ZNF217 is a marker of poor prognosis in breast cancer that drives epithelial-mesenchymal transition and invasion. Cancer Res (2012) 72(14):3593–606. doi: 10.1158/0008-5472.CAN-11-3095 22593193

