# Supplementary material for: Comprehensive genomic profiling of colorectal cancer patients reveals differences in mutational landscapes among clinical and pathological subgroups
Source: Front Oncol. 2022 Nov 10;12:1000146. doi: 10.3389/fonc.2022.1000146 (PMC9685809; doi:10.3389/fonc.2022.1000146)
Supplement: Supplementary file 1 [file DataSheet_1.pdf]

Gene list of 425 gene panel	
**No.**	**GENE**
1	ABCB1 (MDR1)
2	ABCC2 (MRP2)
3	ADGRB3 (BAI3)
4	ADH1B
5	AFDN (MLLT4)
6	AIP
7	AKT1
8	AKT2
9	AKT3
10	ALDH2
11	ALK
12	AMER1 (FAM123B)
13	APC
14	AR
15	ARAF
16	ARID1A
17	ARID1B
18	ARID2
19	ARID5B
20	ASCL4
21	ASXL1
22	ATF1
23	ATIC
24	ATM
25	ATR
26	ATRX
27	AURKA
28	AURKB
29	AXIN2
30	AXL
31	B2M
32	BAD
33	BAK1
34	BAP1
35	BARD1
36	BAX
37	BCL2
38	BCL2L11 (BIM)

39	BCR
40	BIRC3
41	BLM
42	BMPR1AA62
43	BRAF
44	BRCA1
45	BRCA2
46	BRD4
47	BRIP1
48	BTG2
49	BTK
50	BUB1B
51	CASP8
52	CBL
53	CBLB
54	CCN6 (WISP3)
55	CCND1
56	CCNE1
57	CD274 (PD-L1)
58	CD74
59	CDA
60	CDC73
61	CDH1
62	CDK10
63	CDK12
64	CDK4
65	CDK6
66	CDK8
67	CDKN1A
68	CDKN1B
69	CDKN1C
70	CDKN2A
71	CDKN2B
72	CDKN2C
73	CEBPA
74	CEP57
75	CHD4
76	CHD8
77	CHEK1
78	CHEK2
79	CREBBP

80	CRKL
81	CSF1R
82	CTCF
83	CTLA4
84	CTNNB1
85	CUL3
86	CUX1
87	CXCR4
88	CYLD
89	CYP19A1
90	CYP2A13
91	CYP2A6
92	CYP2A7
93	CYP2B6*6
94	CYP2C19*2
95	CYP2C9*3
96	CYP2D6
97	CYP3A4*4
98	CYP3A5
99	CYSLTR2
100	DAXX
101	DDR2
102	DENND1A
103	DHFR
104	DICER1
105	DLL3
106	DNMT3A
107	DOT1L
108	DPYD
109	DTL (CDT2)
110	DUSP2
111	EGFR
112	EIF1AX
113	EMSY (c11orf30)
114	EP300

115	EPAS1
116	EPCAM
117	EPHA2
118	EPHA3
119	EPHA5
120	ERBB2 (HER2)
121	ERBB3
122	ERBB4
123	ERBIN (ERBB2IP)
124	ERCC1
125	ERCC2
126	ERCC3
127	ERCC4
128	ERCC5
129	ESR1
130	ETV1
131	ETV4
132	ETV5
133	ETV6
134	EWSR1
135	EXT1
136	EXT2
137	EZH2
138	EZR
139	FANCA
140	FANCC
141	FANCD2
142	FANCE
143	FANCF
144	FANCG
145	FANCI
146	FANCL
147	FANCM
148	FAT1
149	FBXW7
150	FGF19
151	FGFR1
152	FGFR2
153	FGFR3
154	FGFR4
155	FH

156	FLCN
157	FLT1 (VEGFR1)
158	FLT3
159	FLT4
160	FOXA1
161	FOXL2
162	FOXP1
163	FRG1
164	GATA1
165	GATA2
166	GATA3
167	GATA4
168	GATA6
169	GNA11
170	GNAQ
171	GNAS
172	GRIN2A
173	GRM3
174	GRM8
175	GSTM1
176	GSTM4
177	GSTP1
178	GSTT1
179	HDAC2
180	HDAC9
181	HGF
182	HLA-A
183	HNF1A
184	HNF1B
185	HRAS
186	IDH1
187	IDH2
188	IFNA6
189	IFNB1
190	IFNE
191	IFNG
192	IFNGR1
193	IFNGR2
194	IGF1R
195	IGF2
196	IKBKE
197	IKZF1

198	IL7R
199	INPP4B
200	IRF2
201	JAK1
202	JAK2
203	JAK3
204	JARID2
205	JUN
206	KDM5A
207	KDR (VEGFR2)
208	KEAP1
209	KIF1B
210	KIT
211	KITLG
212	KLLN
213	KMT2A (MLL)
214	KMT2B
215	KMT2C
216	KMT2D (MLL2)
217	KRAS
218	LHCGR
219	LMO1
220	LRP1B
221	LYN
222	LZTR1
223	MAP2K1 (MEK1)
224	MAP2K2 (MEK2)
225	MAP2K4
226	MAP3K1
227	MAP3K4
228	MAX
229	MCL1
230	MDM2
231	MDM4
232	MECOM
233	MED12
234	MEF2B
235	MEN1
236	MET

237	MGMT
238	MITF
239	MLH1
240	MLH3
241	MLLT1
242	MLLT3
243	MPL
244	MRE11 (MRE11A)
245	MSH2
246	MSH6
247	MTHFR
248	MTOR
249	MUTYH
250	MYC
251	MYCL (MYCL1)
252	MYCN
253	MYD88
254	MYH9
255	NAT1
256	NBN
257	NCOR1
258	NF1
259	NF2
260	NFE2L2
261	NFKBIA
262	NKX2-1
263	NOTCH1
264	NOTCH2
265	NOTCH3
266	NPM1
267	NQO1
268	NRAS
269	NRG1
270	NSD1
271	NTRK1
272	NTRK2
273	NTRK3
274	NUTM1
275	PAK3
276	PALB2
277	PALLD
278	PARP1

279	PARP2
280	PAX5
281	PBRM1
282	PDCD1 (PD1)
283	PDCD1LG2 (PD-L2)
284	PDE11A
285	PDGFRA
286	PDGFRB
287	PDK1
288	PGR
289	PHOX2B
290	PIK3C3
291	PIK3CA
292	PIK3CD
293	PIK3R1
294	PIK3R2
295	PKHD1
296	PLAG1
297	PLCB4
298	PLK1
299	PMS1
300	PMS2
301	POLD1
302	POLD3
303	POLE
304	POLH
305	POT1
306	PPARD
307	PPP2R1A
308	PRDM1
309	PREX2
310	PRF1
311	PRKACA
312	PRKAR1A
313	PRKCI
314	PRKDC
315	PRKN (PARK2)
316	PRSS1
317	PRSS3
318	PTCH1

319	PTEN
320	PTK2
321	PTPN11
322	PTPN13
323	QKI
324	RAC1
325	RAC3
326	RAD50
327	RAD51
328	RAD51B
329	RAD51C
330	RAD51D
331	RAD54L
332	RAF1
333	RARA
334	RARG
335	RASGEF1A
336	RB1
337	RECQL4
338	RELN
339	RET
340	RHOA
341	RICTOR
342	RNF43
343	ROS1
344	RPTOR
345	RRM1
346	RUNX1
347	RUNX1T1
348	SBDS
349	SDC4
350	SDHA
351	SDHB
352	SDHC
353	SDHD
354	SEPTIN9 (SEPT9)
355	SETBP1
356	SETD2
357	SF3B1
358	SGK1
359	SKP2

360	SLC34A2
361	SLC3A2
362	SMAD2
363	SMAD3
364	SMAD4
365	SMAD7
366	SMARCA4
367	SMARCB1
368	SMO
369	SOCS1
370	SOS1
371	SOX2
372	SPOP
373	SPRED1
374	SPRY4
375	SRC
376	SRSF2
377	SRY
378	STAG2
379	STAT3
380	STK11
381	STMN1
382	SUFU
383	TACC3
384	TAP1
385	TAP2
386	TEK
387	TEKT4
388	TERC
389	TERT
390	TET2
391	TGFBR2
392	THADA
393	TMEM127
394	TMPRSS2
395	TNFAIP3
396	TNFRSF11A
397	TNFRSF14
398	TNFRSF19

399	TNFSF11
400	TOP1
401	TOP2A
402	TP53
403	TP63
404	TPMT
405	TSC1
406	TSC2
407	TSHR
408	TTF1
409	TUBB3
410	TYMS
411	U2AF1
412	UGT1A1
413	VAMP2
414	VEGFA
415	VHL
416	WAS
417	WRN
418	WT1
419	XPA
420	XPC
421	XRCC1
422	XRCC2
423	YAP1
424	ZNF217
425	ZNF703
