# Supplementary material for: Comprehensive genomic profiling of colorectal cancer patients reveals differences in mutational landscapes among clinical and pathological subgroups
Source: Front Oncol. 2022 Nov 10;12:1000146. doi: 10.3389/fonc.2022.1000146 (PMC9685809; doi:10.3389/fonc.2022.1000146)
Supplement: Supplementary file 2 [file DataSheet_2.pdf]

Table S2. The mutation list of tumor samples

#Patient_No=Patient Number
#Gene=HGNC Gene Symbol
#Chr_start=Start position on the Chromosome. Chromosome:Start
#Chr_end=End position on the Chromosome. Chromosome:End
#Ref=Reference Allele
#Alt=Altered Allele
#Variant_Classification=Classification of variants
#AAChange=Change of Amino Acid
#AF=Proportion of Altered Allele

**Patient_No**	**Gene**	**AAChange**	**Variant Classificatio**
P1	ARID1A	c.5329G>T(p.E1777*)	STOP GAINED
P1	PDGFRB		Amplication
P1	KMT2B	c.4738C>A(p.Q1580K)	MISSENSE
P1	APC	c.646C>T(p.R216*)	STOP GAINED
P1	FANCD2	c.3139G>T(p.G1047*)	STOP GAINED
P1	EXT2	c.1084T>G(p.S362A)	MISSENSE
P1	ERCC4	c.1177G>A(p.A393T)	MISSENSE
P1	CSF1R		Amplication
P1	MTOR	c.5930C>A(p.T1977K)	MISSENSE
P1	APC	c.8192C>A(p.P2731H)	MISSENSE
P1	RB1	c.2206C>A(p.Q736K)	MISSENSE
P1	JAK1	c.2593G>T(p.V865L)	MISSENSE
P1	KRAS	c.34G>T(p.G12C)	MISSENSE
P1	APC	c.734C>A(p.S245*)	STOP GAINED
P1	ERBB4	c.2685G>T(p.R895S)	MISSENSE
P1	FBXW7	c.608C>A(p.S203*)	STOP GAINED
P1	TP53	c.725G>A(p.C242Y)	MISSENSE
P1	CSF1R		Amplication
P1	PDGFRB		Amplication
P1	CEBPA		Deletion
P2	FGFR1		Amplication
P2	FGFR1	FGFR1:exon2~IGR (upst	FUSION
P2	APC	c.3944C>A(p.S1315*)	STOP GAINED
P2	LRP1B	c.2504-1G>C	SPLICE
P2	MET	c.1447T>A(p.S483T)	MISSENSE
P2	AXIN2	c.330C>A(p.F110L)	MISSENSE
P2	POLH	c.2083C>T(p.R695C)	MISSENSE
P2	KLLN	c.428_433delCCCGCC(p	INFRAME INDEL
P2	FGFR1		Amplication
P2	FGFR1		Amplication
P2	FGFR1		Amplication
P2	FGFR1		Amplication
P3	MAP2K4	c.439A>G(p.M147V)	MISSENSE
P3	PKHD1	c.5684A>G(p.E1895G)	MISSENSE
P3	BRAF	c.1799T>A(p.V600E)	MISSENSE
P3	SMAD4	c.250-1G>C	SPLICE

P3	TP53	c.524G>A(p.R175H)	MISSENSE
P3	PTEN		Deletion
P3	CDKN2B		Deletion
P4	FGFR2	c.1393G>A(p.E465K)	MISSENSE
P4	PRKCI	c.396G>T(p.W132C)	MISSENSE
P4	CHD4	c.3748G>A(p.D1250N)	MISSENSE
P4	KEAP1	c.701G>C(p.R234P)	MISSENSE
P4	CDH1	c.2216A>T(p.E739V)	MISSENSE
P5	APC	c.3934G>T(p.G1312*)	STOP GAINED
P5	PIK3R1	c.41A>T(p.Y14F)	MISSENSE
P5	FANCD2	c.2041A>G(p.K681E)	MISSENSE
P5	SMAD4	c.1087T>G(p.C363G)	MISSENSE
P5	EP300	c.1762G>A(p.V588I)	MISSENSE
P5	TP53	c.824G>A(p.C275Y)	MISSENSE
P5	KRAS	c.38G>A(p.G13D)	MISSENSE
P5	CDK6	c.7_9delAAG(p.K3del)	INFRAME INDEL
P5	PIK3R1	c.40dupT(p.Y14Lfs*2)	FRAMESHIFT
P5	ERBB4	c.305delT(p.L102Yfs*2	FRAMESHIFT
P6	TMPRSS2	c.145G>A(p.V49M)	MISSENSE
P6	NSD1	c.404C>T(p.S135F)	MISSENSE
P6	TP53	c.743G>A(p.R248Q)	MISSENSE
P6	SMAD4	c.1484T>C(p.L495P)	MISSENSE
P6	ATM	c.7751_7754delCTAA(p	FRAMESHIFT
P6	APC	c.4395delT(p.S1465Rfs	FRAMESHIFT
P7	SLC3A2	c.899delA(p.K300Rfs*3	FRAMESHIFT
P7	AMER1	c.2074G>A(p.E692K)	MISSENSE
P7	AMER1	c.1921C>T(p.R641*)	STOP GAINED
P7	SMARCB1	c.946C>T(p.R316W)	MISSENSE
P7	SMARCA4	c.442G>A(p.G148R)	MISSENSE
P7	RECQL4	c.3349G>A(p.G1117R)	MISSENSE
P7	KMT2A	c.2318delC(p.P773Rfs*	FRAMESHIFT
P7	NSD1	c.2175_2176invGT(p.S	MISSENSE
P7	CYLD	c.64delT(p.Y22Tfs*25)	FRAMESHIFT
P7	RNF43	c.1976delG(p.G659Vfs*	FRAMESHIFT
P7	TGFBR2	c.317T>C(p.L106P)	MISSENSE
P7	LZTR1	c.27delG(p.Q10Rfs*15)	FRAMESHIFT
P7	MAP2K4	c.961G>A(p.V321M)	MISSENSE
P7	EP300	c.1943G>A(p.R648Q)	MISSENSE
P7	TGFBR2	c.382_383delAA(p.K12	FRAMESHIFT
P7	BRAF	c.1682C>T(p.A561V)	MISSENSE
P7	TTF1	c.2671G>A(p.A891T)	MISSENSE
P7	RPTOR	c.2362C>T(p.R788C)	MISSENSE
P7	BRCA2	c.9097delA(p.T3033Lfs	FRAMESHIFT
P7	JARID2	c.1186dupG(p.A396Gfs	FRAMESHIFT
P7	SRC	c.659G>A(p.R220H)	MISSENSE
P7	B2M	c.45_48delTTCT(p.S16A	FRAMESHIFT
P7	CDKN2A	c.335G>A(p.R112H)	MISSENSE

P7	TERT	c.1931C>T(p.T644M)	MISSENSE
P7	FOXP1	c.1328A>G(p.Y443C)	MISSENSE
P7	TGFBR2	c.321dupC(p.Y108Lfs*3	FRAMESHIFT
P7	PRKCI	c.30G>A(p.M10I)	MISSENSE
P7	PTCH1	c.2082G>T(p.Q694H)	MISSENSE
P7	CASP8	c.793C>T(p.R265W)	MISSENSE
P7	APC	c.6747dupA(p.G2250Rf	FRAMESHIFT
P7	CHD4	c.2923C>T(p.R975C)	MISSENSE
P7	RRM1	c.902T>C(p.L301P)	MISSENSE
P7	NRG1	c.1361delC(p.P454Lfs*1	FRAMESHIFT
P7	NSD1	c.1034C>T(p.P345L)	MISSENSE
P7	PARK2	c.572G>A(p.R191Q)	MISSENSE
P7	ARID1B	c.4556G>A(p.R1519H)	MISSENSE
P7	NAT1	c.98G>A(p.R33Q)	MISSENSE
P7	EP300	c.5817G>T(p.Q1939H)	MISSENSE
P7	DOT1L	c.3097G>A(p.G1033S)	MISSENSE
P7	RNF43	c.375+2T>C	SPLICE
P7	TSC1	c.1838C>A(p.P613Q)	MISSENSE
P7	BAX	c.130G>A(p.E44K)	MISSENSE
P7	CDK12	c.893G>A(p.R298Q)	MISSENSE
P7	RAD54L	c.433G>A(p.V145I)	MISSENSE
P7	FLT4	c.1924delC(p.R642Afs*	FRAMESHIFT
P7	KDR	c.3773C>T(p.T1258M)	MISSENSE
P7	CREBBP	c.3668G>A(p.R1223H)	MISSENSE
P7	TGFBR2	c.1198C>T(p.L400F)	MISSENSE
P7	BAP1	c.175C>T(p.R59W)	MISSENSE
P7	FBXW7	c.1393C>T(p.R465C)	MISSENSE
P7	B2M	c.43_44delCT(p.L15Ffs*	FRAMESHIFT
P7	POT1	c.1892A>G(p.E631G)	MISSENSE
P7	ASXL1	c.1934dupG(p.G646Wf	FRAMESHIFT
P7	PRDM1	c.126G>T(p.E42D)	MISSENSE
P7	RAD50	c.1722dupA(p.Q575Tfs	FRAMESHIFT
P7	ATM	c.9023G>A(p.R3008H)	MISSENSE
P7	MSH2	c.1288A>T(p.K430*)	STOP GAINED
P7	NBN	c.797C>T(p.P266L)	MISSENSE
P7	KMT2B	c.3057delA(p.G1020Afs	FRAMESHIFT
P7	BAX	c.34_34+1insT(p.G12Vf	FRAMESHIFT
P7	GRIN2A	c.2147C>T(p.A716V)	MISSENSE
P7	SMARCA4	c.2729C>T(p.T910M)	MISSENSE
P7	DOT1L	c.3694G>A(p.A1232T)	MISSENSE
P7	RNF43	c.349_350delCGinsA(p.	FRAMESHIFT
P7	POLD1	c.2158G>A(p.V720I)	MISSENSE
P7	BRD4	c.3178C>T(p.R1060C)	MISSENSE
P7	PLCB4	c.685dupA(p.I229Nfs*7	FRAMESHIFT
P7	PIK3C3	c.1459T>C(p.S487P)	MISSENSE
P7	QKI	c.401delA(p.K134Rfs*1	FRAMESHIFT
P7	MSH6		Deletion

P8	NF1	c.2281G>A(p.A761T)	MISSENSE
P8	AURKA	c.481C>T(p.L161F)	MISSENSE
P8	GRIN2A	c.3475C>T(p.R1159C)	MISSENSE
P8	RAF1	c.770C>T(p.S257L)	MISSENSE
P8	KMT2C	c.2690G>A(p.R897Q)	MISSENSE
P8	ROS1	c.3025G>A(p.E1009K)	MISSENSE
P8	NFE2L2	c.938T>G(p.L313R)	MISSENSE
P8	CDK12	c.923G>T(p.R308I)	MISSENSE
P8	POLE	c.857C>G(p.P286R)	MISSENSE
P8	APC	c.1307A>C(p.N436T)	MISSENSE
P8	GATA1	c.1117T>C(p.S373P)	MISSENSE
P8	GRIN2A	c.581G>A(p.S194N)	MISSENSE
P8	RHOA	c.365G>A(p.R122Q)	MISSENSE
P8	APC	c.694C>T(p.R232*)	STOP GAINED
P8	SMARCA4	c.2644G>A(p.E882K)	MISSENSE
P8	BARD1	c.775G>T(p.D259Y)	MISSENSE
P8	RET	c.2083C>T(p.P695S)	MISSENSE
P8	ROS1	c.367A>C(p.N123H)	MISSENSE
P8	C11orf30	c.2648C>T(p.S883L)	MISSENSE
P8	FLT1	c.2926A>C(p.S976R)	MISSENSE
P8	ERCC5	c.628G>T(p.E210*)	STOP GAINED
P8	ATR	c.5942A>C(p.E1981A)	MISSENSE
P8	PDGFRA	c.649G>T(p.E217*)	STOP GAINED
P8	FANCC	c.956C>T(p.T319M)	MISSENSE
P8	TOP2A	c.2719T>G(p.Y907D)	MISSENSE
P8	ERCC5	c.211C>T(p.R71C)	MISSENSE
P8	BRCA2	c.5782G>T(p.E1928*)	STOP GAINED
P8	NBN	c.904C>T(p.L302F)	MISSENSE
P8	CYSLTR2	c.626G>A(p.G209D)	MISSENSE
P8	NOTCH1	c.6376G>A(p.G2126R)	MISSENSE
P8	BRD4	c.297G>T(p.K99N)	MISSENSE
P8	TAP2	c.1795G>T(p.D599Y)	MISSENSE
P8	CBLB	c.2850G>T(p.K950N)	MISSENSE
P8	TAP2	c.1620C>A(p.C540*)	STOP GAINED
P8	PMS2	c.1862C>A(p.S621Y)	MISSENSE
P8	KMT2A	c.9262C>T(p.L3088F)	MISSENSE
P8	ROS1	c.3489G>T(p.K1163N)	MISSENSE
P8	PIK3R1	c.1042C>T(p.R348*)	STOP GAINED
P8	FLCN	c.50G>A(p.R17H)	MISSENSE
P8	TET2	c.3310T>G(p.F1104V)	MISSENSE
P8	PGR	c.1817G>T(p.R606I)	MISSENSE
P8	THADA	c.5822C>T(p.S1941L)	MISSENSE
P8	SETD2	c.1334G>A(p.R445H)	MISSENSE
P8	ARID1A	c.3419C>A(p.S1140Y)	MISSENSE
P8	ABCB1	c.836A>C(p.K279T)	MISSENSE
P8	ATM	c.5584C>T(p.L1862F)	MISSENSE
P8	CHD4	c.3659A>C(p.K1220T)	MISSENSE

P8	BRCA2	c.7559G>A(p.R2520Q)	MISSENSE
P8	PKHD1	c.6084G>T(p.K2028N)	MISSENSE
P8	HGF	c.1915A>C(p.N639H)	MISSENSE
P8	PDGFRB	c.2335G>A(p.V779I)	MISSENSE
P8	CYP2D6	c.262C>T(p.R88C)	MISSENSE
P8	FH	c.472_473delAGinsCA(p	MISSENSE
P8	ROS1	c.3250A>C(p.I1084L)	MISSENSE
P8	NTRK2	c.1150A>C(p.I384L)	MISSENSE
P8	RET	c.1187C>T(p.S396L)	MISSENSE
P8	APC	c.5522A>T(p.D1841V)	MISSENSE
P8	KITLG	c.425G>T(p.R142I)	MISSENSE
P8	PIK3CA	c.2236G>A(p.D746N)	MISSENSE
P8	RPTOR	c.333G>T(p.E111D)	MISSENSE
P8	ATR	c.760G>T(p.E254*)	STOP GAINED
P8	SF3B1	c.756A>C(p.K252N)	MISSENSE
P8	ALK	c.3541C>T(p.R1181C)	MISSENSE
P8	EPAS1	c.2216G>A(p.R739Q)	MISSENSE
P8	ERBB4	c.2557T>G(p.S853A)	MISSENSE
P8	TAP2	c.1051G>T(p.E351*)	STOP GAINED
P8	AR	c.878C>A(p.S293Y)	MISSENSE
P8	FAT1	c.2542G>T(p.D848Y)	MISSENSE
P8	FGFR2	c.2387C>A(p.S796Y)	MISSENSE
P8	MLH3	c.1666G>T(p.D556Y)	MISSENSE
P8	ERBB4	c.458A>C(p.K153T)	MISSENSE
P8	CBLB	c.2078C>A(p.S693Y)	MISSENSE
P8	ROS1	c.980G>T(p.G327V)	MISSENSE
P8	ROS1	c.1579G>T(p.E527*)	STOP GAINED
P8	TAP2	c.405A>C(p.K135N)	MISSENSE
P8	SETD2	c.3700G>T(p.E1234*)	STOP GAINED
P8	BARD1	c.1435T>G(p.L479V)	MISSENSE
P8	FANCD2	c.821G>T(p.R274I)	MISSENSE
P8	SETD2	c.4568G>A(p.R1523H)	MISSENSE
P8	IFNG	c.355A>T(p.T119S)	MISSENSE
P8	CDA	c.429G>T(p.Q143H)	MISSENSE
P8	TGFBR2	c.1208G>A(p.R403H)	MISSENSE
P8	LYN	c.998G>A(p.S333N)	MISSENSE
P8	MAP2K4	c.395G>T(p.R132I)	MISSENSE
P8	LRP1B	c.466C>A(p.Q156K)	MISSENSE
P8	PREX2	c.3898A>G(p.T1300A)	MISSENSE
P8	GRIN2A	c.148G>A(p.E50K)	MISSENSE
P8	FANCC	c.1603C>T(p.R535C)	MISSENSE
P8	SETD2	c.2794G>A(p.V932I)	MISSENSE
P8	WRN	c.1519G>T(p.E507*)	STOP GAINED
P8	MAP3K1	c.4165T>G(p.F1389V)	MISSENSE
P8	ARID2	c.1258G>A(p.E420K)	MISSENSE
P8	APC	c.503G>T(p.R168I)	MISSENSE
P8	WAS	c.93G>T(p.E31D)	MISSENSE

P8	XPC	c.1536A>C(p.K512N)	MISSENSE
P8	BRCA2	c.2544G>T(p.K848N)	MISSENSE
P8	CTNNB1	c.664T>C(p.S222P)	MISSENSE
P8	CASP8	c.52G>T(p.D18Y)	MISSENSE
P8	ATM	c.8071C>T(p.R2691C)	MISSENSE
P8	AKT1	c.1003C>A(p.L335M)	MISSENSE
P8	ATM	c.6013C>A(p.L2005I)	MISSENSE
P8	IL7R	c.1151C>A(p.S384Y)	MISSENSE
P8	SETD2	c.2751T>G(p.S917R)	MISSENSE
P8	FAT1	c.3575A>C(p.K1192T)	MISSENSE
P8	FANCC	c.263A>C(p.K88T)	MISSENSE
P8	MPL	c.373C>A(p.L125I)	MISSENSE
P8	EP300	c.4219A>C(p.K1407Q)	MISSENSE
P8	LRP1B	c.1598G>T(p.G533V)	MISSENSE
P8	PTPN11	c.923A>C(p.N308T)	MISSENSE
P8	MRE11A	c.391G>A(p.D131N)	MISSENSE
P8	ZNF217	c.1731G>T(p.K577N)	MISSENSE
P8	PRKCI	c.114A>G(p.I38M)	MISSENSE
P8	BRCA2	c.2275C>A(p.L759I)	MISSENSE
P8	TEK	c.3022G>A(p.E1008K)	MISSENSE
P8	ETV6	c.849C>A(p.H283Q)	MISSENSE
P8	DOT1L	c.4064C>T(p.S1355L)	MISSENSE
P8	XRCC2	c.137T>G(p.F46C)	MISSENSE
P8	TAP2	c.223dupC(p.L75Pfs*92	FRAMESHIFT
P8	APC	c.4348C>T(p.R1450*)	STOP GAINED
P8	BRCA2	c.4301A>C(p.K1434T)	MISSENSE
P8	NSD1	c.2378_2379insC(p.K79	FRAMESHIFT
P8	BAI3	c.544G>T(p.E182*)	STOP GAINED
P8	BLM	c.79C>A(p.L27I)	MISSENSE
P8	FANCI	c.1315G>T(p.E439*)	STOP GAINED
P8	PTK2	c.570G>T(p.K190N)	MISSENSE
P8	PLK1	c.912C>A(p.F304L)	MISSENSE
P8	PDE11A	c.1112C>T(p.S371F)	MISSENSE
P8	LRP1B	c.10912C>T(p.R3638W)	MISSENSE
P8	CYLD	c.372A>C(p.Q124H)	MISSENSE
P8	PDGFRA	c.2041G>T(p.D681Y)	MISSENSE
P8	MAP2K4	c.200G>T(p.G67V)	MISSENSE
P8	ZNF217	c.3089G>T(p.R1030I)	MISSENSE
P8	CYSLTR2	c.582A>C(p.K194N)	MISSENSE
P8	MAP3K4	c.665G>A(p.R222H)	MISSENSE
P8	LRP1B	c.6018G>T(p.E2006D)	MISSENSE
P8	JAK1	c.3416G>A(p.R1139Q)	MISSENSE
P8	MTOR	c.7513C>T(p.R2505*)	STOP GAINED
P8	CYSLTR2	c.703G>T(p.E235*)	STOP GAINED
P8	PIK3R1	c.1157G>A(p.R386Q)	MISSENSE
P8	TUBB3	c.891G>T(p.K297N)	MISSENSE
P8	SETD2	c.71+2T>C	SPLICE

P8	SF3B1	c.944G>A(p.R315Q)	MISSENSE
P8	CHEK2	c.1369G>T(p.E457*)	STOP GAINED
P8	ROS1	c.2293A>C(p.K765Q)	MISSENSE
P8	ESR1	c.1702T>G(p.L568V)	MISSENSE
P8	ATRX	c.3171G>T(p.K1057N)	MISSENSE
P8	NKX2-1	c.821C>T(p.A274V)	MISSENSE
P8	PREX2	c.2894A>C(p.D965A)	MISSENSE
P8	MLLT4	c.131G>T(p.R44I)	MISSENSE
P8	TAP1	c.806G>A(p.G269D)	MISSENSE
P8	PDGFRA	c.2166T>G(p.I722M)	MISSENSE
P8	NFE2L2	c.1772T>C(p.V591A)	MISSENSE
P8	PTPN13	c.2767C>T(p.R923*)	STOP GAINED
P8	POT1	c.587T>G(p.V196G)	MISSENSE
P8	PDE11A	c.1865C>T(p.A622V)	MISSENSE
P8	IKBKE	c.419A>G(p.N140S)	MISSENSE
P8	HSPH1	c.1180T>G(p.F394V)	MISSENSE
P8	JAK2	c.1244A>C(p.K415T)	MISSENSE
P8	MSH2	c.1738G>T(p.E580*)	STOP GAINED
P8	ATRX	c.1911G>T(p.E637D)	MISSENSE
P8	QKI	c.451T>G(p.L151V)	MISSENSE
P8	CTNNB1	c.264G>T(p.M88I)	MISSENSE
P8	FOXP1	c.1573C>T(p.R525*)	STOP GAINED
P8	WAS	c.122G>T(p.R41L)	MISSENSE
P8	DTL	c.544G>A(p.V182M)	MISSENSE
P8	KMT2B	c.3704G>A(p.R1235Q)	MISSENSE
P8	PRKAR1A	c.153G>T(p.R51S)	MISSENSE
P8	ATRX	c.3021A>C(p.Q1007H)	MISSENSE
P8	ATRX	c.3392G>T(p.R1131I)	MISSENSE
P8	PGR	c.1349C>A(p.S450Y)	MISSENSE
P8	APC	c.4154G>T(p.S1385I)	MISSENSE
P8	CDC73	c.1450C>T(p.R484C)	MISSENSE
P8	ATM	c.6001T>G(p.L2001V)	MISSENSE
P8	WAS	c.280C>T(p.R94W)	MISSENSE
P8	NF1	c.7720G>T(p.E2574*)	STOP GAINED
P8	ATIC	c.1136G>A(p.R379Q)	MISSENSE
P8	IFNE	c.95G>T(p.R32I)	MISSENSE
P8	PDGFRA	c.1425G>T(p.E475D)	MISSENSE
P8	AMER1	c.2570G>A(p.R857Q)	MISSENSE
P8	BRIP1	c.752G>A(p.R251H)	MISSENSE
P8	NBN	c.1589C>A(p.S530Y)	MISSENSE
P8	FAT1	c.3209G>A(p.R1070Q)	MISSENSE
P8	NF2	c.300T>G(p.F100L)	MISSENSE
P8	MUTYH	c.1517T>C(p.M506T)	MISSENSE
P8	PKHD1	c.6388G>A(p.A2130T)	MISSENSE
P8	CHEK2	c.1519G>T(p.A507S)	MISSENSE

P8	AXL	c.2425G>A(p.E809K)	MISSENSE
P8	AR	c.1472C>T(p.A491V)	MISSENSE
P8	ABCB1	c.2246G>A(p.R749Q)	MISSENSE
P8	ERCC5	c.2502T>G(p.Y834*)	STOP GAINED
P8	GRM8	c.1502A>G(p.D501G)	MISSENSE
P8	CTNNB1	c.269G>A(p.R90Q)	MISSENSE
P8	BTK	c.1469G>A(p.R490H)	MISSENSE
P8	FANCD2	c.1092G>A(p.W364*)	STOP GAINED
P8	ERBB4	c.2584T>G(p.F862V)	MISSENSE
P8	NTRK1	c.1237G>A(p.E413K)	MISSENSE
P8	CYP2A6	c.821G>A(p.R274H)	MISSENSE
P8	SMAD2	c.448G>A(p.E150K)	MISSENSE
P8	TPMT	c.487C>T(p.R163C)	MISSENSE
P8	ERBB2IP	c.2455G>A(p.V819I)	MISSENSE
P8	RAD50	c.3880G>A(p.D1294N)	MISSENSE
P8	ATM	c.3980T>G(p.L1327*)	STOP GAINED
P8	RAD51C	c.550G>A(p.A184T)	MISSENSE
P8	FGFR3	c.148G>A(p.V50I)	MISSENSE
P8	FGFR1	c.2461C>T(p.R821C)	MISSENSE
P8	RAF1	c.200G>T(p.R67I)	MISSENSE
P8	PIK3CA	c.3044C>A(p.S1015Y)	MISSENSE
P8	BMPR1A	c.531-1G>T	SPLICE
P8	MSH2	c.1289A>T(p.K430I)	MISSENSE
P8	LRP1B	c.9912C>A(p.F3304L)	MISSENSE
P8	FAT1	c.11180G>T(p.G3727V)	MISSENSE
P8	ATR	c.2770A>C(p.S924R)	MISSENSE
P8	MTOR	c.4348T>G(p.Y1450D)	MISSENSE
P8	BRCA1	c.1024C>A(p.L342M)	MISSENSE
P8	MAP3K1	c.2759T>G(p.L920W)	MISSENSE
P8	TGFBR2	c.1234G>T(p.D412Y)	MISSENSE
P8	DPYD	c.964T>C(p.C322R)	MISSENSE
P8	PKHD1	c.527G>T(p.S176I)	MISSENSE
P8	KMT2B	c.7807G>T(p.E2603*)	STOP GAINED
P8	CRKL	c.737G>A(p.R246K)	MISSENSE
P9	FGFR4	c.787G>T(p.V263L)	MISSENSE
P9	ESR1	c.988G>A(p.E330K)	MISSENSE
P9	FBXW7	c.7C>T(p.Q3*)	STOP GAINED
P9	LRP1B	c.2095G>A(p.G699S)	MISSENSE
P10	TUBB3	c.1141A>T(p.I381F)	MISSENSE
P10	IGF1R	c.722C>T(p.A241V)	MISSENSE
P10	ERCC4	c.1444G>C(p.E482Q)	MISSENSE
P10	KMT2B	c.6661C>T(p.P2221S)	MISSENSE
P10	PDGFRA	c.248C>T(p.T83M)	MISSENSE
P10	AXL	c.1642C>G(p.Q548E)	MISSENSE
P10	TP53	c.499C>T(p.Q167*)	STOP GAINED
P10	ABCB1	c.1350+2T>A	SPLICE

P10	FANCF	c.187C>T(p.H63Y)	MISSENSE
P10	BRAF	c.1799T>A(p.V600E)	MISSENSE
P10	IDH1	c.719A>G(p.E240G)	MISSENSE
P10	POLD1	c.829C>G(p.L277V)	MISSENSE
P10	FAT1	c.12497_13138+1533de	LARGE FRAGMENT D
P10	TUBB3		Amplication
P10	PKHD1	c.1991G>A(p.W664*)	STOP GAINED
P10	DENND1A	c.1426G>C(p.V476L)	MISSENSE
P10	GRIN2A	c.237delC(p.K80Rfs*30)	FRAMESHIFT
P10	EXT1	c.652_962+12delinsCCA	SPLICE
P10	CDKN1B		Deletion
P10	FLT3		Amplication
P10	PTPRD		Deletion
P10	MEN1		Deletion
P11	PRKCI	c.1438C>T(p.R480C)	MISSENSE
P11	APC	c.1495C>T(p.R499*)	STOP GAINED
P11	SMO	c.430C>T(p.R144C)	MISSENSE
P11	NQO1	c.359T>A(p.V120E)	MISSENSE
P11	KRAS	c.35G>A(p.G12D)	MISSENSE
P11	AMER1	c.1183G>T(p.E395*)	STOP GAINED
P11	PREX2	c.39C>A(p.S13R)	MISSENSE
P11	TP53	c.532C>G(p.H178D)	MISSENSE
P12	SMAD4	c.956-7_965dupCCTATA	FRAMESHIFT
P12	APC	c.3904delC(p.L1302Cfs*	FRAMESHIFT
P12	SMARCA4	c.1538G>A(p.R513Q)	MISSENSE
P12	ERBB3	c.1921C>G(p.H641D)	MISSENSE
P12	TP53	c.707A>G(p.Y236C)	MISSENSE
P13	ERBB3	c.310G>A(p.V104M)	MISSENSE
P13	GNAS	c.1163C>T(p.A388V)	MISSENSE
P13	SETBP1	c.3233C>T(p.T1078M)	MISSENSE
P14	APC	c.4616C>A(p.S1539*)	STOP GAINED
P14	LHCGR	c.459-1G>C	SPLICE
P14	TP53	c.1024C>T(p.R342*)	STOP GAINED
P14	TP53	c.734G>T(p.G245V)	MISSENSE
P14	NRG1	c.1757G>T(p.R586I)	MISSENSE
P14	SMAD4	c.739G>T(p.G247*)	STOP GAINED
P14	ATM	c.829G>T(p.E277*)	STOP GAINED
P14	SMAD4		Deletion
P14	DLL3	c.133G>T(p.A45S)	MISSENSE
P14	SMAD4		Deletion
P14	MEN1		Deletion
P15	TP53	c.481G>A(p.A161T)	MISSENSE
P15	MEN1	c.1012C>G(p.L338V)	MISSENSE
P15	APC	c.637C>T(p.R213*)	STOP GAINED
P15	LRP1B	c.10057+1G>A	SPLICE
P15	ATM	c.979G>A(p.G327R)	MISSENSE
P15	APC	c.4298delC(p.P1433Qfs	FRAMESHIFT

P15	TP53		Deletion
P15	TP53		Deletion
P15	RECQL4		Amplication
P16	MYC		Amplication
P16	APC	c.4351G>T(p.E1451*)	STOP GAINED
P16	TP53	c.817C>T(p.R273C)	MISSENSE
P16	AMER1	c.519dupT(p.S174*)	FRAMESHIFT
P16	FLT4	c.2287G>A(p.V763M)	MISSENSE
P16	PPARD	c.170A>G(p.Q57R)	MISSENSE
P16	MYC		Amplication
P17	ALK	c.389G>A(p.G130D)	MISSENSE
P17	KRAS	c.351A>T(p.K117N)	MISSENSE
P17	GRM3	c.1114G>A(p.V372I)	MISSENSE
P17	DOT1L	c.1391C>A(p.P464H)	MISSENSE
P17	FAT1	c.1645C>G(p.R549G)	MISSENSE
P17	TP53	c.742C>T(p.R248W)	MISSENSE
P17	KIT	c.1415T>C(p.L472P)	MISSENSE
P17	MRE11A	c.233T>C(p.L78S)	MISSENSE
P17	GNAS	c.601C>T(p.R201C)	MISSENSE
P17	CYP2D6	c.994G>A(p.E332K)	MISSENSE
P17	ATRX	c.5831G>A(p.S1944N)	MISSENSE
P17	PRDM1	c.236C>T(p.A79V)	MISSENSE
P17	DPYD	c.2906A>G(p.Q969R)	MISSENSE
P17	KRAS	c.182A>G(p.Q61R)	MISSENSE
P18	TP53		Deletion
P18	MYC		Amplication
P18	APC	c.2863G>T(p.E955*)	STOP GAINED
P18	RET	c.2498G>A(p.R833H)	MISSENSE
P18	IGF1R		Amplication
P18	ZNF703		Amplication
P18	APC	c.4313delC(p.T1438Nfs	FRAMESHIFT
P18	FBXW7	c.1122+1G>C	SPLICE
P18	TP53	c.743G>A(p.R248Q)	MISSENSE
P18	KRAS	c.35G>A(p.G12D)	MISSENSE
P18	TP53		Deletion
P18	IGF1R		Amplication
P18	MYC		Amplication
P18	ZNF703		Amplication
P18	RECQL4		Amplication
P19	APC	c.3907C>T(p.Q1303*)	STOP GAINED
P19	NFE2L2	c.1346G>A(p.R449H)	MISSENSE
P19	TP53	c.637C>T(p.R213*)	STOP GAINED
P19	GNAS		Amplication
P20	BRCA2	c.2649C>G(p.F883L)	MISSENSE
P20	ZNF703	c.698A>G(p.N233S)	MISSENSE
P20	TP53	c.721delT(p.S241Pfs*6)	FRAMESHIFT
P20	BLM	c.1959G>T(p.K653N)	MISSENSE

P20	CYP2A6	c.1035C>A(p.D345E)	MISSENSE
P20	LRP1B	c.2380G>T(p.G794C)	MISSENSE
P20	APC	c.4031C>A(p.S1344*)	STOP GAINED
P21	APC	c.2938A>T(p.K980*)	STOP GAINED
P21	TP53	c.527G>T(p.C176F)	MISSENSE
P21	ERBB3	c.310G>A(p.V104M)	MISSENSE
P21	APC	c.4354delG(p.V1452Yfs	FRAMESHIFT
P21	PBRM1	c.862G>T(p.E288*)	STOP GAINED
P22	PDCD1	c.787G>A(p.A263T)	MISSENSE
P22	SRC		Amplication
P22	BMPR1A		Deletion
P22	KEAP1	c.274C>A(p.Q92K)	MISSENSE
P22	KRAS	c.35G>C(p.G12A)	MISSENSE
P22	APC	c.694C>T(p.R232*)	STOP GAINED
P22	PTEN		Deletion
P22	SMAD4		Deletion
P22	APC	c.2054G>A(p.W685*)	STOP GAINED
P22	TOP1		Amplication
P22	TP53	c.524G>A(p.R175H)	MISSENSE
P22	TP53		Deletion
P22	SRC		Amplication
P22	TOP1		Amplication
P22	SMAD4		Deletion
P22	BMPR1A		Deletion
P22	PTEN		Deletion
P22	SMAD2		Deletion
P22	MAX		Deletion
P22	RAD51B		Deletion
P22	SMAD3		Deletion
P22	PDK1		Amplication
P23	TP53	c.256_257delGC(p.A86	FRAMESHIFT
P23	MITF	c.1019C>T(p.T340I)	MISSENSE
P23	KRAS	c.38G>A(p.G13D)	MISSENSE
P23	ERCC5	c.182G>A(p.R61Q)	MISSENSE
P23	APC	c.2397T>G(p.Y799*)	STOP GAINED
P24	CDK8	c.647-1G>T	SPLICE
P24	APC	c.1495C>T(p.R499*)	STOP GAINED
P24	MLH1		Deletion
P24	SMAD4		Deletion
P24	LRP1B	c.13610C>T(p.A4537V)	MISSENSE
P24	APC	c.4108A>T(p.K1370*)	STOP GAINED
P24	TP53	c.821T>C(p.V274A)	MISSENSE
P24	TSC2	c.3257G>T(p.G1086V)	MISSENSE
P24	SMAD4		Deletion
P24	MLH1		Deletion
P25	BRAF	c.1799T>A(p.V600E)	MISSENSE
P25	TP53	c.841G>C(p.D281H)	MISSENSE

P25	RNF43	c.143T>A(p.I48N)	MISSENSE
P26	TP53	c.733G>A(p.G245S)	MISSENSE
P26	GRIN2A	c.526_534dupATCTTCC	INFRAME INDEL
P26	PKHD1	c.5134G>A(p.G1712R)	MISSENSE
P26	APC	c.4132C>T(p.Q1378*)	STOP GAINED
P26	FBXW7	c.1810A>T(p.K604*)	STOP GAINED
P26	EPHA5	c.1364G>A(p.R455Q)	MISSENSE
P27	APC	c.690_696dupTATACGA	FRAMESHIFT
P27	PIK3CA	c.1633G>A(p.E545K)	MISSENSE
P27	ARID2	c.278dupA(p.Y93*)	STOP GAINED
P27	NF1	c.1431delT(p.F477Lfs*2	FRAMESHIFT
P27	ARID2	ARID2:exon15~IGR (dov	FUSION
P27	CREBBP	c.3307C>T(p.R1103*)	STOP GAINED
P27	PRKDC	c.7259C>T(p.S2420L)	MISSENSE
P27	KMT2A	c.5675A>G(p.Y1892C)	MISSENSE
P27	NF1	c.2033dupC(p.I679Dfs*	FRAMESHIFT
P27	ERBB3	c.850G>A(p.G284R)	MISSENSE
P28	KRAS	c.35G>T(p.G12V)	MISSENSE
P28	NTRK3	c.1933C>T(p.R645C)	MISSENSE
P29	PIK3CA	c.1633G>A(p.E545K)	MISSENSE
P29	SMAD3	c.277C>T(p.R93*)	STOP GAINED
P29	KRAS	c.34G>A(p.G12S)	MISSENSE
P29	SMAD2	c.388C>T(p.R130*)	STOP GAINED
P29	CHD8	c.3318A>T(p.E1106D)	MISSENSE
P29	ARID2	c.622G>A(p.G208R)	MISSENSE
P29	APC	c.4467_4471dupACATT	FRAMESHIFT
P30	TP53	c.461G>T(p.G154V)	MISSENSE
P30	APC	c.1495C>T(p.R499*)	STOP GAINED
P30	JARID2	c.2312G>A(p.R771H)	MISSENSE
P31	IGF1R	c.928G>A(p.G310S)	MISSENSE
P31	SDHD		Deletion
P31	PKHD1	c.1302A>T(p.E434D)	MISSENSE
P31	AR	c.2306T>A(p.L769Q)	MISSENSE
P31	SMAD4		Deletion
P31	TP53	c.638G>C(p.R213P)	MISSENSE
P31	BRAF	c.823G>A(p.E275K)	MISSENSE
P31	SOX2	c.859G>A(p.A287T)	MISSENSE
P31	DOT1L	c.2927C>T(p.A976V)	MISSENSE
P31	APC	c.2932C>T(p.Q978*)	STOP GAINED
P31	HGF	c.1048C>T(p.R350*)	STOP GAINED
P31	KRAS	c.436G>A(p.A146T)	MISSENSE
P31	SMAD4		Deletion
P31	AXIN2		Deletion
P31	FBXW7		Deletion
P32	IL7R		Amplication
P32	APC	c.2821G>T(p.E941*)	STOP GAINED
P32	PARK2	c.1061G>A(p.G354E)	MISSENSE

P32	TOP1		Amplication
P32	SRC		Amplication
P32	APC	c.1409-2A>G	SPLICE
P32	ZNF217		Amplication
P32	LRP1B	c.5016A>C(p.E1672D)	MISSENSE
P32	FAT1	c.10353A>C(p.E3451D)	MISSENSE
P32	ARID1B	c.59G>A(p.G20D)	MISSENSE
P32	CHEK1	c.1115G>A(p.R372Q)	MISSENSE
P32	BAP1	c.1289G>C(p.S430T)	MISSENSE
P32	TP53	c.742C>T(p.R248W)	MISSENSE
P32	GNAS		Amplication
P32	EPHA5	c.488G>T(p.G163V)	MISSENSE
P32	ATM	c.1009C>T(p.R337C)	MISSENSE
P33	PKHD1	c.1946C>T(p.T649M)	MISSENSE
P33	TSC2	c.1459dupT(p.S487Ffs*	FRAMESHIFT
P33	APC	c.3944C>A(p.S1315*)	STOP GAINED
P33	TP53	c.814G>T(p.V272L)	MISSENSE
P34	TP53	c.158G>A(p.W53*)	STOP GAINED
P34	FBXW7	c.832C>T(p.R278*)	STOP GAINED
P34	APC	c.4666dupA(p.T1556Nf	FRAMESHIFT
P34	GATA4	c.770C>T(p.P257L)	MISSENSE
P34	RECQL4	c.654_655delACinsTT(p	MISSENSE
P34	PDGFRB	c.1998C>A(p.N666K)	MISSENSE
P34	MAP3K1	c.2512T>A(p.L838I)	MISSENSE
P34	KRAS	c.38G>A(p.G13D)	MISSENSE
P34	PGR	PGR:exon4~IGR (downs	FUSION
P35	MITF	c.124G>A(p.G42R)	MISSENSE
P35	MED12	c.514G>A(p.E172K)	MISSENSE
P35	SOX2	c.419C>T(p.A140V)	MISSENSE
P35	KRAS	c.35G>T(p.G12V)	MISSENSE
P35	GNAS		Amplication
P35	GATA3	c.619G>A(p.A207T)	MISSENSE
P35	TP53	c.733G>A(p.G245S)	MISSENSE
P35	APC	c.3904delC(p.L1302Cfs*	FRAMESHIFT
P35	FLCN	c.1285dupC(p.H429Pfs*	FRAMESHIFT
P35	GNAS		Amplication
P36	ERBB2	c.2524G>A(p.V842I)	MISSENSE
P36	SMAD3	c.1102C>T(p.R368*)	STOP GAINED
P36	TERT	c.1358G>A(p.R453H)	MISSENSE
P36	APC	c.4473delT(p.F1491Lfs*	FRAMESHIFT
P36	LRP1B	c.2962G>A(p.D988N)	MISSENSE
P36	KRAS	c.35G>A(p.G12D)	MISSENSE
P36	TP53	c.578A>T(p.H193L)	MISSENSE
P36	ARID1A	c.2720C>G(p.S907C)	MISSENSE
P36	CTNNB1	c.832G>A(p.G278R)	MISSENSE
P36	CHEK2	c.283C>T(p.R95*)	STOP GAINED
P37	TP53	c.743G>A(p.R248Q)	MISSENSE

P37	APC	c.4312delA(p.T1438Hfs	FRAMESHIFT
P37	KRAS	c.35G>A(p.G12D)	MISSENSE
P37	APC	c.3502G>T(p.E1168*)	STOP GAINED
P37	RET	c.2906G>A(p.R969Q)	MISSENSE
P38	B2M	c.106G>T(p.E36*)	STOP GAINED
P38	CHD4	c.5720A>G(p.Q1907R)	MISSENSE
P38	JARID2	c.1186dupG(p.A396Gfs	FRAMESHIFT
P38	IGF1R	c.95-2A>G	SPLICE
P38	EPHA3	c.649C>T(p.P217S)	MISSENSE
P38	IGF1R	c.3769C>T(p.P1257S)	MISSENSE
P38	KMT2D	c.4168delG(p.A1390Qfs	FRAMESHIFT
P38	TERT	c.3032G>T(p.R1011M)	MISSENSE
P38	THADA	c.2993G>A(p.R998Q)	MISSENSE
P38	IDH1	c.664C>T(p.R222C)	MISSENSE
P38	NBN	c.1396delA(p.R466Gfs*	FRAMESHIFT
P38	ARID1A	c.5548dupG(p.D1850Gf	FRAMESHIFT
P38	TGFBR2	c.382_383delAA(p.K12{	FRAMESHIFT
P38	BTK	c.843G>A(p.W281*)	STOP GAINED
P38	B2M	c.68-2A>G	SPLICE
P38	POLD1	c.644C>T(p.A215V)	MISSENSE
P38	MED12	c.4070G>A(p.R1357H)	MISSENSE
P38	PRSS1	c.93G>T(p.E31D)	MISSENSE
P38	PTCH1	c.474G>A(p.M158I)	MISSENSE
P38	FANCC	c.554G>A(p.R185Q)	MISSENSE
P38	SMARCA4	c.326delC(p.P109Rfs*1!	FRAMESHIFT
P38	EPHA3	c.369C>A(p.Y123*)	STOP GAINED
P38	KMT2A	c.5665C>T(p.R1889C)	MISSENSE
P38	AKT1	c.652C>A(p.Q218K)	MISSENSE
P38	PAK3	c.413T>C(p.M138T)	MISSENSE
P38	EPHA2	c.1952G>A(p.G651D)	MISSENSE
P38	PIK3CA	c.3140A>G(p.H1047R)	MISSENSE
P38	TSHR	c.290_292delTCT(p.F97	INFRAME INDEL
P38	RUNX1T1	c.1069C>T(p.R357W)	MISSENSE
P38	TP53	c.524G>A(p.R175H)	MISSENSE
P38	MECOM	c.1839delA(p.G614Efs*	FRAMESHIFT
P38	RNF43	c.2136_2143delAGAAA	FRAMESHIFT
P38	BRCA2	c.6539dupT(p.L2180Ffs	FRAMESHIFT
P38	DNMT3A	c.2311C>T(p.R771*)	STOP GAINED
P38	CHD8	c.7543G>T(p.G2515C)	MISSENSE
P38	TERT	c.1453C>T(p.R485C)	MISSENSE
P38	PLCB4	c.1536delC(p.E513Nfs*	FRAMESHIFT
P38	RAD50	c.2164_2165delAA(p.K7	FRAMESHIFT
P38	APC	c.1660C>T(p.R554*)	STOP GAINED
P38	GRIN2A	c.1310G>A(p.R437Q)	MISSENSE
P38	FLT4	c.491G>A(p.G164D)	MISSENSE
P38	MEN1	c.1310C>T(p.A437V)	MISSENSE
P38	TSC1	c.3127_3129dupAGC(p.	INFRAME INDEL

P38	ARAF	c.23delC(p.P8Lfs*26)	FRAMESHIFT
P38	BAX	c.265delC(p.R89Efs*44)	FRAMESHIFT
P38	TTF1	c.821delA(p.K274Sfs*1	FRAMESHIFT
P38	BAX	c.121delG(p.E41Rfs*19	FRAMESHIFT
P38	BUB1B	c.2996G>A(p.R999Q)	MISSENSE
P38	PBRM1	c.1605G>T(p.E535D)	MISSENSE
P38	NRAS	c.34G>T(p.G12C)	MISSENSE
P38	GRIN2A	c.391G>A(p.A131T)	MISSENSE
P38	KMT2B	c.5159T>C(p.L1720S)	MISSENSE
P38	PIK3R1	c.907C>T(p.P303S)	MISSENSE
P38	THADA	c.483T>A(p.N161K)	MISSENSE
P38	GATA6	c.996_998dupCCA(p.H3	INFRAME INDEL
P38	APC	c.3709_3710delCA(p.Q	FRAMESHIFT
P38	KMT2B	c.6895delC(p.R2299Gfs	FRAMESHIFT
P38	ERCC4	c.1024delA(p.R342Gfs*	FRAMESHIFT
P38	NBN	c.2232delT(p.F744Lfs*7	FRAMESHIFT
P38	APC	c.1742delA(p.K581Rfs*	FRAMESHIFT
P38	TP53	c.1085G>A(p.S362N)	MISSENSE
P38	RAF1	c.779C>T(p.T260I)	MISSENSE
P38	TOP2A	c.4303delA(p.R1435Gfs	FRAMESHIFT
P38	SLC3A2	c.899delA(p.K300Rfs*3	FRAMESHIFT
P38	EPAS1	c.2542G>A(p.V848M)	MISSENSE
P38	POLD1	c.1922C>A(p.P641H)	MISSENSE
P38	PIK3R1	c.1344delA(p.K448Nfs*	FRAMESHIFT
P38	SOS1	c.1705C>A(p.L569M)	MISSENSE
P38	ARID2	c.1767T>A(p.N589K)	MISSENSE
P38	RET	c.2161C>T(p.R721W)	MISSENSE
P38	PALLD	c.2756A>G(p.D919G)	MISSENSE
P38	PTCH1	c.3606delC(p.S1203Afs	FRAMESHIFT
P38	KMT2B	c.7795delG(p.E2599Rfs	FRAMESHIFT
P38	PRDM1	c.1571C>T(p.T524M)	MISSENSE
P38	RECQL4	c.645G>T(p.E215D)	MISSENSE
P38	GSTT1	c.566C>T(p.P189L)	MISSENSE
P38	SMAD2	c.524delT(p.L175Cfs*5)	FRAMESHIFT
P38	MSH6	c.3261dupC(p.F1088Lfs	FRAMESHIFT
P38	PREX2	c.2715+1G>A	SPLICE
P38	TP53	c.733G>A(p.G245S)	MISSENSE
P38	DNMT3A	c.81G>T(p.E27D)	MISSENSE
P38	DDR2	c.2507C>T(p.T836M)	MISSENSE
P38	QKI	c.401delA(p.K134Rfs*1	FRAMESHIFT
P38	SOCS1	c.150delC(p.G51Afs*34	FRAMESHIFT
P38	NSD1	c.4151G>T(p.R1384L)	MISSENSE
P38	CHD8	c.3478C>T(p.R1160C)	MISSENSE
P38	RNF43	c.1976delG(p.G659Vfs*	FRAMESHIFT
P39	TP53	c.733G>A(p.G245S)	MISSENSE
P39	APC	c.4099C>T(p.Q1367*)	STOP GAINED
P40	LHCGR	c.1613C>T(p.A538V)	MISSENSE

P40	BTG2	c.403C>A(p.L135I)	MISSENSE
P40	TUBB3	c.772G>A(p.V258M)	MISSENSE
P40	CHD8	c.2827C>T(p.R943C)	MISSENSE
P40	CTNNB1	c.673C>T(p.R225C)	MISSENSE
P40	RECQL4	c.2637delC(p.S880Qfs*	FRAMESHIFT
P40	FBXW7	c.1810A>G(p.K604E)	MISSENSE
P40	GNAS	c.1792C>T(p.R598C)	MISSENSE
P40	CREBBP	c.3631_3632delTT(p.L1	FRAMESHIFT
P40	CBL	c.1852C>T(p.H618Y)	MISSENSE
P40	EP300	c.1992G>T(p.M664I)	MISSENSE
P40	NOTCH2	c.1862G>A(p.R621H)	MISSENSE
P40	TNFAIP3	c.2274delC(p.K759Sfs*	FRAMESHIFT
P40	STK11	c.644G>A(p.G215D)	MISSENSE
P40	FGF19	c.313G>A(p.A105T)	MISSENSE
P40	BAP1	c.1850G>T(p.R617M)	MISSENSE
P40	NOTCH1	c.7475C>T(p.S2492L)	MISSENSE
P40	SUFU	c.71delC(p.P24Rfs*72)	FRAMESHIFT
P40	RPTOR	c.3439G>A(p.V1147I)	MISSENSE
P40	GNAS	c.1066C>T(p.R356C)	MISSENSE
P40	SDHB	c.427T>G(p.L143V)	MISSENSE
P40	IRF2	c.893C>A(p.P298Q)	MISSENSE
P40	GATA6	c.572C>A(p.T191N)	MISSENSE
P40	PPARD	c.856G>A(p.V286M)	MISSENSE
P40	FOXA1	c.1034delG(p.G345Afs*	FRAMESHIFT
P40	MET	c.1235G>A(p.R412H)	MISSENSE
P40	JARID2	c.1272delG(p.R425Gfs*	FRAMESHIFT
P40	SETD2	c.4219delA(p.R1407Gfs	FRAMESHIFT
P40	BRCA2	c.1813dupA(p.I605Nfs*	FRAMESHIFT
P40	STAT3	c.1013T>C(p.V338A)	MISSENSE
P40	JAK1	c.1594C>T(p.R532C)	MISSENSE
P40	JAK1	c.1289delC(p.P430Rfs*	FRAMESHIFT
P40	FAT1	c.8799delA(p.G2934Vfs	FRAMESHIFT
P40	PDCD1	c.105dupC(p.T36Hfs*7	FRAMESHIFT
P40	KDM5A	c.3649C>T(p.R1217W)	MISSENSE
P40	TOP2A	c.3304G>A(p.E1102K)	MISSENSE
P40	FGFR4	c.1944+2T>C	SPLICE
P40	UGT1A1	c.1558A>G(p.K520E)	MISSENSE
P40	TNFAIP3	c.1805C>T(p.T602M)	MISSENSE
P40	FGFR3	c.1637C>T(p.T546M)	MISSENSE
P40	MCL1	c.328_330delGAG(p.E1	INFRAME INDEL
P40	BAX	c.121delG(p.E41Rfs*19	FRAMESHIFT
P40	APC	c.2369_2370delGA(p.R	FRAMESHIFT
P40	PGR	c.1699dupT(p.C567Lfs*	FRAMESHIFT
P40	APC	c.694C>T(p.R232*)	STOP GAINED
P40	NOTCH2	c.3461C>T(p.A1154V)	MISSENSE
P40	NBN	c.1066G>A(p.A356T)	MISSENSE
P40	ROS1	c.1165G>A(p.V389I)	MISSENSE

P40	KRAS	c.38G>A(p.G13D)	MISSENSE
P40	RAD54L	c.1718G>A(p.S573N)	MISSENSE
P40	DENND1A	c.2783G>A(p.R928H)	MISSENSE
P40	FANCF	c.265T>C(p.C89R)	MISSENSE
P40	PIK3CA	c.2189T>C(p.V730A)	MISSENSE
P40	TGFBR2	c.382_383delAA(p.K12[illegible]	FRAMESHIFT
P40	FGFR3	c.1931A>G(p.N644S)	MISSENSE
P40	MLH3	c.1755dupA(p.E586Rfs[illegible]	FRAMESHIFT
P40	SKP2	c.686dupA(p.N229Kfs*[illegible]	FRAMESHIFT
P40	ARID1A	c.827delG(p.G276Efs*8	FRAMESHIFT
P40	CBL	c.2389A>G(p.S797G)	MISSENSE
P40	FAT1	c.223G>A(p.V75I)	MISSENSE
P40	GATA6	c.767C>T(p.A256V)	MISSENSE
P41	TTF1	c.821dupA(p.S275Vfs*[illegible]	FRAMESHIFT
P41	PDE11A	c.1865C>T(p.A622V)	MISSENSE
P41	ARID1A	c.488C>T(p.A163V)	MISSENSE
P41	FANCM	c.4005delA(p.V1336Lfs[illegible]	FRAMESHIFT
P41	PTCH1	c.3921delC(p.R1308Efs[illegible]	FRAMESHIFT
P41	KDM5A	c.3597delA(p.G1200Dfs	FRAMESHIFT
P41	DICER1	c.5186C>T(p.P1729L)	MISSENSE
P41	RAD50	c.1504delA(p.M502Wfs	FRAMESHIFT
P41	LRP1B	c.5584A>G(p.T1862A)	MISSENSE
P41	DNMT3A	c.1742G>T(p.W581L)	MISSENSE
P41	THADA	c.3411G>C(p.Q1137H)	MISSENSE
P41	MAP3K4	c.925G>A(p.A309T)	MISSENSE
P41	TGFBR2	c.382_383delAA(p.K12[illegible]	FRAMESHIFT
P41	MSH2	c.746delA(p.K249Rfs*5	FRAMESHIFT
P41	BRCA2	c.7791delA(p.E2598Kfs[illegible]	FRAMESHIFT
P41	B2M	c.41_44delCTCT(p.S14F	FRAMESHIFT
P41	FAT1	c.2T>C(p.M1?)	START LOST
P41	EPHA5	c.466T>G(p.F156V)	MISSENSE
P41	CREBBP	c.6745C>T(p.R2249C)	MISSENSE
P41	DICER1	c.2810G>A(p.R937H)	MISSENSE
P41	HGF	c.1999G>T(p.G667*)	STOP GAINED
P41	MAP3K1	c.2269C>T(p.R757C)	MISSENSE
P41	HGF	c.800C>T(p.P267L)	MISSENSE
P41	NOTCH1	c.2558_2560delTCT(p.F	INFRAME INDEL
P41	PMS2	c.1239delA(p.D414Tfs*	FRAMESHIFT
P41	BRD4	c.118G>A(p.A40T)	MISSENSE
P41	APC	c.637C>T(p.R213*)	STOP GAINED
P41	ERCC5	c.2964+1G>A	SPLICE
P41	INPP4B	c.2621G>A(p.R874Q)	MISSENSE
P41	ATR	c.4456A>G(p.N1486D)	MISSENSE
P41	TP53	c.817C>T(p.R273C)	MISSENSE
P41	CUX1	c.2705G>A(p.R902H)	MISSENSE
P41	RRM1	c.1985G>A(p.C662Y)	MISSENSE
P41	B2M	c.43_44delCT(p.L15Ffs*	FRAMESHIFT

P41	JARID2	c.1272delG(p.R425Gfs*	FRAMESHIFT
P41	APC	c.4396G>T(p.G1466*)	STOP GAINED
P41	CHD4	c.223-1G>A	SPLICE
P41	ERBB4	c.215G>A(p.R72Q)	MISSENSE
P41	RRM1	c.2052G>T(p.W684C)	MISSENSE
P41	ARID1A	c.3281dupA(p.Q1095Af	FRAMESHIFT
P41	RB1	c.219_220dupAG(p.A74	FRAMESHIFT
P41	TET2	c.1106G>A(p.R369Q)	MISSENSE
P41	CDC73	c.1537C>T(p.R513W)	MISSENSE
P41	MLH1	c.839A>G(p.Y280C)	MISSENSE
P41	EP300	c.6490delC(p.Q2164Rfs	FRAMESHIFT
P41	BAX	c.121delG(p.E41Rfs*19	FRAMESHIFT
P41	CTCF	c.610dupA(p.T204Nfs*2	FRAMESHIFT
P41	SLC3A2	c.899delA(p.K300Rfs*3	FRAMESHIFT
P41	KRAS	c.38G>A(p.G13D)	MISSENSE
P41	FANCI	c.2565A>G(p.I855M)	MISSENSE
P41	FLCN	c.1285delC(p.H429Tfs*.	FRAMESHIFT
P41	KMT2B	c.3159delG(p.R1055Gfs	FRAMESHIFT
P41	AXIN2	c.1994delG(p.G665Afs*	FRAMESHIFT
P42	KRAS		Amplication
P42	BMPR1A		Deletion
P42	ZNF703		Amplication
P42	KRAS	c.436G>A(p.A146T)	MISSENSE
P42	PTEN		Deletion
P42	PLK1	c.878G>A(p.R293H)	MISSENSE
P42	APC	c.3916G>T(p.E1306*)	STOP GAINED
P42	FGFR1		Amplication
P42	RB1	c.1253G>C(p.R418T)	MISSENSE
P42	MYC		Amplication
P42	PTK2		Amplication
P42	CSF1R	c.2881C>T(p.Q961*)	STOP GAINED
P42	NSD1	c.1894C>T(p.R632*)	STOP GAINED
P42	TP53	c.1024C>T(p.R342*)	STOP GAINED
P42	MET	c.3856G>T(p.D1286Y)	MISSENSE
P42	WRN	c.2569C>T(p.R857C)	MISSENSE
P42	DICER1	c.2930A>G(p.N977S)	MISSENSE
P42	KMT2B	c.7907_7908delTT(p.F2	FRAMESHIFT
P42	FGFR1		Amplication
P42	KRAS		Amplication
P42	ZNF703		Amplication
P42	PTK2		Amplication
P42	MYC		Amplication
P42	PTEN		Deletion
P42	BMPR1A		Deletion
P42	RECQL4		Amplication
P42	SMAD2		Deletion
P43	APC	c.2589C>A(p.Y863*)	STOP GAINED

P43	ARID1A	c.529C>T(p.Q177*)	STOP GAINED
P43	PALLD	c.3214C>T(p.Q1072*)	STOP GAINED
P43	KRAS	c.35G>A(p.G12D)	MISSENSE
P44	FANCM	c.5135C>A(p.S1712Y)	MISSENSE
P44	ERBB3	c.179T>A(p.M60K)	MISSENSE
P44	ERBB3	c.2029A>G(p.M677V)	MISSENSE
P44	NOTCH1	c.1066T>A(p.S356T)	MISSENSE
P44	FANCI	c.428A>G(p.N143S)	MISSENSE
P44	BRAF	c.712-2delA	SPLICE
P44	POT1	c.559A>C(p.T187P)	MISSENSE
P44	CDK12	c.754C>T(p.R252*)	STOP GAINED
P44	PDE11A	c.1238G>A(p.R413Q)	MISSENSE
P44	SMARCA4	c.4270C>T(p.P1424S)	MISSENSE
P44	CHEK2	c.1283C>G(p.S428C)	MISSENSE
P45	AXIN2	c.32dupC(p.D12Gfs*8)	FRAMESHIFT
P45	TTF1	c.269G>A(p.R90K)	MISSENSE
P45	APC	c.637C>T(p.R213*)	STOP GAINED
P45	TP53	c.818G>A(p.R273H)	MISSENSE
P45	CDKN1B		Deletion
P46	ALK	c.4198G>T(p.E1400*)	STOP GAINED
P46	MSH6	c.3964G>T(p.E1322*)	STOP GAINED
P46	APC	c.854A>G(p.D285G)	MISSENSE
P46	PTPN13	c.1570A>C(p.I524L)	MISSENSE
P46	ZNF217	c.227A>G(p.H76R)	MISSENSE
P46	EPHA5	c.1345G>A(p.D449N)	MISSENSE
P46	STAT3	c.1875G>T(p.E625D)	MISSENSE
P46	NPM1	c.859C>A(p.L287I)	MISSENSE
P46	KRAS	c.292G>T(p.E98*)	STOP GAINED
P46	TTF1	c.2344G>T(p.E782*)	STOP GAINED
P46	FAT1	c.241A>C(p.N81H)	MISSENSE
P46	PREX2	c.2629A>C(p.I877L)	MISSENSE
P46	BUB1B	c.1246C>T(p.R416W)	MISSENSE
P46	CYP2A6	c.241C>G(p.L81V)	MISSENSE
P46	FLT1	c.2306C>T(p.A769V)	MISSENSE
P46	KRAS	c.436G>A(p.A146T)	MISSENSE
P46	RICTOR	c.839G>A(p.R280Q)	MISSENSE
P46	PRKDC	c.7060A>C(p.I2354L)	MISSENSE
P46	GATA1	c.182C>A(p.A61D)	MISSENSE
P46	SPRED1	c.470T>C(p.F157S)	MISSENSE
P46	QKI	c.10G>A(p.E4K)	MISSENSE
P46	BRAF	c.1165C>T(p.R389C)	MISSENSE
P46	LRP1B	c.3514C>A(p.L1172I)	MISSENSE
P46	SMAD4	c.919G>T(p.E307*)	STOP GAINED
P46	FAT1	c.4516C>T(p.R1506C)	MISSENSE
P46	SMAD4	c.1487G>A(p.R496H)	MISSENSE
P46	ERBB4	c.283C>T(p.R95C)	MISSENSE
P46	CHD8	c.3207C>A(p.F1069L)	MISSENSE

P46	PDGFRA	c.452G>A(p.R151H)	MISSENSE
P46	B2M	c.119C>A(p.S40*)	STOP GAINED
P46	SGK1	c.275C>A(p.S92Y)	MISSENSE
P46	CYSLTR2	c.944G>A(p.R315K)	MISSENSE
P46	PIK3CA	c.3129G>T(p.M1043I)	MISSENSE
P46	LHCGR	c.1078G>T(p.D360Y)	MISSENSE
P46	SMAD2	c.919A>C(p.N307H)	MISSENSE
P46	TTF1	c.1043C>T(p.A348V)	MISSENSE
P46	CTNNB1	c.2129G>A(p.R710H)	MISSENSE
P46	PIK3R1	c.1042C>T(p.R348*)	STOP GAINED
P46	STMN1	c.88G>T(p.E30*)	STOP GAINED
P46	APC	c.4348C>T(p.R1450*)	STOP GAINED
P46	LRP1B	c.805G>T(p.G269*)	STOP GAINED
P46	AMER1	c.2815G>T(p.E939*)	STOP GAINED
P46	CDC73	c.1450C>T(p.R484C)	MISSENSE
P46	PDGFRA	c.2097G>T(p.E699D)	MISSENSE
P46	FANCM	c.5797C>A(p.L1933I)	MISSENSE
P46	FANCL	c.203G>A(p.R68Q)	MISSENSE
P46	ARID2	c.977C>A(p.S326Y)	MISSENSE
P46	MAP2K1	c.199G>A(p.D67N)	MISSENSE
P46	GNAS	c.409G>T(p.A137S)	MISSENSE
P46	MITF	c.952G>T(p.E318*)	STOP GAINED
P46	PIK3R1	c.1381C>T(p.R461*)	STOP GAINED
P46	RELN	c.4864C>T(p.R1622*)	STOP GAINED
P46	CHD8	c.4744C>T(p.R1582*)	STOP GAINED
P46	ATRX	c.3155A>C(p.K1052T)	MISSENSE
P46	FAT1	c.7513G>A(p.A2505T)	MISSENSE
P46	PREX2	c.311A>C(p.E104A)	MISSENSE
P46	MAP2K4	c.968G>A(p.G323E)	MISSENSE
P46	PIK3R1	c.1892G>A(p.R631Q)	MISSENSE
P46	ATR	c.446T>C(p.L149S)	MISSENSE
P46	EZH2	c.144A>C(p.K48N)	MISSENSE
P46	NF1	c.5588G>T(p.G1863V)	MISSENSE
P46	AR	c.2428G>T(p.A810S)	MISSENSE
P46	ROS1	c.892A>C(p.K298Q)	MISSENSE
P46	GRIN2A	c.3217G>T(p.E1073*)	STOP GAINED
P46	KDM5A	c.4672G>T(p.E1558*)	STOP GAINED
P46	PTK2	c.2652G>T(p.K884N)	MISSENSE
P46	ARID1A	c.4646G>A(p.G1549D)	MISSENSE
P46	FBXW7	c.103C>T(p.R35C)	MISSENSE
P46	PDE11A	c.4G>A(p.A2T)	MISSENSE
P46	TNFRSF11A	c.983C>A(p.S328*)	STOP GAINED
P46	THADA	c.627A>C(p.Q209H)	MISSENSE
P46	NFE2L2	c.1687G>A(p.E563K)	MISSENSE
P46	LRP1B	c.7333G>A(p.D2445N)	MISSENSE
P46	TUBB3	c.185G>A(p.R62Q)	MISSENSE
P46	MAP2K4	c.658A>C(p.K220Q)	MISSENSE

P46	AR	c.1974G>T(p.Q658H)	MISSENSE
P46	ATR	c.7282C>A(p.L2428I)	MISSENSE
P46	TOP2A	c.1683G>A(p.W561*)	STOP GAINED
P46	APC	c.2828C>A(p.S943*)	STOP GAINED
P46	ROS1	c.3730G>T(p.E1244*)	STOP GAINED
P46	ATRX	c.6301G>T(p.E2101*)	STOP GAINED
P46	DPYD	c.2682G>T(p.K894N)	MISSENSE
P46	PKHD1	c.11021G>A(p.S3674N)	MISSENSE
P46	MRE11A	c.42A>C(p.K14N)	MISSENSE
P46	GRIN2A	c.1663G>A(p.A555T)	MISSENSE
P46	TEK	c.2475A>C(p.K825N)	MISSENSE
P46	RAC1	c.73A>T(p.T25S)	MISSENSE
P46	WT1	c.599G>A(p.S200N)	MISSENSE
P46	ABCB1	c.122G>A(p.R41H)	MISSENSE
P46	STAT3	c.148G>T(p.E50*)	STOP GAINED
P46	ATIC	c.1135C>T(p.R379*)	STOP GAINED
P46	ATR	c.3118T>G(p.L1040V)	MISSENSE
P46	LRP1B	c.9086G>A(p.S3029N)	MISSENSE
P46	CCNE1	c.72C>A(p.F24L)	MISSENSE
P46	MET	c.1235G>A(p.R412H)	MISSENSE
P46	ZNF217	c.363G>T(p.K121N)	MISSENSE
P46	PTPN13	c.2480C>A(p.S827Y)	MISSENSE
P46	FANCD2	c.3857A>G(p.D1286G)	MISSENSE
P46	APC	c.6709C>T(p.R2237*)	STOP GAINED
P46	PPP2R1A	c.1627T>C(p.S543P)	MISSENSE
P46	CTNNB1	c.1544G>A(p.R515Q)	MISSENSE
P46	CXCR4	c.421G>A(p.A141T)	MISSENSE
P46	GNAS	c.595C>T(p.R199C)	MISSENSE
P46	CBL	c.2363G>A(p.R788Q)	MISSENSE
P46	MCL1	c.437T>C(p.L146S)	MISSENSE
P46	STAG2	c.2998C>T(p.L1000F)	MISSENSE
P46	ATR	c.442G>T(p.E148*)	STOP GAINED
P46	PIK3CA	c.1624G>A(p.E542K)	MISSENSE
P46	BAD	c.146G>A(p.S49N)	MISSENSE
P46	SMAD4	c.404G>A(p.R135Q)	MISSENSE
P46	MITF	c.217C>T(p.R73C)	MISSENSE
P46	POLE	c.1331T>A(p.M444K)	MISSENSE
P46	EP300	c.2891C>A(p.S964Y)	MISSENSE
P47	DICER1	c.5552G>A(p.R1851H)	MISSENSE
P47	KRAS	c.34G>T(p.G12C)	MISSENSE
P47	APC	c.4486delA(p.T1496Lfs’	FRAMESHIFT
P47	FANCG	c.176-2A>C	SPLICE
P47	LRP1B	c.5037G>C(p.R1679S)	MISSENSE
P47	APC	c.2626C>T(p.R876*)	STOP GAINED
P47	TP63	c.535G>A(p.V179M)	MISSENSE
P47	TP53	c.818G>A(p.R273H)	MISSENSE
P47	PREX2	c.1037C>G(p.P346R)	MISSENSE

P48	RB1	c.2263T>A(p.F755I)	MISSENSE
P48	NOTCH2	c.7255C>A(p.P2419T)	MISSENSE
P48	TAP1	c.1904G>A(p.R635H)	MISSENSE
P48	SPRED1	c.1262T>A(p.V421D)	MISSENSE
P48	LHCGR	c.1664A>G(p.N555S)	MISSENSE
P48	PIK3CA	c.3012G>A(p.M1004I)	MISSENSE
P48	PBRM1	c.2776dupA(p.R926Kfs*	FRAMESHIFT
P48	CHD8	c.4658G>A(p.R1553Q)	MISSENSE
P48	FGFR2	c.308G>A(p.G103D)	MISSENSE
P48	LRP1B	c.4723T>A(p.F1575I)	MISSENSE
P48	EPHA3	c.1306G>A(p.A436T)	MISSENSE
P48	APC	c.2461_2462delGT(p.V8	FRAMESHIFT
P48	ROS1	c.6176T>G(p.L2059R)	MISSENSE
P48	RAD50	c.3163delA(p.S1055Vfs	FRAMESHIFT
P48	RAD50	c.1376dupA(p.N459Kfs	FRAMESHIFT
P48	ERBB2	c.2990C>T(p.A997V)	MISSENSE
P48	JAK1	c.1289dupC(p.L431Vfs*	FRAMESHIFT
P48	JARID2	c.1186dupG(p.A396Gfs	FRAMESHIFT
P48	KMT2A	c.2317_2318dupCC(p.S	FRAMESHIFT
P48	PKHD1	c.11763delA(p.E3922Kf	FRAMESHIFT
P48	MLH3	c.3137G>A(p.R1046Q)	MISSENSE
P48	RNF43	c.1576G>A(p.V526M)	MISSENSE
P48	LZTR1	c.2317G>A(p.V773M)	MISSENSE
P48	CHD8	c.5783G>A(p.R1928Q)	MISSENSE
P48	PMS2	c.1239dupA(p.D414Rfs	FRAMESHIFT
P48	APC	c.646C>T(p.R216*)	STOP GAINED
P48	SMO	c.635G>A(p.G212D)	MISSENSE
P48	MSH6	c.3260_3261dupCC(p.F	FRAMESHIFT
P48	FLCN	c.1285dupC(p.H429Pfs	FRAMESHIFT
P48	BUB1B	c.2488G>A(p.D830N)	MISSENSE
P48	IGF2	c.536G>A(p.R179Q)	MISSENSE
P48	RARA	c.574C>T(p.R192C)	MISSENSE
P48	LZTR1	c.1217C>T(p.T406M)	MISSENSE
P48	FLCN	c.1285delC(p.H429Tfs*	FRAMESHIFT
P48	ATM	c.8704A>G(p.T2902A)	MISSENSE
P48	B2M	c.293A>G(p.Y98C)	MISSENSE
P48	AIP	c.325G>A(p.A109T)	MISSENSE
P48	IFNGR2	c.961T>C(p.S321P)	MISSENSE
P48	PMS2	c.353G>A(p.S118N)	MISSENSE
P48	ARID1A	c.936dupC(p.G313Rfs*8	FRAMESHIFT
P48	MSH6	c.3261dupC(p.F1088Lfs	FRAMESHIFT
P48	ERBB2	c.2524G>A(p.V842I)	MISSENSE
P48	ASXL1	c.1934dupG(p.G646Wfs	FRAMESHIFT
P48	MUTYH	c.389-2A>C	SPLICE
P48	ATR	c.6700A>G(p.S2234G)	MISSENSE
P48	PIK3R2	c.1712G>T(p.R571L)	MISSENSE
P48	PMS1	c.2766delT(p.H923Ifs*3	FRAMESHIFT

P49	RUNX1	c.318G>A(p.W106*)	STOP GAINED
P49	KRAS	c.35G>T(p.G12V)	MISSENSE
P49	CHD4	CHD4:exon30~IGR (ups	FUSION
P49	TP53	c.844C>T(p.R282W)	MISSENSE
P49	KMT2C	c.2759dupT(p.L921Ifs*1	FRAMESHIFT
P49	APC	c.2413C>T(p.R805*)	STOP GAINED
P50	ASXL1	c.1934dupG(p.G646Wfs	FRAMESHIFT
P50	ATM	c.8190G>T(p.Q2730H)	MISSENSE
P50	MTOR	c.6422C>A(p.P2141Q)	MISSENSE
P50	APC	c.694C>T(p.R232*)	STOP GAINED
P50	ERBB4	c.671delC(p.P224Lfs*27	FRAMESHIFT
P50	AMER1	c.2156T>C(p.M719T)	MISSENSE
P50	KRAS	c.35G>T(p.G12V)	MISSENSE
P50	CHD4	c.4018C>T(p.R1340C)	MISSENSE
P51	KRAS	c.35G>T(p.G12V)	MISSENSE
P51	BRAF	c.1781A>G(p.D594G)	MISSENSE
P51	ERBB2	c.2033G>A(p.R678Q)	MISSENSE
P51	FAT1	c.7361G>A(p.R2454Q)	MISSENSE
P51	APC	c.4921G>T(p.V1641L)	MISSENSE
P52	LRP1B	c.7707G>T(p.K2569N)	MISSENSE
P52	APC	c.1660C>T(p.R554*)	STOP GAINED
P52	EPHA3	c.2740C>T(p.R914C)	MISSENSE
P52	WRN	c.1298A>G(p.D433G)	MISSENSE
P52	APC	c.3928A>T(p.K1310*)	STOP GAINED
P52	TP53	c.637C>T(p.R213*)	STOP GAINED
P52	PIK3CA	c.1624G>A(p.E542K)	MISSENSE
P52	KDM5A	c.2356G>A(p.D786N)	MISSENSE
P53	DICER1	c.1219G>T(p.D407Y)	MISSENSE
P53	MTOR	c.7499T>A(p.I2500N)	MISSENSE
P53	PIK3CA	c.1633G>A(p.E545K)	MISSENSE
P53	CTNNB1	c.13+91_181del	SPLICE
P53	KRAS	c.35G>A(p.G12D)	MISSENSE
P54	NRAS	c.182A>T(p.Q61L)	MISSENSE
P54	ARAF	c.133_134del(p.L45Rf	FRAMESHIFT
P54	SMAD4	c.1333C>T(p.R445*)	STOP GAINED
P54	FANCA	c.967A>T(p.T323S)	MISSENSE
P54	RNF43	c.505G>A(p.A169T)	MISSENSE
P54	NTRK2	c.1388G>C(p.G463A)	MISSENSE
P54	TP53	c.818G>A(p.R273H)	MISSENSE
P54	CREBBP	c.2431G>T(p.G811W)	MISSENSE
P55	FBXW7	c.1039C>T(p.R347C)	MISSENSE
P55	SRC		Amplication
P55	APC	c.2351del(p.S784Ffs*	FRAMESHIFT
P55	SKP2	c.127A>T(p.K43*)	STOP GAINED
P55	TERT	c.1291C>A(p.P431T)	MISSENSE
P55	APC	c.1312+2T>C	SPLICE

P55	PGR	c.1867C>T(p.R623C)	MISSENSE
P55	TP53	c.818G>A(p.R273H)	MISSENSE
P55	KRAS	c.35G>T(p.G12V)	MISSENSE
P55	FAT1	c.10431_10433del(p.I	INFRAME INDEL
P55	ZNF217		Amplication
P55	TOP1		Amplication
P56	ALK	c.4460C>T(p.S1487L)	MISSENSE
P56	BUB1B	c.2983A>G(p.K995E)	MISSENSE
P56	RNF43	c.351_352insAC(p.P1	FRAMESHIFT
P56	PIK3CA	c.3129G>T(p.M1043I)	MISSENSE
P56	RNF43	c.350G>C(p.R117P)	MISSENSE
P56	APC	c.637C>T(p.R213*)	STOP GAINED
P56	KIT	c.1588G>A(p.V530I)	MISSENSE
P56	ESR1	c.1712C>T(p.A571V)	MISSENSE
P56	APC	c.3927_3931del(p.E1	FRAMESHIFT
P56	ATM	c.1899-2_1914del	SPLICE
P56	KRAS	c.35G>A(p.G12D)	MISSENSE
P57	EGFR		Amplication
P57	APC	c.1312+1G>A	SPLICE
P57	TP53	c.844C>T(p.R282W)	MISSENSE
P57	RICTOR	RICTOR:exon4~IGR (u	FUSION
P58	ERBB4	c.2479A>G(p.I827V)	MISSENSE
P58	KDM5A	c.4440C>G(p.F1480L)	MISSENSE
P58	BMPR1A	c.1441C>T(p.P481S)	MISSENSE
P58	NOTCH2	c.6498del(p.S2167Lfs	FRAMESHIFT
P58	FANCG	c.1733C>T(p.T578I)	MISSENSE
P58	KRAS	c.35G>A(p.G12D)	MISSENSE
P58	JAK3	c.1511C>T(p.S504F)	MISSENSE
P58	PKHD1	c.1154C>G(p.T385R)	MISSENSE
P58	BMPR1A	c.1362del(p.Q454Hfs	FRAMESHIFT
P58	BTK	c.868C>T(p.Q290*)	STOP GAINED
P58	PTEN	c.976G>C(p.D326H)	MISSENSE
P58	AKT1	c.968G>T(p.D323Y)	MISSENSE
P58	TEK	c.3178C>T(p.P1060S)	MISSENSE
P58	TP53	c.818G>A(p.R273H)	MISSENSE
P58	PAK3	c.118G>C(p.E40Q)	MISSENSE
P58	SPRY4	c.366C>G(p.D122E)	MISSENSE
P58	CUL3	c.765G>T(p.L255F)	MISSENSE
P59	APC	c.694C>T(p.R232*)	STOP GAINED
P59	TP53	c.747G>C(p.R249S)	MISSENSE
P60	FLT1		Amplication
P60	CXCR4	c.575G>A(p.R192H)	MISSENSE
P60	GNAS	c.215C>T(p.S72L)	MISSENSE
P60	FLT3		Amplication

P60	MSH2	c.2008C>T(p.P670S)	MISSENSE
P60	LHCGR	c.1435C>T(p.R479*)	STOP GAINED
P60	TP53	c.535C>T(p.H179Y)	MISSENSE
P60	TP53	c.525_530delinsTG(p.	FRAMESHIFT
P60	MAP2K1	c.171G>T(p.K57N)	MISSENSE
P60	APC	c.4478_4479delinsA(	FRAMESHIFT
P60	APC	c.3897_3907del(p.T1	FRAMESHIFT
P61	CREBBP	c.1063C>T(p.Q355*)	STOP GAINED
P61	TP53	c.733G>A(p.G245S)	MISSENSE
P61	APC	c.2512A>T(p.R838*)	STOP GAINED
P61	BRD4	c.152C>G(p.S51C)	MISSENSE
P61	AKT2	IGR (downstream UQ	FUSION
P61	FBXW7	c.1336_1338del(p.R4	INFRAME INDEL
P61	PPP2R1A	c.547C>T(p.R183W)	MISSENSE
P61	EGFR		Amplication
P61	BRD4	c.196C>A(p.L66M)	MISSENSE
P62	LRP1B	c.3214C>T(p.R1072C)	MISSENSE
P62	CREBBP	c.3349C>A(p.P1117T)	MISSENSE
P62	KRAS	c.38G>A(p.G13D)	MISSENSE
P62	FLCN	c.250_295dup(p.D99	STOP GAINED
P62	NTRK3	c.640G>A(p.V214M)	MISSENSE
P62	ASXL1	c.2077C>T(p.R693*)	STOP GAINED
P62	TP53	c.524G>A(p.R175H)	MISSENSE
P63	PIK3CA	c.1638G>C(p.Q546H)	MISSENSE
P63	KRAS	c.34G>T(p.G12C)	MISSENSE
P63	TP53	c.395A>C(p.K132T)	MISSENSE
P63	APC	c.4666dup(p.T1556N	FRAMESHIFT
P63	AMER1	c.1489C>T(p.R497*)	STOP GAINED
P63	PDE11A	c.1105G>A(p.A369T)	MISSENSE
P63	PKHD1	c.3805G>A(p.V1269M	MISSENSE
P63	NOTCH1	c.2357C>T(p.P786L)	MISSENSE
P63	SMAD4	c.1607T>C(p.L536P)	MISSENSE
P63	APC		Deletion
P64	BRAF	c.1406G>C(p.G469A)	MISSENSE
P64	RECQL4	c.1151G>A(p.R384Q)	MISSENSE
P64	APC	c.4606G>T(p.E1536*)	STOP GAINED
P64	FANCA	c.74T>A(p.L25Q)	MISSENSE
P64	DNMT3A	c.196C>A(p.P66T)	MISSENSE
P64	RNF43	c.1825C>T(p.R609W)	MISSENSE
P64	SMAD4	c.1082G>A(p.R361H)	MISSENSE
P64	TP53	c.817C>T(p.R273C)	MISSENSE
P64	CDC73	c.559G>C(p.A187P)	MISSENSE
P65	BRCA2	c.6952C>T(p.R2318*)	STOP GAINED
P65	TOP1	c.829A>C(p.N277H)	MISSENSE

P65	ATRX	c.3195G>T(p.E1065D)	MISSENSE
P65	TSHR	c.325C>T(p.R109W)	MISSENSE
P65	NTRK1	c.2005T>G(p.F669V)	MISSENSE
P65	NF1	c.4084C>T(p.R1362*)	STOP GAINED
P65	XPC	c.2497G>T(p.E833*)	STOP GAINED
P65	CUL3	c.532G>T(p.E178*)	STOP GAINED
P65	ATRX	c.550G>T(p.D184Y)	MISSENSE
P65	GRIN2A	c.1918A>G(p.I640V)	MISSENSE
P65	FLT4	c.1696G>A(p.E566K)	MISSENSE
P65	DTL	c.612G>T(p.Q204H)	MISSENSE
P65	PREX2	c.2473T>C(p.Y825H)	MISSENSE
P65	AKT3	c.497G>A(p.R166Q)	MISSENSE
P65	RAD54L	c.1062G>T(p.K354N)	MISSENSE
P65	PPP2R1A	c.82C>T(p.R28C)	MISSENSE
P65	APC	c.688C>T(p.R230C)	MISSENSE
P65	IDH1	c.59G>T(p.R20L)	MISSENSE
P65	FAT1	c.5050A>C(p.S1684R)	MISSENSE
P65	MSH2	c.1588G>T(p.E530*)	STOP GAINED
P65	ABCB1	c.160G>T(p.G54*)	STOP GAINED
P65	FBXW7	c.286G>T(p.E96*)	STOP GAINED
P65	CDK12	c.3308-1G>A	SPLICE
P65	EGFR	c.382A>G(p.N128D)	MISSENSE
P65	PDE11A	c.1034T>G(p.I345S)	MISSENSE
P65	LRP1B	c.3041C>A(p.S1014Y)	MISSENSE
P65	BRCA2	c.3459G>T(p.K1153N	MISSENSE
P65	PTEN	c.996A>C(p.K332N)	MISSENSE
P65	DDR2	c.1049A>G(p.Y350C)	MISSENSE
P65	TUBB3	c.795C>A(p.F265L)	MISSENSE
P65	EPAS1	c.910A>C(p.S304R)	MISSENSE
P65	NSD1	c.507G>T(p.K169N)	MISSENSE
P65	LHCGR	c.505T>G(p.F169V)	MISSENSE
P65	NSD1	c.5149C>T(p.R1717C)	MISSENSE
P65	ATM	c.742C>T(p.R248*)	STOP GAINED
P65	FANCC	c.1555A>G(p.T519A)	MISSENSE
P65	GRM8	c.1146G>T(p.K382N)	MISSENSE
P65	KMT2A	c.444C>A(p.F148L)	MISSENSE
P65	GRIN2A	c.3843G>T(p.K1281N	MISSENSE
P65	ROS1	c.6629G>T(p.R2210I)	MISSENSE
P65	SETD2	c.1715C>A(p.S572Y)	MISSENSE
P65	IL7R	c.638T>G(p.F213C)	MISSENSE
P65	CXCR4	c.1056T>G(p.S352R)	MISSENSE
P65	MRE11	c.1561G>T(p.E521*)	STOP GAINED
P65	SMAD2	c.565G>T(p.E189*)	STOP GAINED
P65	SETD2	c.3694A>T(p.K1232*)	STOP GAINED

P65	JUN	c.907G>T(p.E303*)	STOP GAINED
P65	PDE11A	c.1029C>A(p.F343L)	MISSENSE
P65	SETBP1	c.3436C>T(p.R1146W	MISSENSE
P65	NSD1	c.758A>C(p.K253T)	MISSENSE
P65	CYP2D6	c.909C>A(p.F303L)	MISSENSE
P65	GRM3	c.1004G>A(p.R335H)	MISSENSE
P65	APC	c.4199C>T(p.S1400L)	MISSENSE
P65	RAD51C	c.940A>C(p.I314L)	MISSENSE
P65	GATA4	c.775C>T(p.R259C)	MISSENSE
P65	HDAC9	c.1259C>A(p.S420Y)	MISSENSE
P65	WISP3	c.761G>A(p.S254N)	MISSENSE
P65	FAT1	c.10045G>A(p.V3349	MISSENSE
P65	NSD1	c.2101G>A(p.D701N)	MISSENSE
P65	IFNGR2	c.995A>C(p.K332T)	MISSENSE
P65	AKT3	c.1192G>T(p.E398*)	STOP GAINED
P65	TAP1	c.1227C>A(p.Y409*)	STOP GAINED
P65	FAT1	c.2752G>T(p.E918*)	STOP GAINED
P65	PTCH1	c.2145C>A(p.F715L)	MISSENSE
P65	DDR2	c.724T>G(p.F242V)	MISSENSE
P65	BLM	c.1537A>C(p.K513Q)	MISSENSE
P65	RAD51D	c.629C>T(p.A210V)	MISSENSE
P65	ERBB4	c.3668A>G(p.N1223S	MISSENSE
P65	DICER1	c.4928A>G(p.K1643R	MISSENSE
P65	PTK2	c.2123A>C(p.Q708P)	MISSENSE
P65	KDM5A	c.4483A>C(p.K1495Q	MISSENSE
P65	ERBB4	c.3128C>T(p.S1043L)	MISSENSE
P65	KDM5A	c.559G>T(p.D187Y)	MISSENSE
P65	CHD4	c.4657A>G(p.T1553A	MISSENSE
P65	ZNF217	c.2222A>C(p.K741T)	MISSENSE
P65	SRC	c.95T>A(p.F32Y)	MISSENSE
P65	PIK3C3	c.66G>T(p.K22N)	MISSENSE
P65	PIK3R1	c.232C>T(p.R78*)	STOP GAINED
P65	FGFR2	c.2306A>G(p.Y769C)	MISSENSE
P65	PTPN13	c.3396T>G(p.S1132R)	MISSENSE
P65	FLT3	c.2779G>A(p.A927T)	MISSENSE
P65	ERBB4	c.2131C>T(p.R711C)	MISSENSE
P65	ERCC2	c.1264G>T(p.D422Y)	MISSENSE
P65	POLE	c.857C>G(p.P286R)	MISSENSE
P65	DOT1L	c.850T>G(p.L284V)	MISSENSE
P65	PDGFRB	c.2225A>G(p.D742G)	MISSENSE
P65	EMSY	c.566T>C(p.V189A)	MISSENSE
P65	IGF1R	c.625A>C(p.N209H)	MISSENSE
P65	IFNA6	c.282G>T(p.K94N)	MISSENSE
P65	NSD1	c.5278A>G(p.T1760A	MISSENSE

P65	KMT2B	c.1966G>A(p.E656K)	MISSENSE
P65	CEP57	c.1417A>C(p.K473Q)	MISSENSE
P65	AXL	c.1315C>T(p.R439C)	MISSENSE
P65	BRCA1	c.3690A>C(p.L1230F)	MISSENSE
P65	POLH	c.241C>T(p.R81C)	MISSENSE
P65	FAT1	c.922T>C(p.S308P)	MISSENSE
P65	BLM	c.3553T>G(p.L1185V)	MISSENSE
P65	ALK	c.776G>A(p.R259H)	MISSENSE
P65	FANCD2	c.1610G>T(p.S537I)	MISSENSE
P65	CDK8	c.196G>T(p.E66*)	STOP GAINED
P65	FBXW7	c.289G>T(p.E97*)	STOP GAINED
P65	ARID1A	c.1820C>A(p.S607*)	STOP GAINED
P65	ERCC1	c.661G>A(p.D221N)	MISSENSE
P65	KMT2B	c.1430G>T(p.S477I)	MISSENSE
P65	KDR	c.2053G>A(p.E685K)	MISSENSE
P65	SMAD2	c.962G>A(p.R321Q)	MISSENSE
P65	MAP3K1	c.1918T>C(p.S640P)	MISSENSE
P65	ATF1	c.691C>T(p.R231C)	MISSENSE
P65	TERT	c.2264T>G(p.V755G)	MISSENSE
P65	FAT1	c.5177A>G(p.D1726G	MISSENSE
P65	CTNNB1	c.966A>C(p.Q322H)	MISSENSE
P65	ATIC	c.1136G>A(p.R379Q)	MISSENSE
P65	CHD8	c.3214A>G(p.T1072A	MISSENSE
P65	BMPR1A	c.1411C>T(p.R471C)	MISSENSE
P65	ATR	c.2116G>T(p.E706*)	STOP GAINED
P65	TP53	c.637C>T(p.R213*)	STOP GAINED
P65	SETD2	c.2872T>G(p.C958G)	MISSENSE
P65	BRCA2	c.8496G>T(p.E2832D	MISSENSE
P65	EXT2	c.402G>T(p.K134N)	MISSENSE
P65	FBXW7	c.1711C>T(p.R571W)	MISSENSE
P65	ATM	c.3617T>C(p.L1206S)	MISSENSE
P65	PKHD1	c.949G>T(p.D317Y)	MISSENSE
P65	CRKL	c.216G>T(p.K72N)	MISSENSE
P65	EXT1	c.593G>A(p.G198D)	MISSENSE
P65	PLCB4	c.224A>C(p.K75T)	MISSENSE
P65	APC	c.2891T>G(p.L964*)	STOP GAINED
P65	ROS1	c.1931A>C(p.K644T)	MISSENSE
P65	NRG1	c.862C>T(p.R288W)	MISSENSE
P65	NSD1	c.444T>G(p.I148M)	MISSENSE
P65	GRIN2A	c.262G>A(p.D88N)	MISSENSE
P65	PIK3CA	c.263G>A(p.R88Q)	MISSENSE
P65	ERBB4	c.2605G>A(p.E869K)	MISSENSE
P65	APC	c.7862C>A(p.S2621Y)	MISSENSE
P65	LHCGR	c.425C>T(p.T142M)	MISSENSE

P65	APC	c.6077C>A(p.S2026Y)	MISSENSE
P65	BRCA2	c.5063A>C(p.E1688A)	MISSENSE
P65	KDR	c.858G>T(p.K286N)	MISSENSE
P65	TNFSF11	c.940C>T(p.R314*)	STOP GAINED
P65	FANCI	c.250G>T(p.E84*)	STOP GAINED
P65	ATM	c.895G>T(p.E299*)	STOP GAINED
P65	EP300	c.149C>T(p.S50F)	MISSENSE
P65	DENND1A	c.2348C>T(p.A783V)	MISSENSE
P65	DPYD	c.893C>T(p.T298M)	MISSENSE
P65	GRIN2A	c.2954C>T(p.P985L)	MISSENSE
P65	TAP1	c.1372A>C(p.K458Q)	MISSENSE
P65	CHD4	c.4013G>T(p.R1338I)	MISSENSE
P65	MYCL	c.598A>C(p.I200L)	MISSENSE
P65	PREX2	c.4091T>C(p.V1364A)	MISSENSE
P65	CASP8	c.203G>A(p.R68Q)	MISSENSE
P65	KMT2A	c.6185G>A(p.R2062H	MISSENSE
P65	FLT3	c.1826A>G(p.N609S)	MISSENSE
P65	LRP1B	c.8330G>A(p.R2777Q	MISSENSE
P65	ERBIN	c.2179G>T(p.D727Y)	MISSENSE
P65	RICTOR	c.665G>A(p.R222Q)	MISSENSE
P65	MAP2K4	c.433C>T(p.R145W)	MISSENSE
P65	APC	c.2756G>T(p.R919I)	MISSENSE
P65	PBRM1	c.1564C>T(p.R522*)	STOP GAINED
P65	CDK8	c.709C>T(p.R237*)	STOP GAINED
P65	NRG1	c.841G>T(p.D281Y)	MISSENSE
P65	SMO	c.2002C>T(p.R668C)	MISSENSE
P65	CASP8	c.245G>T(p.R82I)	MISSENSE
P65	RICTOR	c.839G>A(p.R280Q)	MISSENSE
P65	ALK	c.1982A>G(p.N661S)	MISSENSE
P65	CREBBP	c.3832G>A(p.E1278K)	MISSENSE
P65	ATR	c.3938A>C(p.K1313T)	MISSENSE
P65	PLCB4	c.2221C>T(p.R741C)	MISSENSE
P65	FLT1	c.3671G>T(p.R1224I)	MISSENSE
P65	CTLA4	c.554C>A(p.S185Y)	MISSENSE
P65	AR	c.689C>T(p.S230L)	MISSENSE
P65	NTRK1	c.2212G>A(p.D738N)	MISSENSE
P65	ERBB4	c.2525G>A(p.R842Q)	MISSENSE
P65	CBLB	c.1529G>A(p.R510H)	MISSENSE
P65	BRD4	c.8C>T(p.A3V)	MISSENSE
P65	SPRED1	c.138A>T(p.K46N)	MISSENSE
P65	POLE	c.671A>G(p.Y224C)	MISSENSE
P65	PRSS1	c.74T>G(p.V25G)	MISSENSE
P65	PKHD1	c.11006C>T(p.S3669L	MISSENSE
P65	PKHD1	c.2900A>G(p.N967S)	MISSENSE

P65	GRIN2A	c.2865T>G(p.I955M)	MISSENSE
P65	WRN	c.2972C>A(p.S991Y)	MISSENSE
P65	LYN	c.266A>C(p.K89T)	MISSENSE
P65	PGR	c.415T>G(p.S139A)	MISSENSE
P65	CTCF	c.766A>C(p.K256Q)	MISSENSE
P65	MAP3K1	c.3420G>A(p.M1140I	MISSENSE
P65	PBRM1	c.2128C>T(p.R710*)	STOP GAINED
P65	ROS1	c.4765G>T(p.E1589*)	STOP GAINED
P65	BRCA2	c.7795G>T(p.E2599*)	STOP GAINED
P65	PIK3R1	c.484C>T(p.R162*)	STOP GAINED
P65	SETD2	c.2258T>C(p.V753A)	MISSENSE
P65	FAT1	c.12847G>A(p.E4283I	MISSENSE
P65	WRN	c.375A>C(p.K125N)	MISSENSE
P65	RICTOR	c.3793A>C(p.K1265Q	MISSENSE
P65	XRCC2	c.346T>G(p.F116V)	MISSENSE
P65	ATR	c.3064A>C(p.N1022H	MISSENSE
P65	RNF43	c.1701G>T(p.R567S)	MISSENSE
P65	PIK3R1	c.880A>G(p.N294D)	MISSENSE
P65	ABCB1	c.788C>A(p.T263N)	MISSENSE
P65	PDGFRB	c.631G>A(p.V211M)	MISSENSE
P65	ASXL1	c.2362G>T(p.E788*)	STOP GAINED
P65	MSH6	c.3586G>T(p.E1196*)	STOP GAINED
P65	TNFAIP3	c.913G>T(p.E305*)	STOP GAINED
P65	TSHR	c.1762A>G(p.T588A)	MISSENSE
P65	PIK3CA	c.1281A>G(p.I427M)	MISSENSE
P65	DPYD	c.2726G>T(p.R909I)	MISSENSE
P65	TERT	c.2684T>C(p.L895P)	MISSENSE
P65	ABCB1	c.2522C>A(p.A841E)	MISSENSE
P65	ZNF217	c.1555G>A(p.E519K)	MISSENSE
P65	PALB2	c.1689G>T(p.K563N)	MISSENSE
P65	PKHD1	c.10822T>G(p.L3608V	MISSENSE
P65	BAX	c.376A>G(p.I126V)	MISSENSE
P65	CHEK2	c.300G>T(p.Q100H)	MISSENSE
P65	GRM3	c.2029G>A(p.A677T)	MISSENSE
P65	RAD50	c.3934C>A(p.H1312N	MISSENSE
P65	KMT2B	c.368T>G(p.F123C)	MISSENSE
P65	PKHD1	c.6001G>A(p.E2001K)	MISSENSE
P65	NSD1	c.1212G>T(p.E404D)	MISSENSE
P65	LRP1B	c.11846A>C(p.K3949T	MISSENSE
P65	PALB2	c.1348A>C(p.N450H)	MISSENSE
P65	B2M	c.128T>G(p.L43R)	MISSENSE
P65	INPP4B	c.425G>A(p.R142Q)	MISSENSE
P65	SETD2	c.7351G>A(p.A2451T	MISSENSE
P65	ATR	c.5110G>T(p.E1704*)	STOP GAINED

P65	PMS2	c.1279C>T(p.R427C)	MISSENSE
P65	CASP8	c.1207T>G(p.S403A)	MISSENSE
P65	LHCGR	c.435C>A(p.F145L)	MISSENSE
P65	FLT3	c.866A>G(p.N289S)	MISSENSE
P65	ALK	c.700C>T(p.P234S)	MISSENSE
P65	GRM3	c.1472C>T(p.S491L)	MISSENSE
P65	FLT4	c.3199G>A(p.D1067N	MISSENSE
P65	ROS1	c.6614G>T(p.R2205I)	MISSENSE
P65	ESR1	c.754A>G(p.K252E)	MISSENSE
P65	APC	c.3514C>A(p.H1172N	MISSENSE
P65	LRP1B	c.8791T>G(p.L2931V)	MISSENSE
P65	EZH2	c.155G>T(p.R52I)	MISSENSE
P65	EZH2	c.143A>C(p.K48T)	MISSENSE
P65	KDM5A	c.2839G>A(p.E947K)	MISSENSE
P65	ABCB1	c.298G>T(p.D100Y)	MISSENSE
P65	ABCB1	c.1956A>C(p.E652D)	MISSENSE
P65	ATM	c.8386T>G(p.F2796V)	MISSENSE
P65	PAX5	c.316A>C(p.N106H)	MISSENSE
P65	FLT3	c.937C>A(p.L313M)	MISSENSE
P65	TP63	c.566C>T(p.S189L)	MISSENSE
P65	MPL	c.239G>A(p.S80N)	MISSENSE
P65	NRG1	c.302C>T(p.A101V)	MISSENSE
P65	LRP1B	c.10705A>C(p.K3569(	MISSENSE
P65	ABCB1	c.2861C>T(p.A954V)	MISSENSE
P65	ATR	c.5943A>C(p.E1981D)	MISSENSE
P65	DPYD	c.2951A>G(p.D984G)	MISSENSE
P65	MLH3	c.2108A>C(p.K703T)	MISSENSE
P65	GRIN2A	c.1822C>A(p.L608I)	MISSENSE
P65	RUNX1T1	c.1381C>T(p.R461C)	MISSENSE
P65	SETD2	c.3260C>A(p.S1087Y)	MISSENSE
P65	GRM8	c.364G>A(p.A122T)	MISSENSE
P65	ROS1	c.734G>T(p.R245I)	MISSENSE
P65	RAD51	c.223C>A(p.L75M)	MISSENSE
P65	NFE2L2	c.1285G>T(p.E429*)	STOP GAINED
P65	ROS1	c.3632A>G(p.N1211S	MISSENSE
P65	STK11	c.94A>G(p.T32A)	MISSENSE
P65	CBLB	c.2881G>A(p.E961K)	MISSENSE
P65	FANCD2	c.2351C>T(p.S784F)	MISSENSE
P65	FAT1	c.3907G>A(p.E1303K)	MISSENSE
P65	FGFR3	c.1209G>T(p.K403N)	MISSENSE
P65	NF1	c.3297A>C(p.K1099N)	MISSENSE
P65	FAT1	c.3191G>A(p.R1064K)	MISSENSE
P65	ARID2	c.919G>A(p.A307T)	MISSENSE
P65	PIK3CA	c.2373G>T(p.E791D)	MISSENSE

P65	SMAD2	c.1114A>G(p.T372A)	MISSENSE
P65	NBN	c.1367A>C(p.K456T)	MISSENSE
P65	HGF	c.1141C>A(p.P381T)	MISSENSE
P65	FAT1	c.2101G>A(p.D701N)	MISSENSE
P65	HDAC2	c.1002C>A(p.Y334*)	STOP GAINED
P65	PKHD1	c.4975G>T(p.E1659*)	STOP GAINED
P65	NF1	c.782A>C(p.K261T)	MISSENSE
P65	ERBB4	c.99G>T(p.E33D)	MISSENSE
P65	PTEN	c.1004G>A(p.R335Q)	MISSENSE
P65	PTPN13	c.3308T>C(p.F1103S)	MISSENSE
P65	PKHD1	c.12147G>T(p.E4049D	MISSENSE
P65	NTRK3	c.363A>C(p.R121S)	MISSENSE
P65	FANCL	c.220A>G(p.M74V)	MISSENSE
P65	ERBB3	c.2226A>C(p.K742N)	MISSENSE
P65	FGFR1	c.1072G>A(p.V358I)	MISSENSE
P65	KDR	c.1812G>T(p.K604N)	MISSENSE
P65	RICTOR	c.974G>A(p.R325Q)	MISSENSE
P65	ROS1	c.6436T>C(p.S2146P)	MISSENSE
P65	ATR	c.2905G>T(p.E969*)	STOP GAINED
P65	BRIP1	c.493G>T(p.E165*)	STOP GAINED
P65	APC	c.4463T>G(p.L1488*)	STOP GAINED
P65	ARID1B	c.6305T>G(p.L2102*)	STOP GAINED
P65	PDGFRA	c.2310G>T(p.K770N)	MISSENSE
P65	CEP57	c.614T>G(p.L205R)	MISSENSE
P65	DAXX	c.423G>T(p.K141N)	MISSENSE
P65	TTF1	c.1834G>A(p.D612N)	MISSENSE
P65	IGF1R	c.2056A>G(p.N686D)	MISSENSE
P65	CDK8	c.518A>G(p.D173G)	MISSENSE
P65	ATM	c.3646T>G(p.Y1216D)	MISSENSE
P65	BAP1	c.2182C>T(p.R728C)	MISSENSE
P65	CSF1R	c.881G>A(p.R294Q)	MISSENSE
P66	FANCL	c.925A>G(p.I309V)	MISSENSE
P67	SMAD4	c.1477G>A(p.D493N)	MISSENSE
P67	ATR	c.4037C>T(p.A1346V)	MISSENSE
P67	PIK3CA	c.1028A>T(p.Y343F)	MISSENSE
P67	SMAD4	c.1082G>A(p.R361H)	MISSENSE
P67	AMER1	c.1358T>G(p.L453R)	MISSENSE
P67	GNAS	c.683G>A(p.R228H)	MISSENSE
P67	KRAS	c.35G>A(p.G12D)	MISSENSE
P68	AXL	c.277C>A(p.Q93K)	MISSENSE
P68	APC	c.4012C>T(p.Q1338*)	STOP GAINED
P68	TP53	c.742C>T(p.R248W)	MISSENSE
P68	APC	c.1495C>T(p.R499*)	STOP GAINED
P68	LRP1B	c.3810C>G(p.I1270M)	MISSENSE

P69	CYP2D6	c.875A>T(p.D292V)	MISSENSE
P69	LHCGR	c.287del(p.N96Tfs*9)	FRAMESHIFT
P69	PTEN	c.741dup(p.P248Tfs*!	FRAMESHIFT
P69	ARID2	c.214del(p.I72Lfs*18)	FRAMESHIFT
P69	SETBP1	c.3983A>G(p.D1328G	MISSENSE
P69	ATM	c.4416_4423dup(p.Y1	STOP GAINED
P69	PIK3CA	c.1633G>A(p.E545K)	MISSENSE
P69	SMAD3	c.707C>T(p.S236F)	MISSENSE
P69	KRAS	c.35G>C(p.G12A)	MISSENSE
P69	APC	c.4484del(p.S1495Ifs'	FRAMESHIFT
P69	KRAS	c.436G>A(p.A146T)	MISSENSE
P70	LRP1B	c.8897G>A(p.C2966Y)	MISSENSE
P70	TP53	c.488A>G(p.Y163C)	MISSENSE
P70	PPARD	c.295C>A(p.R99S)	MISSENSE
P70	KRAS	c.35G>A(p.G12D)	MISSENSE
P70	GATA4	c.307C>T(p.P103S)	MISSENSE
P70	APC	c.4350del(p.E1451Kfs	FRAMESHIFT
P70	TOP2A	c.787C>T(p.P263S)	MISSENSE
P70	APC	c.2626C>T(p.R876*)	STOP GAINED
P70	PTEN	c.509G>T(p.S170I)	MISSENSE
P71	ALK	c.3257C>T(p.S1086L)	MISSENSE
P71	SMARCA4	c.701C>T(p.P234L)	MISSENSE
P71	SETBP1	c.3233C>T(p.T1078M	MISSENSE
P71	PTEN	c.634+1G>A	SPLICE
P71	TP53	c.503A>G(p.H168R)	MISSENSE
P71	KRAS	c.35G>T(p.G12V)	MISSENSE
P71	APC	c.3441_3492del(p.Y1:	FRAMESHIFT
P71	MITF	c.1093G>A(p.A365T)	MISSENSE
P71	CHD4	c.1169G>A(p.R390H)	MISSENSE
P71	GATA4	c.88G>A(p.A30T)	MISSENSE
P71	ATR	c.2800dup(p.C934Lfs'	FRAMESHIFT
P71	PREX2	c.1211C>G(p.T404S)	MISSENSE
P72	APC	c.1370C>A(p.S457*)	STOP GAINED
P72	APC	c.4285C>T(p.Q1429*)	STOP GAINED
P72	QKI	c.725G>A(p.R242H)	MISSENSE
P72	TP53	c.376-1G>A	SPLICE
P72	PIK3CA	c.1633G>A(p.E545K)	MISSENSE
P72	AXIN2	c.74C>T(p.P25L)	MISSENSE
P72	KRAS	c.38G>A(p.G13D)	MISSENSE
P72	FAT1	c.8011_8012del(p.F2(	FRAMESHIFT
P72	TP53		Deletion
P73	SMAD4		Deletion
P73	APC	c.1234C>T(p.Q412*)	STOP GAINED
P73	ETV1	c.605C>T(p.T202M)	MISSENSE

P73	RUNX1	c.731G>A(p.R244K)	MISSENSE
P73	APC		Deletion
P73	KRAS	c.436G>A(p.A146T)	MISSENSE
P73	TP53	c.824G>A(p.C275Y)	MISSENSE
P74	APC	c.4348C>T(p.R1450*)	STOP GAINED
P74	TP53	c.286del(p.S96Lfs*27	FRAMESHIFT
P75	CDKN2B	c.302G>A(p.R101Q)	MISSENSE
P75	ERCC2	c.1609G>A(p.A537T)	MISSENSE
P75	MSH2	c.1759+2del	SPLICE
P75	EMSY	c.1474G>A(p.G492S)	MISSENSE
P75	RET	c.2030G>A(p.R677Q)	MISSENSE
P75	MED12	c.2905C>T(p.R969C)	MISSENSE
P75	PLK1	c.1294G>A(p.V432M)	MISSENSE
P75	ERCC2	c.1349G>A(p.R450H)	MISSENSE
P75	MEF2B	c.819del(p.T274Pfs*1	FRAMESHIFT
P75	FBXW7	c.1647del(p.S550Vfs*	FRAMESHIFT
P75	PTPN13	c.3006del(p.K1002Nfs	FRAMESHIFT
P75	ATM	c.5690del(p.F1897Sfs	FRAMESHIFT
P75	RECQL4	c.3139_3141del(p.E1(	INFRAME INDEL
P75	ARID1B	c.2342C>T(p.A781V)	MISSENSE
P75	NOTCH1	c.5272C>T(p.R1758C)	MISSENSE
P75	GNAS	c.602G>A(p.R201H)	MISSENSE
P75	RNF43	c.349C>A(p.R117S)	MISSENSE
P75	MGMT	c.488C>T(p.A163V)	MISSENSE
P75	RNF43	c.350del(p.R117Pfs*4	FRAMESHIFT
P75	LRP1B	c.9484G>A(p.G3162R	MISSENSE
P75	ERBB3	c.2999T>C(p.L1000P)	MISSENSE
P75	MPL	c.509G>A(p.R170H)	MISSENSE
P75	GNAS	c.1535G>A(p.R512Q)	MISSENSE
P75	CTCF	c.1697G>A(p.R566H)	MISSENSE
P75	DENND1A	c.220G>A(p.V74M)	MISSENSE
P75	B2M	c.285del(p.D96Mfs*7	FRAMESHIFT
P75	MSH2	c.1609A>T(p.K537*)	STOP GAINED
P75	RNF43	c.394C>T(p.R132*)	STOP GAINED
P75	BLM	c.3530C>T(p.A1177V)	MISSENSE
P75	IGF2	c.298G>A(p.V100M)	MISSENSE
P75	AXIN2	c.2078C>T(p.T693M)	MISSENSE
P75	ARID2	c.3880_3881del(p.L1	FRAMESHIFT
P75	NTRK2	c.1174T>C(p.Y392H)	MISSENSE
P75	PLCB4	c.3307C>T(p.R1103W	MISSENSE
P75	ARAF	c.571del(p.R191Afs*1	FRAMESHIFT
P75	NOTCH2	c.5996del(p.N1999M	FRAMESHIFT
P75	AXIN2	c.1994dup(p.N666Qfs	FRAMESHIFT
P75	CDK12	c.4066C>T(p.R1356*)	STOP GAINED

P75	B2M	c.346+1dup	SPLICE
P75	TSC1	c.3130G>A(p.E1044K)	MISSENSE
P75	SMARCA4	c.3727C>T(p.R1243W	MISSENSE
P75	PKHD1	c.5396T>C(p.L1799P)	MISSENSE
P75	SMARCA4	c.4328G>A(p.R1443H	MISSENSE
P75	TNFRSF11	c.401C>T(p.A134V)	MISSENSE
P75	FLT3	c.2128T>A(p.F710I)	MISSENSE
P75	ARID2	c.1277C>T(p.T426M)	MISSENSE
P75	SETBP1	c.3961C>T(p.R1321C)	MISSENSE
P76	KRAS	c.35G>T(p.G12V)	MISSENSE
P76	ATM	c.6679C>T(p.R2227C)	MISSENSE
P76	APC	c.4285C>T(p.Q1429*)	STOP GAINED
P76	ATRX	c.6647A>G(p.D2216G	MISSENSE
P76	APC	c.2626C>T(p.R876*)	STOP GAINED
P76	MRE11	c.1618T>G(p.F540V)	MISSENSE
P77	APC	c.4188del(p.F1396Lfs	FRAMESHIFT
P77	TP53	c.318C>G(p.S106R)	MISSENSE
P77	PARP1	c.1076del(p.P359Qfs*	FRAMESHIFT
P77	ASCL4	c.124C>A(p.R42S)	MISSENSE
P78	APC	c.3929dup(p.I1311Df	FRAMESHIFT
P78	FBXW7	c.1040G>A(p.R347H)	MISSENSE
P78	FBXW7	c.1391C>T(p.S464L)	MISSENSE
P78	JARID2	c.1817G>A(p.R606Q)	MISSENSE
P78	TP53	c.821_826dup(p.V27	INFRAME INDEL
P78	KRAS	c.34G>T(p.G12C)	MISSENSE
P79	FBXW7	c.1040G>A(p.R347H)	MISSENSE
P79	TP53	c.689C>A(p.T230N)	MISSENSE
P79	ERBB3	c.179T>G(p.M60R)	MISSENSE
P79	BCR	c.2842C>G(p.R948G)	MISSENSE
P79	TGFBR2	c.458dup(p.P154Afs*	FRAMESHIFT
P79	KMT2B	c.3550G>A(p.V1184M	MISSENSE
P79	NRG1	c.1136C>G(p.S379*)	STOP GAINED
P79	SOCS1	c.313G>A(p.D105N)	MISSENSE
P79	TGFBR2	c.1394A>G(p.E465G)	MISSENSE
P79	ERBB2	c.929C>T(p.S310F)	MISSENSE
P79	KRAS	c.35G>A(p.G12D)	MISSENSE
P79	SETD2	c.4053C>G(p.D1351E	MISSENSE
P79	ROS1	c.3515A>G(p.N1172S	MISSENSE
P79	CHD4	c.3314G>A(p.R1105Q	MISSENSE
P79	MED12	c.47G>A(p.R16Q)	MISSENSE
P79	MTOR	c.1363G>A(p.V455M)	MISSENSE
P79	TP53	c.693del(p.I232Sfs*1	FRAMESHIFT
P80	PIK3R1	c.1946G>A(p.R649Q)	MISSENSE
P80	SGK1	c.440G>T(p.R147L)	MISSENSE

P80	FANCE	c.1088T>C(p.L363P)	MISSENSE
P80	AMER1	c.1072C>T(p.R358*)	STOP GAINED
P80	LRP1B	c.4576C>T(p.Q1526*)	STOP GAINED
P80	ATR	c.1367T>C(p.M456T)	MISSENSE
P80	LRP1B	c.884G>A(p.R295Q)	MISSENSE
P80	ERCC4	c.2005A>G(p.T669A)	MISSENSE
P80	WRN	c.3143G>T(p.R1048I)	MISSENSE
P80	IFNA6	c.268C>A(p.L90I)	MISSENSE
P80	PTCH1	c.3782A>C(p.N1261T)	MISSENSE
P80	STAG2	c.1841G>A(p.R614Q)	MISSENSE
P80	DOT1L	c.1364C>T(p.S455F)	MISSENSE
P80	PTEN	c.451G>C(p.A151P)	MISSENSE
P80	PTCH1	c.3397A>G(p.T1133A)	MISSENSE
P80	POLH	c.73C>T(p.Q25*)	STOP GAINED
P80	PREX2	c.1752A>C(p.K584N)	MISSENSE
P80	BRCA1	c.1982G>T(p.R661M)	MISSENSE
P80	DAXX	c.554G>A(p.R185Q)	MISSENSE
P80	FLCN	c.256C>T(p.R86W)	MISSENSE
P80	PIK3C3	c.349G>A(p.G117R)	MISSENSE
P80	PLK1	c.1498C>T(p.R500C)	MISSENSE
P80	IL7R	c.1181G>A(p.G394D)	MISSENSE
P80	PRDM1	c.223A>G(p.T75A)	MISSENSE
P80	MSH6	c.14G>A(p.S5N)	MISSENSE
P80	GRIN2A	c.4127G>A(p.R1376H	MISSENSE
P80	JARID2	c.1879C>T(p.R627W)	MISSENSE
P80	CHD8	c.5390G>T(p.R1797L)	MISSENSE
P80	NF1	c.3496G>T(p.G1166C)	MISSENSE
P80	RECQL4	c.2507A>G(p.Q836R)	MISSENSE
P80	TSHR	c.1188G>T(p.M396I)	MISSENSE
P80	PALB2	c.1988G>A(p.R663H)	MISSENSE
P80	NTRK1	c.1040G>A(p.R347H)	MISSENSE
P80	CUL3	c.1193delA(p.K398Rfs	FRAMESHIFT
P80	AXL	c.874dupC(p.H292Pfs	FRAMESHIFT
P80	KMT2B	c.521dupC(p.T176Dfs	FRAMESHIFT
P80	PIK3R1	c.244dupA(p.I82Nfs*[illegible]	FRAMESHIFT
P80	NOTCH2	c.3415delC(p.L1139W	FRAMESHIFT
P80	TAP2	c.223delC(p.L75*)	FRAMESHIFT
P80	FAT1	c.12041C>T(p.T4014N	MISSENSE
P80	IDH1	c.293C>T(p.T98I)	MISSENSE
P80	TSC2	c.229G>A(p.A77T)	MISSENSE
P80	IFNGR1	c.1132_1133delAG(p.	FRAMESHIFT
P80	PTEN	c.800delA(p.K267Rfs*	FRAMESHIFT
P80	BRIP1	c.3140delT(p.L1047Cf	FRAMESHIFT
P80	FBXW7	c.1941delA(p.K647Nf	FRAMESHIFT

P80	PBRM1	c.773delA(p.N258Mfs	FRAMESHIFT
P80	TNFAIP3	c.1445G>A(p.G482D)	MISSENSE
P80	SMARCA4	c.4303G>A(p.D1435N	MISSENSE
P80	IFNGR1	c.253T>C(p.C85R)	MISSENSE
P80	MSH6	c.3260_3261dupCC(p	FRAMESHIFT
P80	CUL3	c.1358dupA(p.N453K	FRAMESHIFT
P80	TAP2	c.552dupT(p.D185*)	FRAMESHIFT
P80	APC	c.4734T>A(p.C1578*)	STOP GAINED
P80	TEK	c.3368C>T(p.A1123V)	MISSENSE
P80	KMT2B	c.5501G>A(p.R1834H	MISSENSE
P80	KMT2A	c.5114C>A(p.P1705H)	MISSENSE
P80	CDH1	c.2515G>A(p.G839S)	MISSENSE
P80	TGFBR2	c.182G>A(p.C61Y)	MISSENSE
P80	MUTYH	c.1063G>A(p.V355M)	MISSENSE
P80	KMT2B	c.4076A>C(p.Q1359P	MISSENSE
P80	TOP2A	c.3613delA(p.T1205H	FRAMESHIFT
P80	MLH3	c.2021delA(p.N674Ifs	FRAMESHIFT
P80	QKI	c.401delA(p.K134Rfs*	FRAMESHIFT
P80	PREX2	c.1007delA(p.N336Ifs	FRAMESHIFT
P80	FLT1	c.1695_1696delAA(p.	FRAMESHIFT
P80	APC	c.1690C>T(p.R564*)	STOP GAINED
P80	BAP1	c.869A>G(p.N290S)	MISSENSE
P80	CHD8	c.2828G>A(p.R943H)	MISSENSE
P80	MAP2K1	c.602G>A(p.R201H)	MISSENSE
P80	TP53	c.848G>A(p.R283H)	MISSENSE
P80	SMARCA4	c.2900G>A(p.R967H)	MISSENSE
P80	GATA3	c.256C>T(p.R86C)	MISSENSE
P80	C11orf30	c.926C>T(p.T309M)	MISSENSE
P80	PMS2	c.947A>G(p.H316R)	MISSENSE
P80	FANCM	c.4005delA(p.V1336L	FRAMESHIFT
P81	TP53	c.743G>A(p.R248Q)	MISSENSE
P81	LRP1B	c.7432T>G(p.L2478V)	MISSENSE
P81	QKI	c.401dupA(p.E135Gfs	FRAMESHIFT
P81	KMT2B	c.5563C>T(p.P1855S)	MISSENSE
P81	POLD1	c.2677G>A(p.D893N)	MISSENSE
P81	EIF1AX	c.38G>A(p.R13H)	MISSENSE
P81	EPHA3	c.236A>G(p.N79S)	MISSENSE
P81	NF1		Deletion
P81	SMAD4	c.1082G>A(p.R361H)	MISSENSE
P81	BRCA2	c.4316C>T(p.A1439V)	MISSENSE
P81	ARID1A	c.6763G>A(p.E2255K)	MISSENSE
P81	APC	c.694C>T(p.R232*)	STOP GAINED
P81	RNF43	c.198A>T(p.E66D)	MISSENSE
P82	KRAS	c.35G>A(p.G12D)	MISSENSE

P82	PBRM1	c.3425A>G(p.K1142R)	MISSENSE
P82	IDH1	c.394C>T(p.R132C)	MISSENSE
P82	GATA6	IGR (upstream RBBP8	FUSION
P82	MDM2	c.1026A>C(p.K342N)	MISSENSE
P82	NF1	c.7039A>G(p.S2347G)	MISSENSE
P82	TP53	c.524G>A(p.R175H)	MISSENSE
P82	APC	c.694C>T(p.R232*)	STOP GAINED
P82	KRAS	c.35G>T(p.G12V)	MISSENSE
P82	PKHD1	c.5134G>A(p.G1712R	MISSENSE
P82	PMS1	c.784A>T(p.I262L)	MISSENSE
P82	TP53	c.1024delC(p.R342Efs	FRAMESHIFT
P83	PTEN		Deletion
P83	GRM8	c.2372G>T(p.C791F)	MISSENSE
P83	APC	c.2821G>T(p.E941*)	STOP GAINED
P83	TP53	c.916C>T(p.R306*)	STOP GAINED
P83	SMO	c.734C>T(p.T245M)	MISSENSE
P83	KRAS	c.35G>A(p.G12D)	MISSENSE
P83	APC	c.4473delT(p.F1491Lf	FRAMESHIFT
P83	FBXW7	c.1436G>A(p.R479Q)	MISSENSE
P83	CHEK2	OSBP2:exon1~CHEK2	FUSION
P83	EPHA5	c.832G>A(p.A278T)	MISSENSE
P83	APC	c.4475C>G(p.A1492G	MISSENSE
P84	PALB2	c.3263C>T(p.P1088L)	MISSENSE
P84	AXL	c.2260C>G(p.R754G)	MISSENSE
P84	TP53	c.487T>C(p.Y163H)	MISSENSE
P84	APC	c.694C>T(p.R232*)	STOP GAINED
P84	APC	c.3927_3931delAAAG	FRAMESHIFT
P84	KRAS	c.38G>A(p.G13D)	MISSENSE
P85	PLCB4	c.2246A>G(p.N749S)	MISSENSE
P85	POLH	c.767G>A(p.R256H)	MISSENSE
P85	APC	c.694C>T(p.R232*)	STOP GAINED
P85	DOT1L	c.3341C>T(p.A1114V)	MISSENSE
P85	PGR	c.1259G>T(p.G420V)	MISSENSE
P85	TP53	c.746G>T(p.R249M)	MISSENSE
P86	TP53	c.743G>A(p.R248Q)	MISSENSE
P86	CHD4	c.3203G>A(p.R1068H	MISSENSE
P86	FBXW7	c.1099C>T(p.R367*)	STOP GAINED
P86	CHD8	c.5009C>T(p.A1670V)	MISSENSE
P86	GRIN2A	c.2984C>T(p.T995M)	MISSENSE
P86	BRCA2	c.2483A>G(p.Y828C)	MISSENSE
P86	APC	c.4132C>T(p.Q1378*)	STOP GAINED
P86	APC	c.1431dupA(p.L478Ifs	FRAMESHIFT
P87	NOTCH1	c.3271G>A(p.G1091S)	MISSENSE
P87	TP53	c.743G>A(p.R248Q)	MISSENSE

P87	KRAS	c.34G>T(p.G12C)	MISSENSE
P88	TP53	c.817C>T(p.R273C)	MISSENSE
P89	APC	c.4461_4476delTTTA	FRAMESHIFT
P89	TP53	c.524G>A(p.R175H)	MISSENSE
P89	TP53	c.524G>A(p.R175H)	MISSENSE
P89	APC	c.2626C>T(p.R876*)	STOP GAINED
P89	NRAS	c.181C>A(p.Q61K)	MISSENSE
P90	ZNF703		Amplication
P90	PRKDC	c.3395A>G(p.D1132G	MISSENSE
P90	APC	c.4189G>T(p.E1397*)	STOP GAINED
P90	DPYD	c.2452_2457dupGCC	INFRAME INDEL
P90	ERCC5	c.1016C>A(p.P339H)	MISSENSE
P90	TP53	c.637C>T(p.R213*)	STOP GAINED
P90	APC	c.2626C>T(p.R876*)	STOP GAINED
P91	FBXW7	c.988dupA(p.I330Nfs	FRAMESHIFT
P91	SMAD4	c.346C>T(p.Q116*)	STOP GAINED
P91	MTOR	c.6040G>A(p.E2014K	MISSENSE
P91	TP53	c.916C>T(p.R306*)	STOP GAINED
P91	KRAS	c.35G>T(p.G12V)	MISSENSE
P91	APC	c.1312+2T>C	SPLICE
P91	PARP2	c.1087A>G(p.R363G)	MISSENSE
P91	ATRX	c.3397G>T(p.E1133*)	STOP GAINED
P91	CXCR4	c.884G>C(p.C295S)	MISSENSE
P92	POLE	c.125A>G(p.D42G)	MISSENSE
P92	RAD51D	c.145G>A(p.A49T)	MISSENSE
P92	NBN	c.1397G>A(p.R466K)	MISSENSE
P92	RNF43	c.349C>A(p.R117S)	MISSENSE
P92	SF3B1	c.581C>A(p.S194*)	STOP GAINED
P92	POLE	c.204+2T>C	SPLICE
P92	GATA4	c.1232C>T(p.A411V)	MISSENSE
P92	FAT1	c.13009G>A(p.V4337	MISSENSE
P92	UGT1A1	c.353dupA(p.D119Gf	FRAMESHIFT
P92	EGFR	c.3631T>C(p.*1211R	STOP LOST
P92	MAP2K2	c.553C>T(p.R185*)	STOP GAINED
P92	BRCA2	c.44T>C(p.F15S)	MISSENSE
P92	MAP2K2	c.1064C>T(p.A355V)	MISSENSE
P92	APC	c.3875C>T(p.T1292M	MISSENSE
P92	ZNF217	c.2461C>T(p.H821Y)	MISSENSE
P92	IDH1	c.766G>A(p.A256T)	MISSENSE
P92	GATA4	c.776G>A(p.R259H)	MISSENSE
P92	RICTOR	c.4610T>C(p.L1537P)	MISSENSE
P92	KMT2A	c.11455C>T(p.R3819	MISSENSE
P92	FANCA	c.1661C>T(p.A554V)	MISSENSE
P92	ERCC1	c.659C>T(p.A220V)	MISSENSE

P92	ALK	c.3707G>A(p.G1236D	MISSENSE
P92	VHL	c.613C>T(p.R205C)	MISSENSE
P92	PIK3CA	c.328_330delGAA(p.E	INFRAME INDEL
P92	MYCN	c.302dupG(p.L102Tfs	FRAMESHIFT
P92	POLD1	c.2959delG(p.D987Tf:	SPLICE
P92	GRM3	c.2314G>T(p.G772C)	MISSENSE
P92	CDKN2B	c.371G>A(p.G124D)	MISSENSE
P92	KDM5A	c.3597delA(p.G1200D	FRAMESHIFT
P92	ARID1A	c.4555delC(p.Q1519R	FRAMESHIFT
P92	LZTR1	c.731C>A(p.S244Y)	MISSENSE
P92	FGFR4	c.253C>T(p.R85C)	MISSENSE
P92	BRCA1	c.1016delA(p.K339Rfs	FRAMESHIFT
P92	CHD4	c.218dupA(p.E74Gfs*	FRAMESHIFT
P92	FLCN	c.1285delC(p.H429Tfs	FRAMESHIFT
P92	SPOP	c.1100delC(p.P367Hfs	FRAMESHIFT
P92	ATM	c.7174C>T(p.R2392W	MISSENSE
P92	MTOR	c.5246G>A(p.R1749Q	MISSENSE
P92	QKI	c.401delA(p.K134Rfs*	FRAMESHIFT
P92	AMER1	c.1832G>A(p.R611K)	MISSENSE
P92	SMARCB1	c.641C>T(p.T214M)	MISSENSE
P92	SMARCA4	c.597C>A(p.H199Q)	MISSENSE
P92	HGF	c.85G>A(p.A29T)	MISSENSE
P92	DDR2	c.1552delG(p.V518Cfs	FRAMESHIFT
P92	RAD50	c.2165dupA(p.E723G	FRAMESHIFT
P92	NOTCH1	c.2605G>A(p.D869N)	MISSENSE
P92	PALB2	c.1249T>C(p.S417P)	MISSENSE
P92	ERBB4	c.2681A>G(p.Y894C)	MISSENSE
P92	RPTOR	c.3770C>T(p.T1257M	MISSENSE
P92	NF1	c.4247C>A(p.P1416H)	MISSENSE
P92	BARD1	c.1126T>C(p.S376P)	MISSENSE
P92	NOTCH2	c.1264G>T(p.A422S)	MISSENSE
P92	PLCB4	c.2429C>T(p.S810L)	MISSENSE
P92	JARID2	c.1272delG(p.R425Gf	FRAMESHIFT
P92	JAK2	c.1680delT(p.F560Lfs	FRAMESHIFT
P92	GNAS	c.794G>A(p.R265H)	MISSENSE
P92	BRCA2	c.7505G>A(p.R2502H	MISSENSE
P92	DENND1A	c.1268C>T(p.A423V)	MISSENSE
P92	GRIN2A	c.3505C>T(p.R1169W	MISSENSE
P92	CHD8	c.3338G>A(p.R1113H	MISSENSE
P92	BRD4	c.1001A>G(p.D334G)	MISSENSE
P92	EPHA2	c.719delG(p.G240Vfs	FRAMESHIFT
P92	SPRED1	c.234delA(p.D79Tfs*4	FRAMESHIFT
P92	YAP1	c.147delC(p.A50Pfs*2	FRAMESHIFT
P92	PALB2	c.3256C>T(p.R1086*)	STOP GAINED

P92	KRAS	c.35G>A(p.G12D)	MISSENSE
P92	GNAS	c.1882C>T(p.P628S)	MISSENSE
P92	TNFAIP3	c.296G>A(p.G99D)	MISSENSE
P92	JAK2	c.3338G>A(p.R1113H	MISSENSE
P92	RNF43	c.350delG(p.R117Pfs*	FRAMESHIFT
P92	TTF1	c.821delA(p.K274Sfs*	FRAMESHIFT
P92	PREX2	c.1693delT(p.S565Rfs	FRAMESHIFT
P92	GNAS	c.767C>T(p.A256V)	MISSENSE
P92	CHD8	c.7721A>T(p.D2574V)	MISSENSE
P92	MYCN	c.613G>A(p.V205M)	MISSENSE
P92	APC	c.4715T>A(p.I1572N)	MISSENSE
P92	KMT2A	c.92dupC(p.R32Afs*1	FRAMESHIFT
P92	BAP1	c.1259G>A(p.G420E)	MISSENSE
P92	FAT1	c.11597C>T(p.A3866V	MISSENSE
P92	RUNX1	c.1319C>T(p.A440V)	MISSENSE
P92	CHD4	c.5270G>A(p.R1757H	MISSENSE
P92	ERBB3	c.2000G>A(p.R667H)	MISSENSE
P92	BAX	c.121delG(p.E41Rfs*1	FRAMESHIFT
P92	SMO	c.1202C>T(p.A401V)	MISSENSE
P92	IFNGR1	c.486T>A(p.D162E)	MISSENSE
P92	FAT1	c.8799delA(p.G2934V	FRAMESHIFT
P92	PARP1	c.628G>T(p.G210C)	MISSENSE
P92	POT1	c.260A>G(p.Q87R)	MISSENSE
P92	PGR	c.1223G>A(p.G408D)	MISSENSE
P92	EXT2	c.1771T>C(p.S591P)	MISSENSE
P92	BTK	c.216T>A(p.N72K)	MISSENSE
P92	LRP1B	c.8528G>A(p.R2843Q	MISSENSE
P92	MSH6	c.741delA(p.K247Nfs*	FRAMESHIFT
P92	BLM	c.2268dupA(p.D757R	FRAMESHIFT
P92	SETBP1	c.440delA(p.N147Ifs*	FRAMESHIFT
P92	KIT	c.1015G>A(p.A339T)	MISSENSE
P92	EPHA5	c.1151G>A(p.G384D)	MISSENSE
P92	RNF43	c.65dupA(p.A23Gfs*1	FRAMESHIFT
P92	KMT2A	c.126delC(p.P45Rfs*1	FRAMESHIFT
P92	PRKCI	c.826delA(p.T276Qfs*	FRAMESHIFT
P92	TSC1	c.2931dupA(p.L978Tf	FRAMESHIFT
P92	EPHA2	c.1379dupC(p.P461Af	FRAMESHIFT
P92	PBRM1	c.4484A>G(p.Q1495R	MISSENSE
P92	FBXW7	c.1513C>T(p.R505C)	MISSENSE
P92	EP300	c.1511G>A(p.R504Q)	MISSENSE
P92	KMT2B	c.5006C>T(p.A1669V)	MISSENSE
P92	RICTOR	c.436G>T(p.A146S)	MISSENSE
P92	ATR	c.3799G>A(p.V1267I)	MISSENSE
P92	NTRK2	c.2375G>A(p.R792H)	MISSENSE

P92	NTRK2	c.1816G>A(p.E606K)	MISSENSE
P92	GRM3	c.755T>C(p.I252T)	MISSENSE
P92	FANCI	c.686T>C(p.V229A)	MISSENSE
P92	THADA	c.5147A>G(p.N1716S	MISSENSE
P92	AKT1	c.320C>T(p.A107V)	MISSENSE
P93	FLT3		Amplication
P93	KRAS	c.38G>A(p.G13D)	MISSENSE
P93	APC	c.1147dupG(p.A383G	FRAMESHIFT
P93	FAT1	c.4653delT(p.N1551K	FRAMESHIFT
P93	TP53	c.335_338dupGCTT(p	FRAMESHIFT
P93	LRP1B	c.4520A>G(p.Q1507R	MISSENSE
P93	YAP1	c.229A>G(p.T77A)	MISSENSE
P93	PBRM1	c.4565A>G(p.Q1522R	MISSENSE
P93	APC	c.2413C>T(p.R805*)	STOP GAINED
P93	FLT1		Amplication
P93	BAI3	c.3978C>G(p.S1326R)	MISSENSE
P93	SMAD4		Deletion
P93	APC	c.4737delT(p.I1580Ff	FRAMESHIFT
P94	ERBB2	c.2033G>A(p.R678Q)	MISSENSE
P94	GNAS	c.1532G>A(p.R511H)	MISSENSE
P95	TP53	c.586C>T(p.R196*)	STOP GAINED
P95	CD274	c.374G>A(p.R125Q)	MISSENSE
P95	AKT1	c.49G>A(p.E17K)	MISSENSE
P95	KRAS	c.38G>A(p.G13D)	MISSENSE
P95	SMAD4	c.1082G>A(p.R361H)	MISSENSE
P95	SETD2	c.2489A>T(p.E830V)	MISSENSE
P96	FANCA	c.4285G>A(p.D1429N	MISSENSE
P96	PIK3CA	c.1345C>A(p.P449T)	MISSENSE
P96	EPHA3	c.1895T>G(p.F632C)	MISSENSE
P96	TP53	c.770T>G(p.L257R)	MISSENSE
P96	KRAS	c.35G>A(p.G12D)	MISSENSE
P96	APC	c.1234C>T(p.Q412*)	STOP GAINED
P96	ERBB3	c.3701C>T(p.S1234F)	MISSENSE
P96	KMT2C	c.2742T>A(p.S914R)	MISSENSE
P97	CTNNB1	c.770C>T(p.T257I)	MISSENSE
P97	SMAD4	c.1521A>T(p.K507N)	MISSENSE
P97	PKHD1	c.8011C>T(p.R2671*)	STOP GAINED
P97	STAT3	c.2147C>T(p.T716M)	MISSENSE
P97	KRAS	c.35G>A(p.G12D)	MISSENSE
P97	NRAS	c.35G>A(p.G12D)	MISSENSE
P98	TP53	c.817C>T(p.R273C)	MISSENSE
P98	PREX2	c.3245G>A(p.S1082N	MISSENSE
P98	RNF43	c.1A>T(p.M1?)	START LOST
P98	LZTR1	c.27dupG(p.Q10Afs*2	FRAMESHIFT

P98	ESR1	c.296C>T(p.P99L)	MISSENSE
P98	NF1	c.130_131dupAT(p.N	FRAMESHIFT
P99	KMT2C	c.1149G>A(p.W383*)	STOP GAINED
P99	KRAS	c.40G>A(p.V14I)	MISSENSE
P99	B2M	c.43_44delCT(p.L15Ff	FRAMESHIFT
P99	TNFAIP3	c.365T>C(p.L122P)	MISSENSE
P99	PTEN	c.700C>T(p.R234W)	MISSENSE
P99	ERCC5	c.1372G>A(p.V458I)	MISSENSE
P99	FAT1	c.9683delC(p.P3228Lf	FRAMESHIFT
P99	CTNNB1	c.134C>T(p.S45F)	MISSENSE
P99	PTCH1	c.3490G>A(p.V1164I)	MISSENSE
P99	GRIN2A	c.2840G>T(p.R947M)	MISSENSE
P99	LZTR1	c.17G>A(p.S6N)	MISSENSE
P99	POLE	c.5024G>A(p.R1675H	MISSENSE
P99	PARK2	c.621A>T(p.E207D)	MISSENSE
P99	ARID1A	c.4030dupT(p.S1344F	FRAMESHIFT
P99	FLT1	c.2144C>T(p.T715M)	MISSENSE
P99	PLK1	c.661G>A(p.E221K)	MISSENSE
P99	CYLD	c.115C>A(p.L39I)	MISSENSE
P99	MTOR	c.406G>A(p.A136T)	MISSENSE
P99	NOTCH2	c.6862C>T(p.P2288S)	MISSENSE
P99	RET	c.2789C>T(p.T930M)	MISSENSE
P99	CDKN2A	c.386A>G(p.Y129C)	MISSENSE
P99	CREBBP	c.7150delC(p.H2384T	FRAMESHIFT
P99	CHD8	c.7112dupA(p.N2371	FRAMESHIFT
P99	ARID1B	c.4913G>A(p.R1638H	MISSENSE
P99	QKI	c.401delA(p.K134Rfs*	FRAMESHIFT
P99	BRD4	c.2006G>A(p.R669H)	MISSENSE
P99	NOTCH1	c.3511-1G>A	SPLICE
P99	IKBKE	c.560G>A(p.R187Q)	MISSENSE
P99	TSHR	c.50G>T(p.R17M)	MISSENSE
P99	CTCF	c.610dupA(p.T204Nfs	FRAMESHIFT
P99	LRP1B	c.7834delC(p.Q2612R	FRAMESHIFT
P99	ERBB2	c.3017G>A(p.R1006H	MISSENSE
P99	MLLT4	c.164_165delTT(p.F5	FRAMESHIFT
P99	BLM	c.1544delA(p.N515M	FRAMESHIFT
P99	RAD50	c.2801delA(p.N934Ifs	FRAMESHIFT
P99	FAT1	c.5854G>A(p.V1952I)	MISSENSE
P99	MLH1	c.1331dupA(p.N444K	FRAMESHIFT
P99	ERBB2	c.877G>A(p.A293T)	MISSENSE
P99	GATA4	c.572G>T(p.S191I)	MISSENSE
P99	MTOR	c.5819C>G(p.P1940R)	MISSENSE
P99	BRCA2	c.5200G>A(p.E1734K)	MISSENSE
P99	PIK3CA	c.323G>A(p.R108H)	MISSENSE

P99	BAP1	c.1702G>T(p.G568W)	MISSENSE
P99	CYSLTR2	c.442C>T(p.H148Y)	MISSENSE
P99	FLCN	c.1285delC(p.H429Tfs	FRAMESHIFT
P99	PARK2	c.1310C>T(p.P437L)	MISSENSE
P99	PKHD1	c.10487T>C(p.L3496S	MISSENSE
P99	ARID1A	c.736G>A(p.A246T)	MISSENSE
P99	KRAS	c.38G>A(p.G13D)	MISSENSE
P99	ARID1B	c.4169A>G(p.D1390G	MISSENSE
P99	AXL	c.874dupC(p.H292Pfs	FRAMESHIFT
P99	FLT3	c.106G>A(p.V36I)	MISSENSE
P99	B2M	c.45_48delTTCT(p.S1(	FRAMESHIFT
P99	RNF43	c.1976delG(p.G659Vf	FRAMESHIFT
P99	RNF43	c.902C>A(p.P301H)	MISSENSE
P99	ARID2	c.2119G>A(p.A707T)	MISSENSE
P99	PIK3R1	c.244delA(p.I82Sfs*3:	FRAMESHIFT
P99	AXL	c.195dupC(p.E66Rfs*.	FRAMESHIFT
P100	APC	c.4147dupA(p.M1383	FRAMESHIFT
P100	CYLD	c.2758C>G(p.L920V)	MISSENSE
P100	LRP1B	c.10912C>T(p.R3638\	MISSENSE
P100	TP53	c.404G>A(p.C135Y)	MISSENSE
P100	APC	c.532-1G>C	SPLICE
P101	CDKN1C	c.190G>T(p.D64Y)	MISSENSE
P101	PIK3CA		Amplication
P101	EGFR	c.2303G>A(p.S768N)	MISSENSE
P101	NFE2L2	c.235G>C(p.E79Q)	MISSENSE
P101	AR	c.277G>A(p.E93K)	MISSENSE
P102	KRAS	c.35G>A(p.G12D)	MISSENSE
P102	TP53	c.733G>A(p.G245S)	MISSENSE
P102	APC	c.3919delA(p.I1307*)	FRAMESHIFT
P102	ERBB3	c.1064C>T(p.T355I)	MISSENSE
P102	APC	c.3940_3943dupAGG	FRAMESHIFT
P102	FBXW7	c.931T>C(p.W311R)	MISSENSE
P102	FANCD2	c.3095A>G(p.N1032S	MISSENSE
P103	TP53	c.742C>T(p.R248W)	MISSENSE
P103	APC	c.4198delT(p.S1400Rf	FRAMESHIFT
P103	ERCC5	c.1951G>A(p.D651N)	MISSENSE
P103	PTK2		Amplication
P103	MYC		Amplication
P103	EWSR1	c.1279G>A(p.V427M)	MISSENSE
P103	PLCB4	c.2890A>G(p.I964V)	MISSENSE
P103	KRAS	c.35G>A(p.G12D)	MISSENSE
P104	TOP1		Amplication
P104	FLT4	c.1309C>T(p.R437C)	MISSENSE
P104	FLT1	c.2603C>T(p.T868M)	MISSENSE

P104	ZNF217		Amplication
P104	GNAS		Amplication
P104	TP53	c.129delGinsAA(p.M4	FRAMESHIFT
P104	APC	c.1861dupA(p.T621N	FRAMESHIFT
P104	SRC		Amplication
P104	FGFR1		Amplication
P104	CTNNB1	c.1522A>C(p.K508Q)	MISSENSE
P105	TP53	c.216dupC(p.V73Rfs*	FRAMESHIFT
P105	ZNF217		Amplication
P105	APC	c.646C>T(p.R216*)	STOP GAINED
P105	TOP1		Amplication
P105	GNAS		Amplication
P105	TOP1		Amplication
P105	ZNF217		Amplication
P105	GNAS		Amplication
P106	FGFR1		Amplication
P106	AR	c.1615C>T(p.R539C)	MISSENSE
P106	APC	c.4666dupA(p.T1556I	FRAMESHIFT
P106	ARID1A	c.2988+1G>A	SPLICE
P106	ESR1	c.146C>T(p.P49L)	MISSENSE
P106	ESR1	c.1507C>T(p.R503W)	MISSENSE
P106	KRAS	c.34G>T(p.G12C)	MISSENSE
P106	TP53	c.743G>A(p.R248Q)	MISSENSE
P107	EGFR	EGFR:exon4~VOPP1:e	FUSION
P107	SMAD4	c.1577A>T(p.E526V)	MISSENSE
P107	FLT4	c.2630C>A(p.A877D)	MISSENSE
P107	EGFR		Amplication
P107	ATM	c.3019G>A(p.D1007N	MISSENSE
P107	EGFR	EGFR:exon25~SEPT14	FUSION
P107	APC	c.4037C>A(p.S1346*)	STOP GAINED
P107	APC	c.3842C>A(p.S1281*)	STOP GAINED
P107	ATRX	c.5566+1G>T	SPLICE
P107	TP53	c.733G>A(p.G245S)	MISSENSE
P107	EGFR		Amplication
P108	AMER1	c.1921C>T(p.R641*)	STOP GAINED
P108	INPP4B	c.1704T>G(p.I568M)	MISSENSE
P108	SMAD2	c.1387delT(p.C463Afs	FRAMESHIFT
P108	SMAD2	c.1279C>T(p.R427*)	STOP GAINED
P108	KRAS	c.35G>T(p.G12V)	MISSENSE
P108	SMAD3	c.1102C>T(p.R368*)	STOP GAINED
P108	APC	c.4348C>T(p.R1450*)	STOP GAINED
P108	APC	c.1548+1G>A	SPLICE
P108	PAK3	c.611G>A(p.R204H)	MISSENSE
P109	CTNNB1	c.199G>A(p.E67K)	MISSENSE

P109	TP53	c.733G>A(p.G245S)	MISSENSE
P109	NRAS	c.181C>A(p.Q61K)	MISSENSE
P109	APC	c.1495C>T(p.R499*)	STOP GAINED
P109	ALK	c.2144G>A(p.G715E)	MISSENSE
P109	FLCN	c.1429C>T(p.R477*)	STOP GAINED
P109	APC	c.688_692delCGTAT(p	FRAMESHIFT
P110	KRAS	c.37G>C(p.G13R)	MISSENSE
P110	RAD50	FSD1L:exon1~RAD50:	FUSION
P110	C11orf30	c.3130G>C(p.V1044L)	MISSENSE
P110	PIK3CA	c.1634A>G(p.E545G)	MISSENSE
P110	APC	c.4473dupT(p.A1492(	FRAMESHIFT
P110	TP53	c.380C>T(p.S127F)	MISSENSE
P110	APC	c.2475T>A(p.Y825*)	STOP GAINED
P110	FGFR3		Amplication
P111	XPC	c.991-2A>T	SPLICE
P111	POLE	c.5648C>T(p.A1883V)	MISSENSE
P111	CDK12	c.2336G>A(p.R779H)	MISSENSE
P111	PREX2	c.1835A>G(p.K612R)	MISSENSE
P111	EPHA3	c.2849A>G(p.D950G)	MISSENSE
P111	ARAF	c.563G>A(p.R188H)	MISSENSE
P111	RPTOR	c.1336C>T(p.R446W)	MISSENSE
P111	KRAS	c.38G>A(p.G13D)	MISSENSE
P111	PGR	c.2404T>G(p.W802G)	MISSENSE
P111	PRSS1	c.134A>C(p.Y45S)	MISSENSE
P111	CTCF	c.134C>T(p.T45M)	MISSENSE
P111	MED12	c.2770C>A(p.L924M)	MISSENSE
P111	AIP	c.332G>A(p.G111D)	MISSENSE
P111	BRCA2	c.1813dupA(p.I605Nf	FRAMESHIFT
P111	KMT2A	c.2318delC(p.P773Rfs	FRAMESHIFT
P111	PBRM1	c.1600C>T(p.R534*)	STOP GAINED
P111	RB1	c.137+1G>A	SPLICE
P111	AMER1	c.2908C>T(p.P970S)	MISSENSE
P111	FANCC	c.1082G>A(p.R361Q)	MISSENSE
P111	TSC2	c.4727C>T(p.T1576M	MISSENSE
P111	KMT2B	c.8132G>A(p.R2711H	MISSENSE
P111	FBXW7	c.2065C>T(p.R689W)	MISSENSE
P111	GRIN2A	c.1510C>T(p.R504W)	MISSENSE
P111	APC	c.7811A>C(p.N2604T)	MISSENSE
P111	NOTCH1	c.4249C>A(p.P1417T)	MISSENSE
P111	GRIN2A	c.3238C>T(p.H1080Y)	MISSENSE
P111	MSH6	c.3261delC(p.F1088Sf	FRAMESHIFT
P111	KMT2A	c.6190C>T(p.R2064C)	MISSENSE
P111	PTCH1	c.1274C>T(p.T425M)	MISSENSE
P111	NKX2-1	c.90G>T(p.E30D)	MISSENSE

P111	PKHD1	c.8141G>A(p.R2714Q	MISSENSE
P111	EPHA2	c.524G>A(p.R175H)	MISSENSE
P111	ALK	c.83G>A(p.R28H)	MISSENSE
P111	AMER1	c.1801C>T(p.R601*)	STOP GAINED
P111	GNAS	c.139G>A(p.E47K)	MISSENSE
P111	FOXP1	c.1154T>A(p.L385Q)	MISSENSE
P111	BRD4	c.137delC(p.P46Rfs*4	FRAMESHIFT
P111	IGF2	c.518dupC(p.E174Rfs	FRAMESHIFT
P111	ZNF703	c.61G>A(p.G21S)	MISSENSE
P111	GRM8	c.959A>G(p.Q320R)	MISSENSE
P111	LRP1B	c.9325A>G(p.R3109G	MISSENSE
P111	CASP8	c.1288C>T(p.R430*)	STOP GAINED
P111	GRM3	c.1079A>G(p.Q360R)	MISSENSE
P111	FANCA	c.4303G>A(p.A1435T	MISSENSE
P111	GRM3	c.1241A>G(p.N414S)	MISSENSE
P111	RECQL4	c.385C>T(p.P129S)	MISSENSE
P111	FOXP1	c.1540C>T(p.R514C)	MISSENSE
P111	ERBB4	c.946G>A(p.E316K)	MISSENSE
P111	ATM	c.1661C>T(p.T554M)	MISSENSE
P111	BAP1	c.862G>A(p.A288T)	MISSENSE
P111	DOT1L	c.3158C>T(p.A1053V)	MISSENSE
P111	APC	c.4438C>T(p.Q1480*)	STOP GAINED
P111	NSD1	c.7697C>T(p.T2566I)	MISSENSE
P111	CDK12	c.4177G>T(p.G1393W	MISSENSE
P111	PLK1	c.1711G>A(p.G571S)	MISSENSE
P111	FAT1	c.9683dupC(p.V3229(	FRAMESHIFT
P111	APC	c.694C>T(p.R232*)	STOP GAINED
P111	PTPN13	c.3069-2A>G	SPLICE
P111	KDM5A	c.4234G>T(p.G1412C	MISSENSE
P111	TERT	c.2776G>A(p.G926S)	MISSENSE
P111	LRP1B	c.1675C>T(p.R559C)	MISSENSE
P111	PKHD1	c.3385G>A(p.G1129R	MISSENSE
P111	CYP2D6	c.508C>T(p.R170C)	MISSENSE
P111	KLLN	c.238A>C(p.S80R)	MISSENSE
P111	DNMT3A	c.2149A>G(p.N717D)	MISSENSE
P111	JAK1	c.2580delA(p.K860Nf	FRAMESHIFT
P111	RNF43	c.1976delG(p.G659Vf	FRAMESHIFT
P112	DDR2	c.778C>T(p.R260W)	MISSENSE
P112	KRAS	c.351A>C(p.K117N)	MISSENSE
P112	FLT4	c.1002C>G(p.S334R)	MISSENSE
P112	FLT3		Amplication
P112	MYC		Amplication
P112	PIK3CA	c.1633G>A(p.E545K)	MISSENSE
P112	FLT1		Amplication

P112	SMARCA4	c.3728G>A(p.R1243Q	MISSENSE
P112	CDC73	c.1480G>A(p.V494I)	MISSENSE
P112	ARID1A	c.1650dupC(p.Y551Lf	FRAMESHIFT
P113	TP53	c.844C>T(p.R282W)	MISSENSE
P113	PIK3CA	c.328_330delGAA(p.E	INFRAME INDEL
P113	KRAS	c.35G>T(p.G12V)	MISSENSE
P113	APC	c.1847delT(p.L616Wf	FRAMESHIFT
P113	APC	c.4241delT(p.V1414E	FRAMESHIFT
P114	APC	c.2626C>T(p.R876*)	STOP GAINED
P114	PIK3CA	c.353G>A(p.G118D)	MISSENSE
P114	APC	c.4666dupA(p.T1556I	FRAMESHIFT
P114	AMER1	c.1150G>T(p.E384*)	STOP GAINED
P114	PPP2R1A	c.1051G>A(p.G351S)	MISSENSE
P114	PRF1	c.1648C>T(p.R550W)	MISSENSE
P114	TP53	c.659A>G(p.Y220C)	MISSENSE
P114	LZTR1	c.1260+2_1260+8del	SPLICE
P114	KRAS	c.35G>A(p.G12D)	MISSENSE
P115	KRAS	c.35G>A(p.G12D)	MISSENSE
P115	APC	c.646C>T(p.R216*)	STOP GAINED
P115	ARID1A	c.1020_1031delGGCG	INFRAME INDEL
P115	CASP8	c.1252C>G(p.L418V)	MISSENSE
P115	SMAD4	c.1082G>A(p.R361H)	MISSENSE
P115	FBXW7	c.832C>T(p.R278*)	STOP GAINED
P115	ARID2	c.5371_5372delAA(p.	FRAMESHIFT
P115	PRKCI	c.1438C>T(p.R480C)	MISSENSE
P115	MED12	c.4036C>T(p.R1346C)	MISSENSE
P115	APC	c.3927_3931delAAAG	FRAMESHIFT
P116	AXL	c.502C>G(p.P168A)	MISSENSE
P116	BRD4	c.3197T>G(p.L1066R)	MISSENSE
P116	BMPR1A	c.905G>T(p.W302L)	MISSENSE
P116	PRF1	c.1537A>C(p.N513H)	MISSENSE
P116	FANCM	IGR (downstream RPL	FUSION
P116	APC	c.2815A>T(p.K939*)	STOP GAINED
P116	PLCB4	c.1629_1630invAG(p.	MISSENSE
P116	SDHB	c.807G>C(p.M269I)	MISSENSE
P116	KRAS	c.35G>T(p.G12V)	MISSENSE
P116	XPC	c.1425delG(p.T476Qf	FRAMESHIFT
P116	APC	c.4285C>T(p.Q1429*)	STOP GAINED
P116	TERT	c.-124C>T	MISSENSE
P116	AR	c.1025C>T(p.P342L)	MISSENSE
P116	AR	c.2290T>A(p.Y764N)	MISSENSE
P116	AR	c.2272G>A(p.V758I)	MISSENSE
P117	NF1	c.2001+2T>C	SPLICE
P117	TP53	c.492G>T(p.K164N)	MISSENSE

P117	PDK1	c.1292C>T(p.T431M)	MISSENSE
P117	PBRM1	c.1847A>C(p.K616T)	MISSENSE
P117	CHD8	c.4457T>C(p.I1486T)	MISSENSE
P117	ERCC4	c.2215G>A(p.G739S)	MISSENSE
P117	TEK	c.2232delG(p.K745Rf	FRAMESHIFT
P117	NQO1	c.746delA(p.N249Tfs	FRAMESHIFT
P117	CASP8	c.1337T>C(p.L446P)	MISSENSE
P117	POLE	c.1676G>A(p.R559Q)	MISSENSE
P117	PDGFRA	c.493G>T(p.E165*)	STOP GAINED
P117	KMT2B	c.7921G>A(p.A2641T	MISSENSE
P117	DPYD	c.893C>T(p.T298M)	MISSENSE
P117	PTPN13	c.4160A>G(p.D1387G	MISSENSE
P117	MTOR	c.6725A>G(p.H2242R	MISSENSE
P117	FANCA	c.926G>A(p.S309N)	MISSENSE
P117	CYP2C9	c.1003C>T(p.R335W)	MISSENSE
P117	CBLB	c.1787G>T(p.R596L)	MISSENSE
P117	EXT2	c.28C>T(p.R10W)	MISSENSE
P117	TET2	c.3770C>T(p.T1257M	MISSENSE
P117	POLE	c.6842A>G(p.N2281S	MISSENSE
P117	MLH3	c.1755dupA(p.E586Rf	FRAMESHIFT
P117	EXT1	c.369delA(p.E125Rfs*	FRAMESHIFT
P117	GATA6	c.1087C>T(p.Q363*)	STOP GAINED
P117	ASXL1	c.1085+1G>A	SPLICE
P117	IGF1R	c.419C>T(p.A140V)	MISSENSE
P117	RAC3	c.73A>G(p.T25A)	MISSENSE
P117	NOTCH2	c.1499G>T(p.S500I)	MISSENSE
P117	ZNF703	c.1316G>A(p.G439D)	MISSENSE
P117	RET	c.2657G>A(p.R886Q)	MISSENSE
P117	AMER1	c.519delT(p.F173Lfs*	FRAMESHIFT
P117	MLH3	c.1755delA(p.E586Nf	FRAMESHIFT
P117	IGF1R	c.1636T>C(p.S546P)	MISSENSE
P117	TSC2	c.2416G>A(p.V806M)	MISSENSE
P117	TP53	c.817C>T(p.R273C)	MISSENSE
P117	SGK1	c.1148G>A(p.R383Q)	MISSENSE
P117	NOTCH3	c.4999C>T(p.R1667C)	MISSENSE
P117	FAT1	c.4355A>G(p.H1452R	MISSENSE
P117	CXCR4	c.739G>A(p.A247T)	MISSENSE
P117	BRCA2	c.9097delA(p.T3033Lf	FRAMESHIFT
P117	AXL	c.1010delC(p.P337Lfs	FRAMESHIFT
P117	CREBBP	c.1270C>T(p.R424*)	STOP GAINED
P117	ESR1	c.425G>A(p.R142H)	MISSENSE
P117	CDKN1C	c.91C>A(p.R31S)	MISSENSE
P117	GRM8	c.2309C>T(p.T770M)	MISSENSE
P117	NOTCH2	c.1259C>T(p.A420V)	MISSENSE

P117	PTK2	c.2825C>T(p.T942M)	MISSENSE
P117	PKHD1	c.10388T>C(p.L3463S	MISSENSE
P117	MDM4	c.535G>A(p.A179T)	MISSENSE
P117	RECQL4	c.3011C>T(p.A1004V)	MISSENSE
P117	POLE	c.100C>T(p.R34C)	MISSENSE
P117	MSH2	c.2605G>A(p.A869T)	MISSENSE
P117	TP53	c.604C>T(p.R202C)	MISSENSE
P117	ALK	c.3178C>T(p.R1060C)	MISSENSE
P117	MSH6	c.718C>T(p.R240*)	STOP GAINED
P117	ARID1B	c.3890C>T(p.T1297M	MISSENSE
P117	TOP2A	c.4303A>G(p.R1435G	MISSENSE
P117	THADA	c.1783G>T(p.G595*)	STOP GAINED
P117	PTEN	c.253+2T>C	SPLICE
P117	NOTCH1	c.7327G>A(p.V2443M	MISSENSE
P117	DOT1L	c.146C>T(p.P49L)	MISSENSE
P117	EGFR	c.2320G>A(p.V774M)	MISSENSE
P117	GATA3	c.379C>T(p.P127S)	MISSENSE
P117	FLT4	c.3208C>T(p.R1070C)	MISSENSE
P117	PDE11A	c.475C>T(p.R159W)	MISSENSE
P117	TET2	c.4351C>T(p.R1451W	MISSENSE
P117	PPP2R1A	c.548G>A(p.R183Q)	MISSENSE
P117	FLT1	c.406G>A(p.V136I)	MISSENSE
P117	FANCA	c.3788_3790delTCT(p	INFRAME INDEL
P117	LRP1B	c.12869A>C(p.E4290A	MISSENSE
P117	DENND1A	c.2969C>T(p.A990V)	MISSENSE
P117	CTNNB1	c.1000G>A(p.E334K)	MISSENSE
P117	ARID2	c.940C>T(p.R314C)	MISSENSE
P117	KMT2B	c.4357A>G(p.S1453G	MISSENSE
P117	EPAS1	c.610A>G(p.N204D)	MISSENSE
P117	AXIN2	c.1883G>T(p.R628L)	MISSENSE
P117	TTF1	c.836dupA(p.K280Efs	FRAMESHIFT
P117	TGFBR2	c.382_383delAA(p.K1	FRAMESHIFT
P117	PMS1	c.418+2T>C	SPLICE
P117	ALK	c.1918G>A(p.G640R)	MISSENSE
P117	SUFU	c.1274C>T(p.A425V)	MISSENSE
P117	SPRY4	c.815T>A(p.L272H)	MISSENSE
P117	STAG2	c.1642C>T(p.L548F)	MISSENSE
P117	POLD1	c.2096C>T(p.S699F)	MISSENSE
P117	NOTCH3	c.5006G>A(p.R1669H	MISSENSE
P117	PTCH1	c.3606dupC(p.S1203C	FRAMESHIFT
P117	APC	c.4348C>T(p.R1450*)	STOP GAINED
P117	FBXW7	c.1094G>A(p.W365*)	STOP GAINED
P117	ERCC4	c.289C>T(p.R97C)	MISSENSE
P117	LRP1B	c.2803G>A(p.G935R)	MISSENSE

P117	EPHA2	c.1703G>A(p.R568H)	MISSENSE
P117	FLT3	c.1349C>T(p.A450V)	MISSENSE
P117	ALK	c.4522G>A(p.G1508S)	MISSENSE
P117	FGF19	c.452G>A(p.R151Q)	MISSENSE
P117	DOT1L	c.2680G>A(p.A894T)	MISSENSE
P117	SDHA	c.1771G>A(p.A591T)	MISSENSE
P117	CCND1	c.842T>C(p.V281A)	MISSENSE
P117	BAX	c.121delG(p.E41Rfs*1	FRAMESHIFT
P118	SMAD4	c.1610A>G(p.D537G)	MISSENSE
P118	APC	c.3146G>A(p.W1049*	STOP GAINED
P118	PIK3CA	c.3140A>G(p.H1047R	MISSENSE
P118	VEGFA	c.641G>A(p.G214E)	MISSENSE
P118	TP53	c.742C>T(p.R248W)	MISSENSE
P118	KRAS	c.35G>A(p.G12D)	MISSENSE
P118	APC	c.4260_4261delCA(p.	FRAMESHIFT
P118	TUBB3	c.904G>A(p.A302T)	MISSENSE
P118	FLT1	c.842G>A(p.R281Q)	MISSENSE
P118	CASP8	c.1232C>T(p.P411L)	MISSENSE
P119	IFNB1	c.305A>C(p.E102A)	MISSENSE
P119	ARID1A	c.688delG(p.A230Rfs*	FRAMESHIFT
P119	BRAF	c.784C>A(p.Q262K)	MISSENSE
P119	PKHD1	c.6951G>T(p.L2317F)	MISSENSE
P119	APC	c.2825delA(p.N942Ifs	FRAMESHIFT
P119	NRAS	c.181C>A(p.Q61K)	MISSENSE
P119	SMAD2	c.1395C>A(p.S465R)	MISSENSE
P119	CDC73	c.1208C>G(p.T403S)	MISSENSE
P119	APC	c.4393_4394delAG(p.	FRAMESHIFT
P119	ARID1B	c.6413T>G(p.L2138R)	MISSENSE
P119	AMER1	c.1372_1373delAG(p.	FRAMESHIFT
P119	TAP2	c.1187T>C(p.L396P)	MISSENSE
P120	TP53	c.1052delA(p.K351Rfs	FRAMESHIFT
P120	FBXW7	c.1739A>G(p.H580R)	MISSENSE
P120	KRAS	c.35G>A(p.G12D)	MISSENSE
P120	SMARCA4	c.2681C>T(p.T894M)	MISSENSE
P120	APC	c.3927_3931delAAAG	FRAMESHIFT
P120	TP53	c.844C>T(p.R282W)	MISSENSE
P120	VEGFA		Amplication
P120	APC	c.3055G>T(p.G1019X)	STOP GAINED
P120	GRIN2A	c.2084G>A(p.R695Q)	MISSENSE
P120	ERBB2	c.2033G>A(p.R678Q)	MISSENSE
P121	TUBB4A	c.811G>A(p.A271T)	MISSENSE
P121	TEK	c.2957C>T(p.S986F)	MISSENSE
P121	TP53	c.1010G>T(p.R337L)	MISSENSE
P121	CTNNB1	c.12_215del	LARGE FRAGMENT D

P122	KRAS	c.35G>C(p.G12A)	MISSENSE
P122	STK11	c.998G>A(p.R333H)	MISSENSE
P122	APC	EPB41L4A:exon9~APC:e	FUSION
P122	TP53	c.227_279del(p.A76Vfs	FRAMESHIFT
P122	SMAD4	c.1081C>T(p.R361C)	MISSENSE
P123	TP53	c.524G>A(p.R175H)	MISSENSE
P123	APC	c.3957dupT(p.V1320Cfs	FRAMESHIFT
P123	PTEN		Deletion
P123	NRAS	c.182A>G(p.Q61R)	MISSENSE
P123	PTEN		Deletion
P124	SMAD4	SMAD4:exon5~IGR (d	FUSION
P124	EZH2	c.758C>A(p.P253Q)	MISSENSE
P124	KMT2A	c.8264T>C(p.I2755T)	MISSENSE
P124	DICER1	c.3661A>T(p.N1221Y)	MISSENSE
P124	NOTCH1	c.1270G>A(p.E424K)	MISSENSE
P124	TP53	c.690delC(p.T231Pfs*	FRAMESHIFT
P124	KDR	c.2571C>A(p.D857E)	MISSENSE
P124	KRAS	c.35G>A(p.G12D)	MISSENSE
P124	FAT1	c.4035G>A(p.W1345*	STOP GAINED
P125	PIK3CA	c.353G>A(p.G118D)	MISSENSE
P125	APC	c.1659delG(p.W553Cfs	FRAMESHIFT
P125	DDR2	c.1894C>G(p.L632V)	MISSENSE
P125	APC	c.4666dupA(p.T1556Nf	FRAMESHIFT
P125	NTRK3	c.1934G>A(p.R645H)	MISSENSE
P125	AMER1	c.368T>A(p.L123*)	STOP GAINED
P125	KRAS	c.38G>A(p.G13D)	MISSENSE
P125	TP53	c.558_559delTG(p.D18	FRAMESHIFT
P126	ERBB4	c.3222A>C(p.E1074D)	MISSENSE
P126	APC	c.4216C>T(p.Q1406*)	STOP GAINED
P126	CDK12	CDK12:exon1~RPTOR	FUSION
P126	TP53	c.272G>A(p.W91*)	STOP GAINED
P127	MLLT3	c.1049C>G(p.S350X)	STOP GAINED
P127	CDH1	c.1320G>T(p.K440N)	MISSENSE
P127	KRAS	c.436G>A(p.A146T)	MISSENSE
P127	IGF1R	c.2278G>A(p.A760T)	MISSENSE
P127	BRD4	c.3796G>A(p.E1266K)	MISSENSE
P127	TP53	c.366_373dupGACTTGC	FRAMESHIFT
P127	MAX	c.453C>A(p.S151R)	MISSENSE
P127	NRG1	c.805T>C(p.Y269H)	MISSENSE
P127	AXIN2	c.799_800delGT(p.V267	FRAMESHIFT
P127	RARA	c.1072G>T(p.A358S)	MISSENSE
P127	ESR1	c.338C>T(p.P113L)	MISSENSE
P127	SMAD2	c.215C>A(p.T72N)	MISSENSE
P127	CREBBP	c.1714G>T(p.G572X)	STOP GAINED
P127	EP300	c.3590G>T(p.R1197M)	MISSENSE
P127	ROS1	c.1792T>A(p.F598I)	MISSENSE

P128	PIK3R1	c.1392_1403delTAGAT	INFRAME INDEL
P128	NTRK1	c.310C>T(p.R104C)	MISSENSE
P128	KRAS	c.38G>A(p.G13D)	MISSENSE
P128	MED12	c.117_134delGAATGTA	INFRAME INDEL
P128	SMAD4	c.1082G>A(p.R361H)	MISSENSE
P128	RNF43	c.1A>T(p.M1?)	START LOST
P128	GNAS	c.1631G>A(p.R544Q)	MISSENSE
P129	CEP57	p.P470H (c.C1409A)	MISSENSE
P129	TP53	p.I195T (c.T584C)	MISSENSE
P129	KRAS	p.G12D (c.G35A)	MISSENSE
P129	APC	p.R1450X (c.C4348T)	STOP GAINED
P129	CEP57	p.P470H (c.C1409A)	MISSENSE
P129	KRAS	p.G12D (c.G35A)	MISSENSE
P129	TP53	p.I195T (c.T584C)	MISSENSE
P129	APC	p.R1450X (c.C4348T)	STOP GAINED
P130	DPYD	c.299T>C(p.F100S)	MISSENSE
P130	KRAS	c.35G>T(p.G12V)	MISSENSE
P130	APC	c.675_680delAAAGGA(	INFRAME INDEL
P130	PTPRD	c.4987A>G(p.S1663G)	MISSENSE
P130	APC	c.2083C>T(p.Q695*)	STOP GAINED
P130	FBXW7	c.1513C>T(p.R505C)	MISSENSE
P130	FBXW7	c.832C>T(p.R278*)	STOP GAINED
P130	PIK3CA	c.1633G>A(p.E545K)	MISSENSE
P130	SMAD4	c.1067C>T(p.P356L)	MISSENSE
P130	APC	c.4348C>T(p.R1450*)	STOP GAINED
P130	IFNGR1	c.45C>G(p.S15R)	MISSENSE
P130	STAG2	c.1865A>C(p.K622T)	MISSENSE
P131	APC	c.4099C>T(p.Q1367X)	STOP GAINED
P131	TNFRSF11A	c.400G>A(p.A134T)	MISSENSE
P131	SMARCB1		Deletion
P131	TP53		Deletion
P131	TP53	c.524G>A(p.R175H)	MISSENSE
P132	BRD4	p.R1130L (c.G3389T)	MISSENSE
P132	CTNNB1	p.Q302fs (c.905delA)	FRAMESHIFT
P132	FANCC	p.T420M (c.C1259T)	MISSENSE
P132	NOTCH2	p.R1931H (c.G5792A)	MISSENSE
P132	POLH	p.R234H (c.G701A)	MISSENSE
P132	SLC3A2	p.K298fs (c.892delA)	FRAMESHIFT
P132	ABCB1	p.V125fs (c.373_374ins	FRAMESHIFT
P132	CDA	p.A101V (c.C302T)	MISSENSE
P132	GNAS	p.D141G (c.A422G)	MISSENSE
P132	STAT3	p.V310M (c.G928A)	MISSENSE
P132	PDGFRB	p.A168T (c.G502A)	MISSENSE
P132	EP300	p.Q1522H (c.G4566T)	MISSENSE
P132	NTRK3	p.D476G (c.A1427G)	MISSENSE
P132	FAT1	p.T874P (c.A2620C)	MISSENSE
P132	CCNE1	p.E45A (c.A134C)	MISSENSE

P132	MDM2	p.P319fs (c.955delC)	FRAMESHIFT
P132	HDAC9	p.R440H (c.G1319A)	MISSENSE
P132	CDK6	p.R214H (c.G641A)	MISSENSE
P132	FANCD2	c.A2495-2G	SPLICE
P132	FBXW7	p.R441Q (c.G1322A)	MISSENSE
P132	APC	p.R1788C (c.C5362T)	MISSENSE
P132	FANCA	p.A1443T (c.G4327A)	MISSENSE
P132	ERBB2IP	p.D947V (c.A2840T)	MISSENSE
P132	ATRX	p.R1518K (c.G4553A)	MISSENSE
P132	MYCN	p.A354V (c.C1061T)	MISSENSE
P132	KDR	p.V665M (c.G1993A)	MISSENSE
P132	MAP3K1	p.R208X (c.C622T)	STOP GAINED
P132	TP53	p.R213Q (c.G638A)	MISSENSE
P132	APC	p.K1462fs (c.4384_438	FRAMESHIFT
P132	SGK1	p.G234X (c.G700T)	STOP GAINED
P132	GRM8	p.359fs (c.1077_1078in	FRAMESHIFT
P132	HGF	c.G747-1T	SPLICE
P132	SETBP1	p.Q1213R (c.A3638G)	MISSENSE
P132	EPHA5	p.C102Y (c.G305A)	MISSENSE
P132	SMARCA4	p.G19D (c.G56A)	MISSENSE
P132	CREBBP	p.R2344W (c.C7030T)	MISSENSE
P132	IKBKE	p.A338V (c.C1013T)	MISSENSE
P132	MLLT4	p.M85V (c.A253G)	MISSENSE
P132	BRCA2	p.G602fs (c.1806delA)	FRAMESHIFT
P132	IDH1	p.I99M (c.A297G)	MISSENSE
P132	PTCH1	p.V1126I (c.G3376A)	MISSENSE
P132	MUTYH	p.R414C (c.C1240T)	MISSENSE
P132	SMO	p.H692fs (c.2076delC)	FRAMESHIFT
P132	MPL	p.H624N (c.C1870A)	MISSENSE
P132	MITF	p.A277T (c.G829A)	MISSENSE
P132	IGF1R	p.R391H (c.G1172A)	MISSENSE
P132	ERCC4	p.F761L (c.T2283G)	MISSENSE
P132	FLT3	p.S633G (c.A1897G)	MISSENSE
P132	NTRK1	p.Y72H (c.T214C)	MISSENSE
P132	RPTOR	p.V596M (c.G1786A)	MISSENSE
P132	ARID1A	p.R1461X (c.C4381T)	STOP GAINED
P132	TTF1	p.K274fs (c.821delA)	FRAMESHIFT
P132	FBXW7	p.G670X (c.G2008T)	STOP GAINED
P132	GATA6	p.G305D (c.G914A)	MISSENSE
P132	GNA11	p.E49K (c.G145A)	MISSENSE
P132	CREBBP	p.D1143G (c.A3428G)	MISSENSE
P132	ASXL1	p.G642fs (c.1926_1927	FRAMESHIFT
P132	AXL	p.63fs (c.189_190insC)	FRAMESHIFT
P132	JARID2	p.M683I (c.G2049A)	MISSENSE
P132	CBLB	p.G661R (c.G1981A)	MISSENSE
P132	TAP1	p.V254A (c.T761C)	MISSENSE
P132	PTPN13	p.P225H (c.C674A)	MISSENSE

P132	FANCL	p.T304A (c.A910G)	MISSENSE
P132	APC	p.R805X (c.C2413T)	STOP GAINED
P132	DNMT3A	p.R209H (c.G626A)	MISSENSE
P132	MTOR	p.R460X (c.C1378T)	STOP GAINED
P132	CTNNB1	p.R542C (c.C1624T)	MISSENSE
P132	KIT	p.P627L (c.C1880T)	MISSENSE
P132	WRN	p.E3fs (c.8delA)	FRAMESHIFT
P132	FANCI	p.1268_1269del (c.380[illegible]	STOP LOST
P132	CBLB	p.A971T (c.G2911A)	MISSENSE
P132	RRM1	p.R499H (c.G1496A)	MISSENSE
P132	NTRK2	p.R734C (c.C2200T)	MISSENSE
P132	RECQL4	p.M748L (c.A2242T)	MISSENSE
P132	JARID2	p.K565R (c.A1694G)	MISSENSE
P132	PDGFRB	p.T369M (c.C1106T)	MISSENSE
P132	NOTCH1	p.R1762W (c.C5284T)	MISSENSE
P132	MAP3K4	p.K158fs (c.472delA)	FRAMESHIFT
P132	MSH6	p.P59S (c.C175T)	MISSENSE
P132	ROS1	p.P1315H (c.C3944A)	MISSENSE
P132	RPTOR	p.A1269T (c.G3805A)	MISSENSE
P132	XPC	p.A783T (c.G2347A)	MISSENSE
P132	MLH3	p.K585fs (c.1755delA)	FRAMESHIFT
P132	RPTOR	p.V1147I (c.G3439A)	MISSENSE
P132	ERBB4	p.S701N (c.G2102A)	MISSENSE
P132	GRIN2A	p.L63P (c.T188C)	MISSENSE
P132	KRAS	p.G13D (c.G38A)	MISSENSE
P132	KDM5A	p.K1199fs (c.3597_359[illegible]	FRAMESHIFT
P132	AXIN2	p.A684V (c.C2051T)	MISSENSE
P132	POLE	p.L2096V (c.C6286G)	MISSENSE
P132	LZTR1	p.L816Q (c.T2447A)	MISSENSE
P132	SMARCB1	p.A109fs (c.327_328del	FRAMESHIFT
P132	SMARCA4	p.A1168T (c.G3502A)	MISSENSE
P132	ATM	p.I2230V (c.A6688G)	MISSENSE
P132	SMARCA4	p.R425Q (c.G1274A)	MISSENSE
P132	PIK3R1	p.G644D (c.G1931A)	MISSENSE
P132	PARK2	p.L112F (c.C334T)	MISSENSE
P132	MGMT	p.L2P (c.T5C)	MISSENSE
P132	TP53	p.R158H (c.G473A)	MISSENSE
P132	TTF1	p.H285R (c.A854G)	MISSENSE
P132	ATR	p.A1570T (c.G4708A)	MISSENSE
P132	C11ORF30	p.G923D (c.G2768A)	MISSENSE
P132	MAP3K4	p.R346C (c.C1036T)	MISSENSE
P132	PALB2	p.M296fs (c.886delA)	FRAMESHIFT
P132	BAX	p.M38fs (c.114delG)	FRAMESHIFT
P132	TOP2A	p.P8H (c.C23A)	MISSENSE
P132	RAF1	p.A142V (c.C425T)	MISSENSE
P132	FAT1	p.V1783M (c.G5347A)	MISSENSE
P132	CDKN1A	p.F51V (c.T151G)	MISSENSE

P132	DUSP2	p.A64T (c.G190A)	MISSENSE
P133	NF1	p.D1313V (c.A3938T)	MISSENSE
P133	PGR	p.C480S (c.G1439C)	MISSENSE
P133	APC	p.S1327X (c.C3980G)	STOP GAINED
P133	APC	p.Q1406X (c.C4216T)	STOP GAINED
P133	ESR1	p.P333S (c.C997T)	MISSENSE
P133	HNF1A	p.S225C (c.A673T)	MISSENSE
P133	KRAS	p.Q61R (c.A182G)	MISSENSE
P133	PDGFRB	p.A789S (c.G2365T)	MISSENSE
P133	TP53	p.R273H (c.G818A)	MISSENSE
P133	FLT4	p.G276D (c.G827A)	MISSENSE
