# Supplementary material for: Comprehensive genomic profiling of colorectal cancer patients reveals differences in mutational landscapes among clinical and pathological subgroups
Source: Front Oncol. 2022 Nov 10;12:1000146. doi: 10.3389/fonc.2022.1000146 (PMC9685809; doi:10.3389/fonc.2022.1000146)

Table S3. The tumor location, tumor volume and stage were not associated with gender and age. Fisher's exact test was used for p value calculation between two groups. Chi-square test was used for p value calculation among three groups.

Features		Gender		p value	Age		p value
		Male	Female		≥ 50	< 50	
Tumor location	Left-side colon	23 (63.89%)	13 (36.11%)	0.161	25 (69.44%)	11 (30.56%)	0.302
	Right-side colon	13 (41.94%)	18 (58.06%)		21 (67.74%)	10 (32.26%)	
	Rectal	39 (59.09%)	27 (40.91%)		53 (80.30%)	13 (19.70%)	
Tumor volume	≥ 5 cm	27 (50.94%)	26 (49.06%)	0.367	39 (73.58%)	14 (26.42%)	1.000
	< 5 cm	46 (60.53%)	30 (39.47%)		57 (75.00%)	19 (25.00%)	
Stage	0~II	35 (56.45%)	27 (43.55%)	>0.999	49 (79.03%)	13 (20.97%)	0.320
	III~IV	40 (56.34%)	31 (43.66%)		50 (70.42%)	21 (29.58%)	
MS status	MSS	67 (55.37%)	54 (44.63%)	0.550	97 (80.17%)	24 (19.83%)	< 0.001
	MSI	8 (66.67%)	4 (33.33%)		2 (16.67%)	10 (83.33%)	

Table S4. The distribution of *KRAS* G12 and G13 mutations in tumors of different anatomic locations.

Locations	KRAS G13	KRAS G12	P value
Right-side colon	9 (39.13%)	10 (43.48%)	
Left-side colon	1 (5.88%)	13 (76.47%)	0.014
Rectal	5 (16.13%)	24 (77.42%)	

Table S5. Clinicopathological features of the validation cohort.

Characteristics	No. (%)
**No. of patients**	240
**Median age, years (range)**	60 (19, 84)
**Sex**	
**Female**	104 (43.3)
**Male**	136 (56.7)
**Primary tumor location**	
**Left-sided colon**	91 (37.92)
**Rectal**	62 (25.83)
**Right-sided colon**	87 (36.2)
**Pathological stage**	
**II**	112 (46.7)
**III**	128 (53.3)
**Histological type**	
**Adenocarcinoma**	212 (88.3)
**Mucinous/signet-ring carcinioma**	28 (11.7)
**Histological grade**	
**Moderate/well**	180 (75.0)
**Poor**	60 (25.0)
**MSI status**	
**MSI-H**	23 (9.6)
**MSI-L/MSS**	217 (90.4)

Figure S1. The comparison of gene and pathway alterations in early- and late-onset patients. (A)The comparison of somatic mutations between early- and late-onset CRC patients. (B)The comparison of pathway alterations between early- and late-onset CRC patients. *: p<0.05, **: p<0.01, ***: p<0.001, ****: p<0.0001.

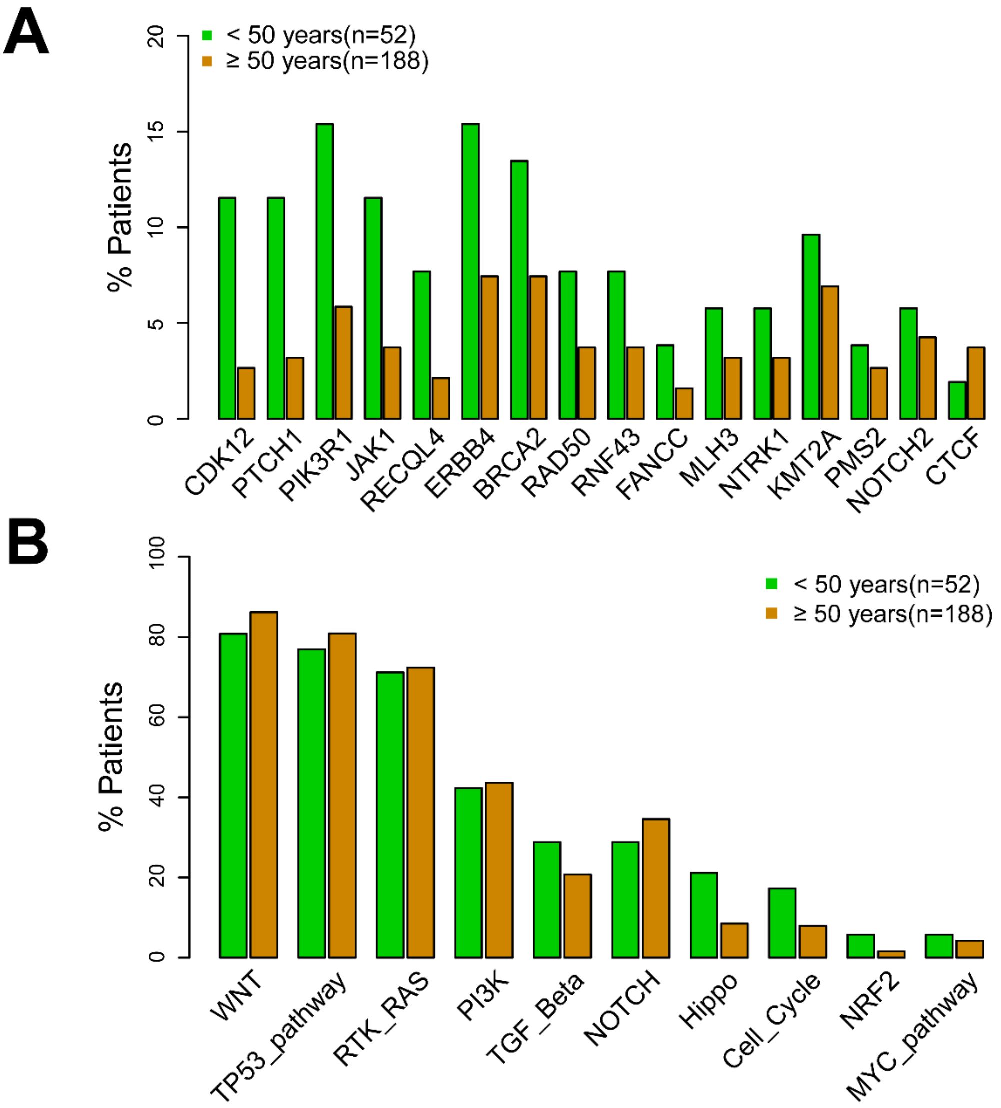

Figure S2. The comparison of genetic alterations in MSS and MSI patients. *: p<0.05, **: p<0.01, ***: p<0.001, ****: p<0.0001.

A. Comparison of MRR genes in Our Cohort

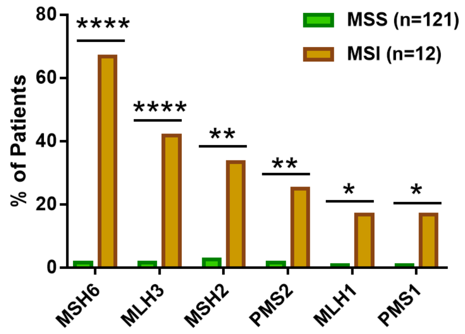

B. Comparison of Mutation Signatures in Our Cohort

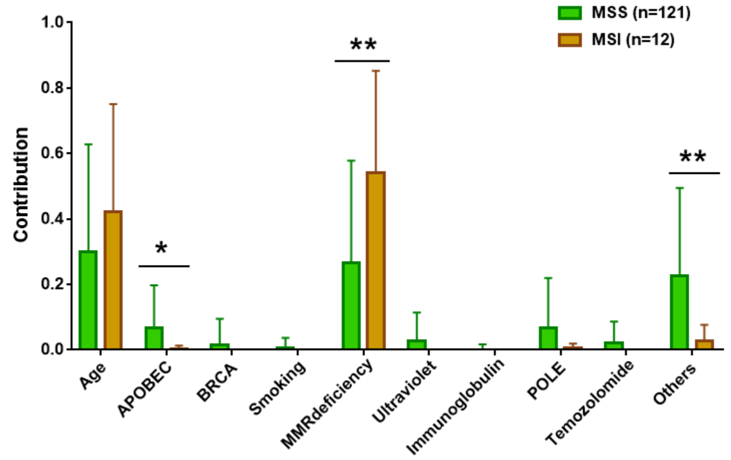

C. Comparison of Mutation Signatures in the Validation Cohort

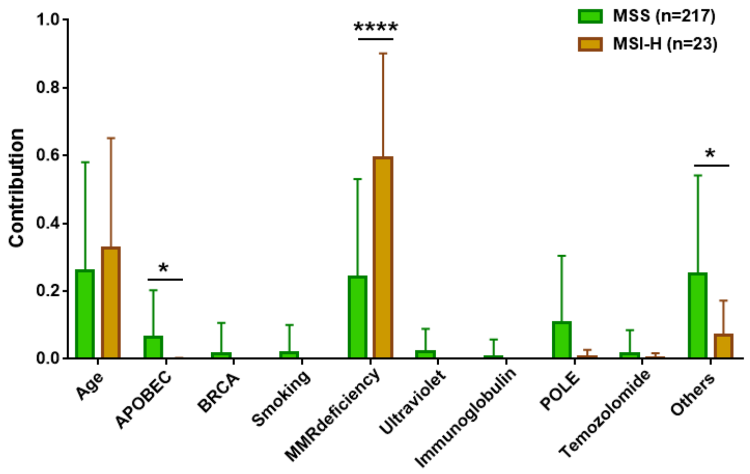

Figure S3. The comparison of gene and pathway alterations among patients with different tumor anatomic locations. (A)The comparison of somatic among patients with different tumor anatomic locations. (B)The comparison of pathway alterations among patients with different tumor anatomic locations. *: p<0.05, **: p<0.01, ***: p<0.001, ****: p<0.0001.

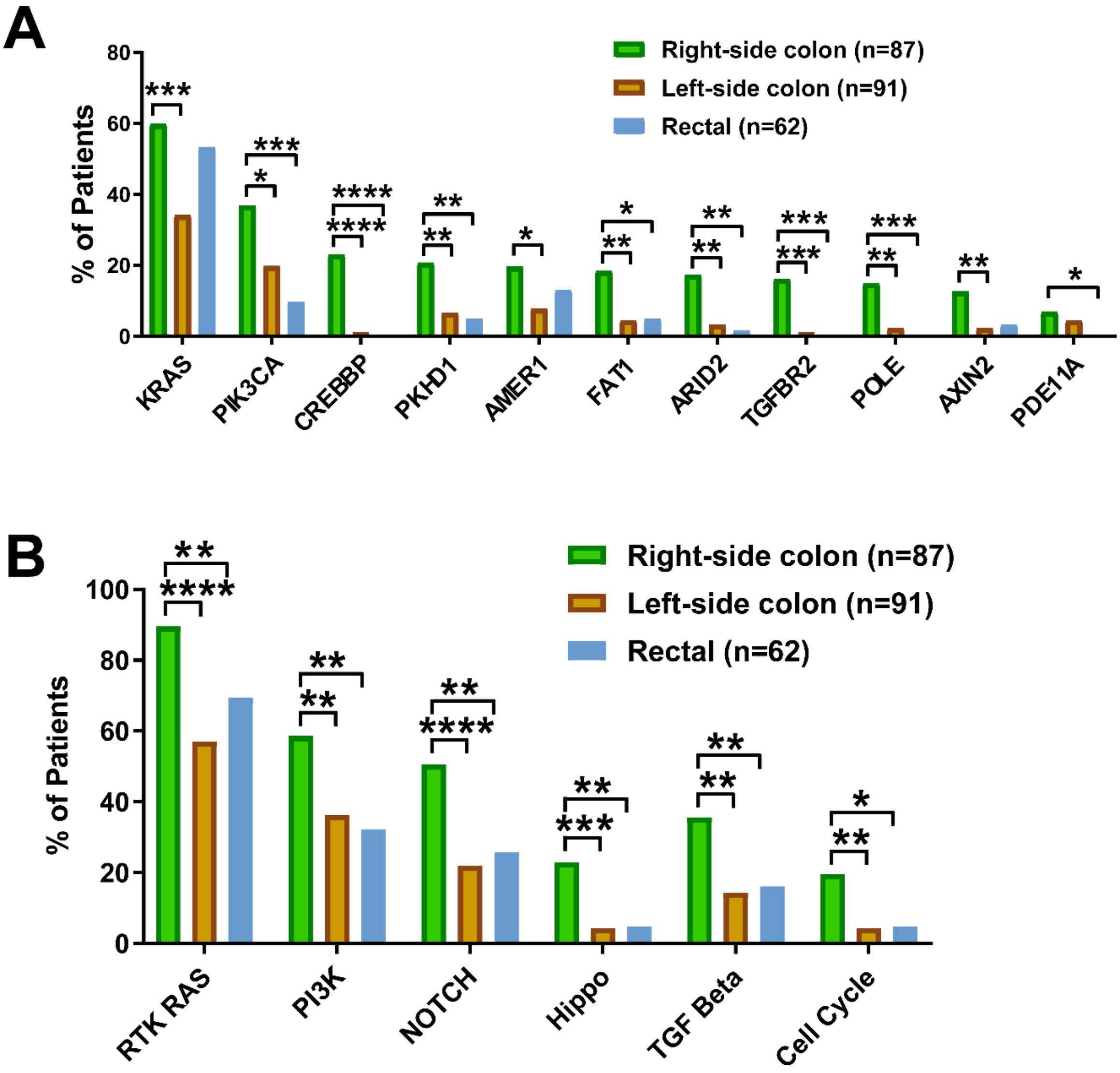

Supplement: Supplementary file 3 [file DataSheet_3.pdf]
